# Principal component-based clinical aging clocks identify signatures of healthy aging and targets for clinical intervention

**DOI:** 10.1038/s43587-024-00646-8

**Published:** 2024-06-19

**Authors:** Sheng Fong, Kamil Pabis, Djakim Latumalea, Nomuundari Dugersuren, Maximilian Unfried, Nicholas Tolwinski, Brian Kennedy, Jan Gruber

**Affiliations:** 1 Department of Geriatric Medicine, Singapore General Hospital, Singapore, Singapore; 2 Clinical and Translational Sciences PhD Program, Duke-NUS Medical School, Singapore, Singapore; 3 Healthy Longevity Translational Research Program, Yong Loo Lin School of Medicine, National University of Singapore, Singapore, Singapore; 4 Center for Healthy Longevity, National University Health System, Singapore, Singapore; 5 Department of Biochemistry, Yong Loo Lin School of Medicine, National University of Singapore, Singapore, Singapore; 6 Science Division, Yale-NUS College, Singapore, Singapore; 7 Cancer and Stem Cell Biology Program, Duke-NUS Medical School, Singapore, Singapore

**Keywords:** Predictive markers, Machine learning, Ageing

## Abstract

Clocks that measure biological age should predict all-cause mortality and give rise to actionable insights to promote healthy aging. Here we applied dimensionality reduction by principal component analysis to clinical data to generate a clinical aging clock (PCAge) identifying signatures (principal components) separating healthy and unhealthy aging trajectories. We found signatures of metabolic dysregulation, cardiac and renal dysfunction and inflammation that predict unsuccessful aging, and we demonstrate that these processes can be impacted using well-established drug interventions. Furthermore, we generated a streamlined aging clock (LinAge), based directly on PCAge, which maintains equivalent predictive power but relies on substantially fewer features. Finally, we demonstrate that our approach can be tailored to individual datasets, by re-training a custom clinical clock (CALinAge), for use in the Comprehensive Assessment of Long-term Effects of Reducing Intake of Energy (CALERIE) study of caloric restriction. Our analysis of CALERIE participants suggests that 2 years of mild caloric restriction significantly reduces biological age. Altogether, we demonstrate that this dimensionality reduction approach, through integrating different biological markers, can provide targets for preventative medicine and the promotion of healthy aging.

## Abstract (web summary)

Using a dimensionality reduction approach, Fong et al. generated a clinical aging clock (PCAge) that delineates healthy and unhealthy aging trajectories. They provide a streamlined version (LinAge) that maintains predictive power, and they demonstrate how the clock can be tailored to available data using the CALERIE study.

## Main

Although prevention is proverbially better than cure, current clinical recommendations promoting healthy aging focus on specific diseases and react to symptoms and signs of disease rather than focusing on organismal age^1^. Biological age (BA) is the most important risk factor determining individual risk of morbidity and mortality, with true BA of individuals generally different from chronological age (CA)^2^. Attempts to construct biological aging clocks, inferring BA from observable physical features (biomarkers), have a long history^2–4^. BA clocks have been constructed based on different classes of biological features, including clinical parameters^5–12^, DNA methylation (DNAm)^13–20^ and many types of -omics data^21–26^.

In addition to the underlying feature space, the operational definition of BA differs between approaches. Historically, BA is defined as the age at which the test subject’s physiology (as determined by its position in feature space) would be approximately normal for the reference cohort^27–29^. First-generation DNAm clocks follow this approach^13,16^. Although such clocks have attained impressive accuracy in determining CA, they are not optimized to predict future morbidity and mortality^18,30^.

Second-generation BA clocks aim to directly predict future mortality from biological parameters^17,18,31–33^. These clocks define true BA as ‘Gompertz age’, or the age commensurate with an individual’s future risk of dying from all intrinsic causes^18^. Second-generation clocks share some similarities with traditional clinical risk markers, such as the atherosclerosis cardiovascular disease (ASCVD) score^34^, but differ in that they predict all-cause mortality, better reflecting the high degree of interconnectivity between organ system and disease etiology^9,17,18,24,31–33^. Successful aging is more than the absence of specific diseases. Unlike existing clinical risk markers, BA clocks can identify individuals likely to remain free from age-dependent dysfunction, morbidity and mortality for years to come. BA clocks can, therefore, provide normative targets for clinical intervention and individual guidance to promote healthy aging.

Second-generation BA clocks require large-scale cohort data comprising data on biological features combined with long disease and mortality follow-up^35,36^. For standard clinical chemistry and physiological features, datasets meeting these criteria are available, enabling construction of second-generation ‘clinical clocks’ (CCs), designed to predict future mortality and morbidity directly from clinical features and biomarkers^15,17,18,31,37–39^. Unfortunately, equivalent historic data are not yet available for most -omics data, including DNAm. Current second-generation DNAm clocks have, therefore, been trained to either approximate BA predictions of existing CCs or approximate levels of the underlying biomarkers themselves^18–20,31^.

In settings where the relevant clinical features and blood markers are readily accessible, CCs have distinct advantages. The features on which CCs are built often have intrinsic well-established biological and pathophysiological meaning, making their findings comparatively easy to interpret and act upon clinically. The development and validation of more powerful CCs, as well as tools facilitating their clinical interpretation and application, should, therefore, be a priority. Extracting aging patterns from patient data can be challenging because many statistical and machine learning techniques require datasets with more examples (for example, subjects) than there are features^40^. Unfortunately, the cost and difficulty to generate such datasets scale with the number of samples and mortality follow-up time. This leaves the dimensionality of the feature space (the number of features collected for each subject) often being large compared to the number of subjects. One approach to address this challenge is dimensionality reduction, or transformation of data from a high-dimensional feature space into an approximately equivalent, lower-dimensional space^41^.

Principal component analysis (PCA) is a commonly used dimensionality reduction technique, based on singular value decomposition (SVD)^41^. SVD results in a transformation of the coordinate system of feature space into an equal number of ‘principal components’ (PCs). The transformation into PC space is a linear transformation (for example, rotation), mapping the original coordinate system of feature space to the new PC system, such that the coordinate axes align with directions in feature space along which the covariance (or correlation) between features is maximal across samples (subjects)^41^. PCA is widely used to extract insights from high-dimensional data. Because aging typically is the major source of variance in datasets derived from aging cohorts, this approach is especially appropriate for the extraction of aging patterns^42–44^. Importantly, because SVD/PCA is an analytical matrix factorization technique, it, unlike regression methods, feature selection and many nonlinear techniques, involves no model fitting, loss of data or algorithmic optimization. PCA is, therefore, not subject to hyper-parameter selection and can be applied to smaller datasets. PCA can be used to reduce dimensionality and compress data by omitting contributions from higher PCs (directions in feature space) that explain smaller amounts of the overall variance, although this approach may sometimes result in loss of useful information^45^. When constructing biomarkers of aging, morbidity and frailty, PCA can be employed as a feature selection/compression method and may increase the robustness of predictive models based on high-dimensional biomedical data^46–49^. The usefulness of this approach in the construction of BA clocks was previously explored by Nakamura et al.^5^. More recently, Higgins-Chen et al.^45^ demonstrated that DNAm clocks constructed from PC-transformed data exhibit increased reliability and reproducibility. In the present study, we explored the construction of second-generation CCs using large clinical datasets and dimensionality reduction by PCA.

## Results

### PCAge predicts biological age

We used a training dataset extracted from the National Health and Nutrition Examination Survey (NHANES) IV 1999–2000 cohort. The training cohort was initially composed of 1,476 males and 1,536 females aged 40–84 years with a feature set comprising data from medical examination, physiological and laboratory measurements. Using health-related questionnaire data, we also generated three derived indices (comorbidity index, self-health index and healthcare use index (see Methods for details on these derived scores)). The complete set of 165 clinical parameters included the derived scores; data from medical examination, morphology and body composition; and clinical laboratory and blood chemistry (Supplementary Table 1). Individuals with missing values in any of these parameters were removed, resulting in a final training dataset comprising 923 males and 852 females. We next converted parameters into z-scores before calculating the SVD for the training cohort. We then used PCA for linear dimensionality reduction, retaining only the first 18 singular vectors (PCs), accounting for 99% of the overall variance in the data (see Supplementary Fig. 1 for scree plot). Loadings of the 165 parameters for these 18 PCs were visualized using heatmaps (Supplementary Fig. 2), and these PCs, together with CA, were selected as covariates for Cox proportional hazard models predicting mortality separately for males and females (Supplementary Fig. 3). Hazard ratios were converted into BA as outlined below (Methods), yielding separate BA clocks for males and females (PCAge). We then tested PCAge in a testing cohort, comprising a separate set of subjects, extracted from the NHANES IV 2001–2002 recruitment wave. This cohort initially comprised 1,619 males and 1,631 females aged 40–84 years, with complete data available for 1,094 males and 942 females. The characteristics of the study participants are shown in Supplementary Table 2. Feature coordinates of subjects from the testing cohort were transformed into PC coordinates by projection using the right singular vectors of the training cohort before BA values were calculated.

**Fig. 1:**
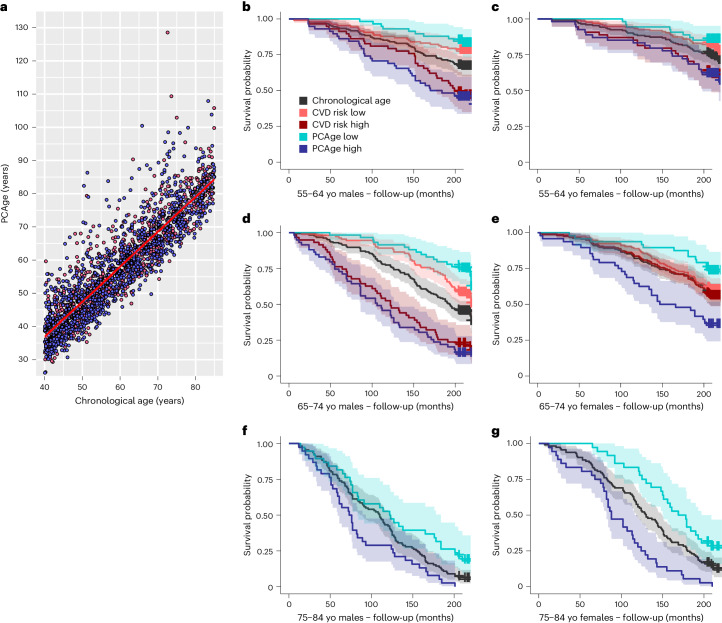
PCAge predicts BA in males and females. **a**, Scatter plot and linear regression of CA versus PCAge for males (blue) and females (red). PCAge is strongly correlated with CA for males (PCC = 0.88, R^2^ = 0.77, *P* < 0.001) and females (PCC = 0.90, R^2^ = 0.81, *P* < 0.001). **b**,**d**,**f**, Kaplan–Meier survival curves showing 20-year survival for males in different CA bins. Biologically younger males (best 25% quartile for BA, PCAge Low, cyan) and biologically older males (worst 25% quartile for BA, PCAge High, blue) are compared to males with BA similar to their CA (mean CA, black), males in the best 25% quartile for ASCVD risk score (CVD risk Low, orange) and males in the worst 25% quartile for ASCVD risk score per CA category (CVD risk High, red). Across all age categories, biologically younger males (PCAge Low, cyan) experienced significantly lower mortality compared to controls (*P* = 0.02 for 55–64, *P* < 0.001 for 65–74 and *P* = 0.03 for 75–84), whereas biologically older males (PCAge High, blue) experienced significantly higher mortality (*P* < 0.001 for 55–64 and 65–74 and *P* = 0.03 for 75–84). There were no statistically significant differences in the risk of dying between individuals with high ASCVD score and high PCAge. However, PCAge also captured subjects who were aging well, beyond just having low CVD risk. **c**,**e**,**g**, Kaplan–Meier survival curves over 20-year follow-up for females similar to **b**, **d** and **f**. When compared to mean CA (black), significant survival differences were observed in females (*P* = 0.05 for PCAge High (blue) in 55–64, *P* = 0.03 for PCAge Low (cyan) in 65–74, *P* = 0.003 for PCAge High (blue) in 65–74, *P* = 0.01 for PCAge Low (cyan) in 75–84 and *P* < 0.001 for PCAge High (blue) in 75–84), although this did not reach statistical significance for PCAge Low (cyan) in 55–64 (*P* = 0.06). In females, PCAge clearly outperforms the ASCVD score in predicting survival, specifically in those with CA 65–74 (*P* = 0.002 for PCAge High (blue) versus CVD risk High (red), although *P* = 0.09 for PCAge Low (cyan) versus CVD risk Low (orange)). Survival analyses were performed using log-rank tests. Areas shaded in color in **b**–**g** indicate 95% error bands for lines of the same color. yo, years old.

As expected, PCAge was highly correlated with CA in both males and females (Fig. 1a) but with significant residuals (PCAge Deltas). We next asked if the residuals between PCAge and CA encode information regarding individual aging trajectories—that is, if large negative or positive residuals were indicative of more or less successful aging. To address this question, we determined the correlation of PCAge, CA and their residuals with parameters of molecular aging (telomere length), cognitive performance (digit symbol substitution test) and physical function (gait speed). Although CA and PCAge were both significantly negatively correlated with telomere length (Supplementary Fig. 4a,d), cognitive performance (Supplementary Fig. 4b,e) and gait speed (Supplementary Fig. 4c,f), PCAge was more predictive of these parameters than CA alone (Supplementary Fig. 4). Subjects with negative PCAge residuals (biologically younger than their CA) had significantly longer telomeres (Supplementary Fig. 4g) and better preserved cognitive performance (Supplementary Fig. 4h), and they walked faster (Supplementary Fig. 4i), than expected based on their CA. By contrast, subjects with positive PCAge residuals (biologically older than their CA) had shorter telomeres (Supplementary Fig. 4g) and worse cognitive performance (Supplementary Fig. 4h), and they walked slower (Supplementary Fig. 4i), than expected for their CA. These data demonstrate that PCAge, despite originally being trained on survival only, is more predictive of molecular and physiological parameters expected to depend on BA than CA alone.

**Fig. 2:**
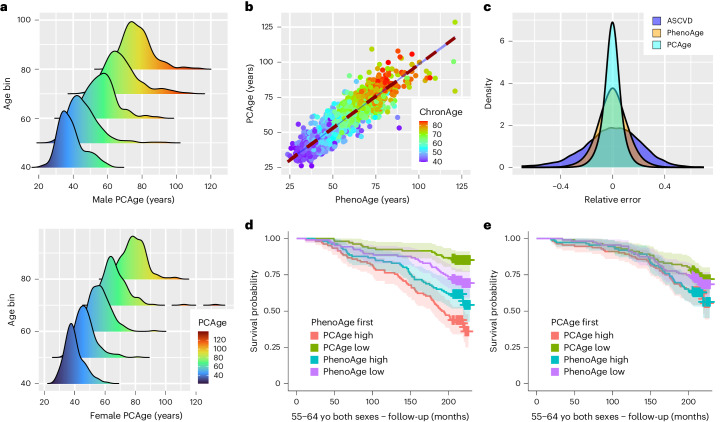
Testing PCAge for robustness and precision. **a**, Ridgeline plots of male and female populations binned by decade for CA. For each CA bin, PCAge for male and female populations possessed a long tail toward the right and contained distinct subpopulations of subjects who were significantly biologically older, especially in the 65–74-year and 75–84-year age bins. Subjects with positive PCAge Deltas of at least 20 years were significantly more likely to suffer from age-dependent diseases (median comorbidity index = 0.18, interquartile range (IQR) = 0.14–0.31 years, *n* = 41 males and 13 females versus the mean and s.d. of the median comorbidity index for age and sex-matched subjects within the normal distribution, which was 0.09 ± 0.02, *n* = 10,000 by bootstrapping, *P* < 0.001) and died significantly faster (median survival = 4.7 more years, IQR = 2.1–13.5 years, *n* = 41 males and 13 females versus the mean and s.d. of the median survival for age and sex-matched subjects who survived for 17.9 ± 0.4 more years, *n* = 10,000 by bootstrapping, *P* < 0.001). **b**, Scatter plot and linear regression of PhenoAge versus PCAge for males and females. The color gradient (ChronAge) reflects the CA of each subject. **c**, Impact of random errors in clinical parameters on BA clocks and disease score. For each clinical parameter, random errors were sampled from a Gaussian distribution with mean or 0% and s.d. 10%. The distribution for relative errors in the ASCVD score, PCAge and PhenoAge are compared. PCAge, using linear projections (PCAs) of many variables, is less impacted than models using only a small number of features. **d**, Kaplan–Meier survival curves over 20-year follow-up for subjects with PhenoAges of 55–64 years, stratified by PCAge or PhenoAge. PCAge High are subjects in the worst 25% quartile for PCAge; PCAge Low are subjects in the best 25% quartile for PCAge; PhenoAge High refers to subjects in the worst 25% quartile for PhenoAge; and PhenoAge Low refers to subjects in the best 25% quartile for PhenoAge. **e**, Equivalent to **d** but showing Kaplan–Meier survival curves for subjects with PCAges of 55–64 years. Survival analyses were performed using log-rank tests. Areas shaded in color in **d** and **e** indicate 95% error bands for lines of the same color. yo, years old.

**Extended Data Fig. 1:**
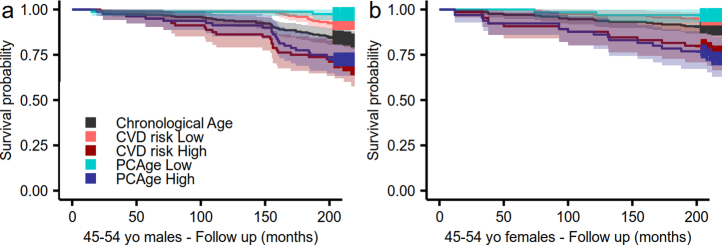
PCAge also predicts BA in chronologically 45–54 year old males and females. Kaplan-Meier survival curves over a 20-year follow up period for 45–54 year old males and females for mean CA (Chronological Age, black), biologically younger males/females in the best 25% quartile for BA per CA category (PCAge Low, cyan), biologically older males/females in the worst 25% quartile for BA per CA category (PCAge High, blue), biologically younger males/females in the best 25% quartile for ASCVD score per CA category (CVD risk Low, orange), and biologically older males/females in the worst 25% quartile for ASCVD score per CA category (CVD risk High, red). **a**, Compared to mean CA (black), male subjects in the best 25% quartile, with younger PCAges relative to their CAs (PCAge Low, cyan), had a shallower decline in survival (*P* = 0.002), whereas male subjects in the worst 25% quartile, with older PCAges relative to their CAs (PCAge High, blue), had a steeper decline in survival (*P* = 0.03). **b**, Compared to mean CA (black), female subjects in the worst 25% quartile, with older PCAges relative to their CAs (PCAge High, blue), had a steeper decline in survival (*P* = 0.001), although there was no statistically significant difference between mean CA (black) and female subjects in the best 25% quartile (PCAge Low, cyan) (*P* = 0.08). For both sexes, there were no statistically significant differences between the ASCVD score and PCAge in the ability to predict survival in the 45–54 age category. Survival analyses were performed using log-rank tests. Areas shaded in color in each panel indicate 95% error bands for lines of the same color.

We next tested the performance of PCAge in predicting survival in unknown subjects by selecting subjects in the test cohort within the best (lowest) 25% and worst (highest) 25% quartiles for BA (PCAge). Across all age categories, when compared to subjects with BA within 25% of their CA (mean CA), male subjects in the best 25% quartiles (PCAge Low) experienced significantly lower mortality over the 20-year follow-up, whereas male subjects in the worst 25% quartiles (PCAge High) experienced significantly higher mortality (Fig. 1b,d,f). Similarly, when compared to mean CA, significant survival differences were observed in females, although this did not reach statistical significance for PCAge Low in the 55–64-year age category (Fig. 1c,e,g). Several of the biological features used to calculate PCAge have known associations with clinical disorders and disease risk. To directly test the performance of PCAge in predicting survival relative to a known clinical risk marker, we compared its predictive power against the ASCVD score, a widely used metric to predict the 10-year risk of cardiovascular disease (CVD) or stroke^34^. Unlike the ASCVD score, we found that PCAge effectively predicts survival and mortality in both males and females aged 45–74 years (Fig. 1b–e and Extended Data Fig. 1). Subjects with large (positive) PCAge Deltas of at least 20 years were also significantly more likely to suffer from age-dependent diseases and died significantly faster (Fig. 2a).

### PCA increases robustness to random errors

To further explore the meaning of residuals between PCAge and CA, we next compared PCAge to a well-validated clinical BA clock, PhenoAge^18^. We found that PCAge and PhenoAge were highly correlated (Pearson correlation coefficient (PCC) = 0.91, R^2^ = 0.83, *P* < 0.001) (Fig. 2b). Despite strong correlation, there were significant residuals between both clocks (Fig. 2b and Supplementary Fig. 5). One explanation for these differences is sensitivity to random errors. Many clinical measurements are subject to measurement errors and significant day-to-day variations. For clinical parameters, typical variability has been estimated to be around 7–10% (https://www.westgard.cpm/biodatabase1.htm). We, therefore, compared the relative sensitivity of the ASCVD score, PhenoAge and PCAge to noise and found that the ASCVD score was impacted most significantly (Fig. 2c). By contrast, random errors largely average out across PCs, and, therefore, the relative error distribution for PCAge was the narrowest (Fig. 2c). The magnitude of the relative errors for PhenoAge was between that of the ASCVD score and PCAge. This may be because PhenoAge uses significantly fewer (nine) parameters compared to PCAge (165 parameters).

**Extended Data Fig. 2:**
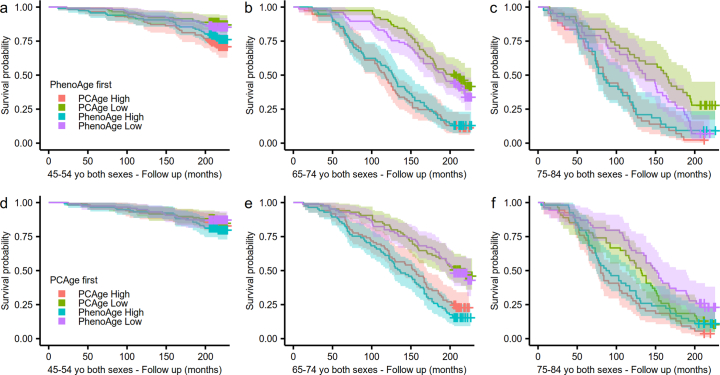
Kaplan-Meier survival curves by PhenoAge and PCAge categories for male and females. We compared PhenoAge’s and PCAge’s ability to stratify the test cohort by either first selecting individuals based on their PhenoAge before predicting survival based on their PCAge (a-c), or by first selecting based on PCAge before predicting survival based on PhenoAge (d-f). **a-c**, Across all PhenoAge categories, we found that PCAge could further predict survival within the PhenoAge selection, as evidenced by the statistically significantly wider degree of separation in the survival curves between the best 25% and worst 25% quartiles (*P* = 0.004 for PCAge Low (green) versus PCAge High (orange) and *P* = 0.1 for PhenoAge Low (purple) versus PhenoAge High (cyan) in the 45–54 PhenoAge category, *P* < 0.001 for PCAge Low (green) versus PCAge High (orange) and *P* < 0.001 for PhenoAge Low (purple) versus PhenoAge High (cyan) in the 65–74 PhenoAge category, *P* < 0.001 for PCAge Low (green) versus PCAge High (orange) and *P* = 0.05 for PhenoAge Low (purple) versus PhenoAge High (cyan) in the 75–84 PhenoAge category). **d-f**, However, when we evaluated the performance of PhenoAge in survival prediction in subjects selected according to their PCAge instead, we found significant differences only in the 65–74 (*P* < 0.001) and 75–84 (*P* = 0.004) PCAge categories. Our findings therefore suggest that PCAge in many cases could identify additional healthy aging and other at-risk individuals beyond that predicted by PhenoAge. Survival analyses were performed using log-rank tests. Areas shaded in color in a-f indicate 95% error bands for lines of the same color.

We next asked if these additional parameters enabled PCAge to capture meaningful biology and whether the residuals between PhenoAge and PCAge encoded additional biological information. To answer this question, we carried out a sequential sorting procedure, age-binning subjects not based on their CA but based on one of the clocks, before attempting to predict future survival using the other clock. We evaluated the ability of PCAge to predict future survival in subjects pre-selected (binned) according to their PhenoAge and vice versa. Across PhenoAge bins, we found that PCAge was able to further stratify survival in subjects binned by PhenoAge (Fig. 2d and Extended Data Fig. 2a–c), but the opposite was not true, with PhenoAge Deltas providing no further stratification in subjects binned by PCAge (Fig. 2e and Extended Data Fig. 2d–f). Our findings suggest that the additional parameters captured by PCAge enabled the identification of additional healthy aging and at-risk individuals, beyond those identified by PhenoAge.

### PCs map mechanisms of aging and age-related disease(s)

**Fig. 3:**
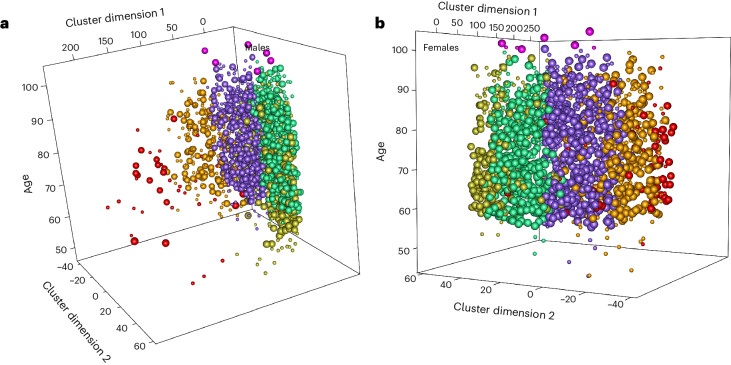
Cluster analysis. **a**, 3D plot of the five male clusters: ‘healthy aging’ (green), ‘mild cardio-metabolic’ (purple), ‘major cardio-metabolic’ (orange), ‘cardio-metabolic failure’ (red) and ‘multi-morbid’ (yellow). Centenarians are color-coded in pink. **b**, 3D plot of the five female clusters represented by the same colors as males in **a**. For both plots, the *z* axis shows CA at the end of the 20-year follow-up period (large spheres) or at death (small spheres). Subjects from the ‘healthy aging’ clusters had the lowest median PCAge and the smallest (most negative) PCAge Delta (*P* < 0.001). By contrast, subjects from the ‘cardio-metabolic failure’ clusters had the highest median PCAge and PCAge Delta (*P* < 0.001) (Supplementary Table 5). Across all clusters, there were six male and eight female centenarians. When we compared the centenarians to individuals of the same initial CA but who did not attain centenarian status, we found that centenarians had significantly lower mean PCAge Delta (−3.4 ± 4.7 years, *n* = 14 versus +1.0 ± 6.3 years, *n* = 259, *P* = 0.0056 by unpaired two-sided *t*-test), indicating that centenarians already had significantly lower BA at the time of the initial survey (that is, 15–20 years before turning 100).

Individual PCs comprise sets of correlated features (Supplementary Table 3). To explore the biological meaning of PC coordinates, we first selected the top nine PCs, based on their predictive value within PCAge (Supplementary Table 4 and Supplementary Fig. 3). We then combined training and testing cohorts, clustering all 2,017 male and 1,794 female subjects using *k*-means clustering based on their location along these nine PCs (Fig. 3). *k*-means clustering maximizes the separation between clusters^50^. The clustering algorithm assigns individuals to the same cluster who are similar to each other in the space spanned by the top nine PCs of PCAge. Members of the same cluster, therefore, share similarities in biological features that impact their future mortality. CA was not part of the data used by the clustering algorithm, and no significant differences in CA were detected between any of the male clusters (Supplementary Table 5) nor for most of the female clusters (Supplementary Table 5). To learn more about the subjects comprising each cluster, we next characterized clusters using demographic and clinical data (Supplementary Table 5). We identified three unique themes, including healthy aging (green clusters), a cardio-metabolic axis formed by three separate clusters (purple, orange and red clusters) and an additional ‘multi-morbidity’ group (yellow clusters).

**Fig. 4:**
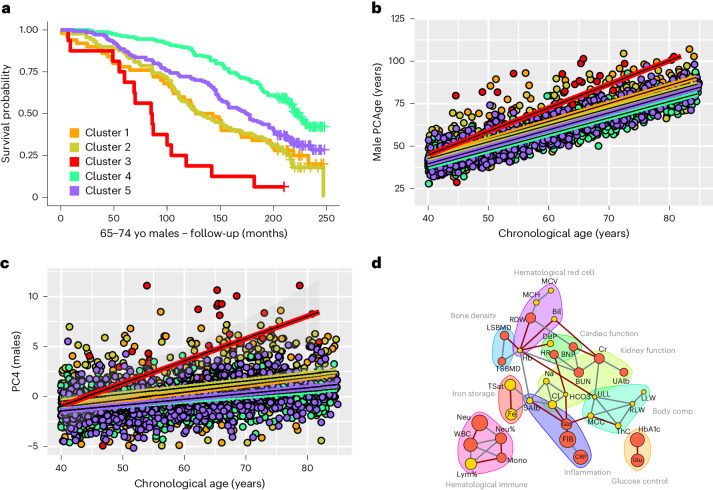
PCs to extract mechanisms of aging and age-related disease(s). **a**, Kaplan–Meier survival curves comparing cluster-specific survival for 65–74-year CA males. Males from the ‘healthy aging’ cluster (Cluster 4, green) had the lowest mortality, whereas males from the ‘cardio-metabolic failure’ cluster (Cluster 3, red) had the highest mortality. Males from the ‘multi-morbid’ clusters (Cluster 2, yellow), although significantly different from subjects of the ‘cardio-metabolic’ clusters (Cluster 5, purple, and Cluster 1, orange), experienced similar mortality to males from the ‘major cardio-metabolic’ cluster (Cluster 1, orange). Log-rank tests were statistically significant for all individual comparisons (*P* < 0.001), except between the ‘major cardio-metabolic’ and ‘multi-morbid’ clusters (*P* = 0.8) and between the ‘major cardio-metabolic’ and ‘mild cardio-metabolic’ (Cluster 5, purple) clusters (*P* = 0.1). **b**, Scatter plot and linear regression of CA versus PCAge for each male cluster. Cluster-specific aging rate is the slope of the linear fit between CA and PCAge. It is not the biological aging rate of individual subjects but characterizes the relationship between BA and CA across clusters. Males in the ‘healthy aging’ cluster (green) had the slowest cluster-specific aging rate, on average 1.03 years per calendar year (slope = 1.03, R^2^ = 0.87, *P* < 0.001). Males from the cardio-metabolic axis had increasing cluster-specific aging rates (slope = 1.07, R^2^ = 0.84, *P* < 0.001 for ‘mild cardio-metabolic’ (purple) and slope = 1.14, R^2^ = 0.70, *P* < 0.001 for ‘major cardio-metabolic’ (orange)), with the highest rate in the ‘cardiometabolic failure’ (red) (slope = 1.47, R^2^ = 0.61, *P* < 0.001) cluster. Males from the ‘multi-morbid’ cluster (yellow) had intermediate rate (slope = 1.06, R^2^ = 0.73, *P* < 0.001). **c**, Scatter plot and linear regression of CA versus PC4 for each male cluster. Although dispersion was high, PC4 increased with age in all clusters, including the ‘healthy aging’ (green) (slope = 0.047, R^2^ = 0.154, *P* < 0.001), ‘mild cardio-metabolic’ (purple) (slope = 0.058, R^2^ = 0.145, *P* < 0.001), ‘major cardio-metabolic’ (orange) (slope = 0.02, R^2^ = 0.023, *P* < 0.001), ‘multi-morbid’ (yellow) (slope = 0.068, R^2^ = 0.13, *P* < 0.001) and ‘cardio-metabolic failure’ (red) (slope = 0.24, R^2^ = 0.333, *P* < 0.001) clusters. **d**, Partial correlation network of clinical parameters within the top 10% by magnitude of weights in PC4. The circle size for each parameter is proportional to its weight within PC4. Parameters in red had a positive weight and increased with CA, whereas parameters in yellow had a negative weight and decreased with CA. yo, years old. MCV, mean cell volume; MCH, mean cell hemoglobin; RDW, red cell distribution width; Hb, hemoglobin; Bil, bilirubin; LSBMD, lumbar spine bone mineral density; TSBMD, thoracic spine bone mineral density; DBP, diastolic blood pressure; HR, heart rate; BNP, n-terminal pro-brain natriuretic peptide; Cr, creatinine; BUN, blood urea nitrogen; UAlb, urine albumin; Na, sodium; Cl, chloride; HCO3, bicarbonate; ULL, upper leg length; MCC, maximal calf circumference; ThC, thigh circumference; RLW, right leg weight; LLW, left leg weight; HbA1c, glycohemoglobin; Glu, glucose; SAlb, serum albumin; Glo, globulin; Fib, fibrinogen; CRP, c-reactive protein; TSat, transferrin saturation; Fe, iron; WBC, white blood cell count; Neu, neutrophil count; Neu%, neutrophil percent; Mono, monocyte count; Lym%, lymphocyte percent.

**Extended Data Fig. 3:**
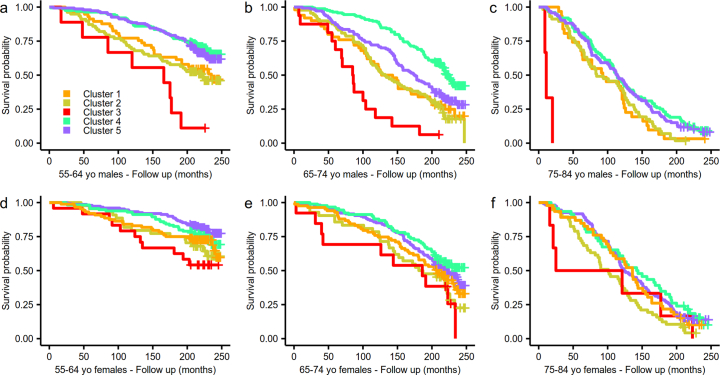
Kaplan-Meier survival curves by CA category for male and female clusters. **a**, Survival curves for male clusters in the 55–64 CA category. Log-rank tests were statistically significant for all individual curve comparisons, except between the ‘major cardio-metabolic’ (orange) and ‘multi-morbid’ (yellow) clusters (*P* = 0.6), and between the ‘healthy aging’ (green) and ‘mild cardio-metabolic’ (purple) clusters (*P* = 0.5). **b**, Refer to Fig. 4a. **c**, Survival curves for male clusters in the 75–84 CA category. Log-rank tests were statistically significant for all individual curve comparisons, except between the ‘major cardio-metabolic’ (orange) and ‘multi-morbid’ (yellow) clusters (*P* = 0.7), ‘major cardio-metabolic’ (orange) and ‘mild cardio-metabolic’ (purple) clusters (*P* = 0.05), and between the ‘healthy aging’ (green) and ‘mild cardio-metabolic’ (purple) clusters (*P* = 0.6). **d**, Survival curves for female clusters in the 55–64 CA category. Log-rank tests were statistically significant only for individual curve comparisons between the ‘mild cardio-metabolic’ (purple) and ‘multi-morbid’ (yellow) clusters (*P* = 0.02), ‘mild cardio-metabolic’ (purple) and ‘cardio-metabolic failure’ (red) clusters (*P* = 0.001), ‘mild cardio-metabolic’ (purple) and ‘major cardio-metabolic’ (orange) clusters (*P* = 0.04), and between the ‘healthy aging’ (green) and ‘cardio-metabolic failure’ (red) clusters (*P* = 0.02). **e**, Survival curves for female clusters in the 65–74 CA category. Log-rank tests were statistically significant only for individual curve comparisons between the ‘healthy aging’ (green) and ‘multi-morbid’ (yellow) clusters (*P* = 0.01), mild cardio-metabolic’ (purple) and ‘cardio-metabolic failure’ (red) clusters (*P* = 0.03), and between the ‘healthy aging’ (green) and ‘cardio-metabolic failure’ (red) clusters (*P* = 0.01). **f**, Survival curves for female clusters in the 75–84 CA category. Log-rank tests were statistically significant only for individual curve comparisons between the ‘mild cardio-metabolic’ (purple) and ‘multi-morbid’ (yellow) clusters (*P* = 0.008), and between the ‘healthy aging’ (green) and ‘multi-morbid’ (yellow) clusters (*P* = 0.006). Subjects from the ‘healthy aging’ clusters experienced the shallowest declines in survival across all CA categories, except for 55–64 year old females.

**Extended Data Fig. 4:**
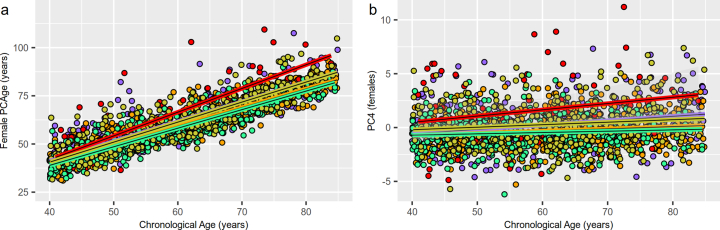
Cluster-specific aging rates and PC4 rates for females. **a**, Scatter plot and linear regression of CA versus PCAge for each of the five female clusters – ‘healthy aging’ (green), ‘mild cardio-metabolic’ (purple), ‘major cardio-metabolic’ (orange), ‘cardio-metabolic failure’ (red), and ‘multi-morbid’ (yellow). Females in the ‘healthy aging’ cluster (green) had the slowest cluster-specific aging rate, biologically aging on average 1.04 years per calendar year (slope=1.04, R^2^ = 0.86, *P* < 0.001 for females). Females from the cardio-metabolic axis had progressively faster cluster-specific aging rates (slope=1.04, R^2^ = 0.86, *P* < 0.001 for ‘mild cardio-metabolic’ (purple), and slope=1.12, R^2^ = 0.80, *P* < 0.001 for ‘major cardio-metabolic’ (orange)), with the highest cluster-specific aging rate seen in the ‘cardiometabolic failure’ (red) females (slope=1.31, R^2^ = 0.66, *P* < 0.001). Females from the ‘multi-morbid’ cluster (yellow) had intermediate cluster-specific aging rates (slope=1.10, R^2^ = 0.82, *P* < 0.001). **b**, Scatter plot and linear regression of CA versus PC4 for each of the five female clusters. Although dispersion was high, PC4 increased with age for the ‘healthy aging’ (green) (slope=0.012, R^2^ = 0.007, *P* = 0.038), ‘mild cardio-metabolic’ (purple) (slope=0.02, R^2^ = 0.023, *P* < 0.001), ‘major cardio-metabolic’ (orange) (slope=0.023, R^2^ = 0.013, *P* = 0.02), and ‘multi-morbid’ (yellow) (slope=0.04, R^2^ = 0.058, *P* < 0.001) clusters. There was no statistically significant increase with age in the already high PC4 values in the ‘cardio-metabolic failure’ (red) (slope=0.04, R^2^ = 0.058, *P* = 0.19) cluster.

Across sex and CA bins, subjects from the ‘healthy aging’ (green) clusters were biologically significantly younger, had a slower cluster-specific aging rate and had significantly higher survival over the 20-year follow-up period compared to other clusters (Figs. 3 and 4a,b, Extended Data Figs. 3 and 4 and Supplementary Table 5). Subjects from the cardio-metabolic axis, comprising a spectrum across the ‘mild cardio-metabolic’ (purple) to the ‘major cardio-metabolic’ (orange) and ‘cardio-metabolic failure’ (red) clusters, exhibited increasingly positive PCAge Delta, progressive decline in survival and overall faster cluster-specific aging rates, with a majority suffering and dying from CVD (Figs. 3 and 4a,b, Extended Data Figs. 3 and 4 and Supplementary Table 5). Along this cardio-metabolic axis, subjects became increasingly obese, sedentary and frail (Supplementary Table 5). Members of the ‘multi-morbid’ (yellow) cluster also failed to age successfully and formed a distinct group of subjects, outside the cardio-metabolic axis, with median PCAge Deltas significantly higher than ‘healthy agers’ and ‘mild cardio-metabolic’ clusters but lower than the ‘major cardio-metabolic’ and ‘cardio-metabolic failure’ clusters (Supplementary Table 5). Although males and females from the ‘multi-morbid’ clusters differed in terms of education and socioeconomic factors (Supplementary Table 5), members of both sexes had significantly more current smokers and subjects with alcohol use disorder, and their members had the lowest body mass index (BMI) among all the clusters (Supplementary Table 5). When we compared centenarians across all clusters, we found that centenarians had significantly lower mean PCAge Delta than matched controls (Fig. 3). For females, there were statistically significantly more centenarians in the ‘healthy aging’ cluster than expected based on the dataset as a whole (*P* = 0.03) (Supplementary Table 5). Interestingly, ‘healthy agers’ used the healthcare system more proactively and effectively than members of other clusters (Supplementary Table 5 and Supplementary Fig. 6), suggesting that early and proactive treatment of risk factors and age-related disease(s) are determinants of more successful aging trajectories. Many members of the ‘multi-morbid’ clusters suffered from, and died of, a variety of chronic, non-cardiovascular diseases (Supplementary Table 5). When treatment was indicated, there were significantly fewer members from the ‘multi-morbid’ clusters who received the required chronic medications at an earlier age, and significantly more relatively younger members who required treatment were missed (Supplementary Table 5). In general, male members of the ‘multi-morbid’ cluster accessed healthcare less frequently, and those who did relied more on emergency treatment (Supplementary Table 5). Taken together, these results suggest that lack of early, preventative and proactive treatment of age-related disease(s) and associated risk factors contributes to unsuccessful aging later in life (see Supplementary Note 1 in the Supplementary Information for detailed cluster analysis).

The cluster analysis shows that individuals separated in feature space along the major PCs selected by PCAge differ not only by life expectancy but also by socioeconomic, lifestyle and behavioral factors, even though none of these factors was originally included in the model. We next asked if membership of the ‘healthy aging’ cluster was associated with specific PC coordinates and if this could be exploited to extract pathways of healthy aging and inform intervention strategies aimed at moving subjects into the ‘healthy aging’ cluster. We found that PC2, followed by PC4, resulted in the greatest separations between the ‘healthy aging’ cluster and other clusters (Supplementary Table 4). When we sorted the clinical measures within PC2 by absolute magnitude and direction of their weights, we found that the clinical measures with highly weighted coefficients were mainly body composition and fat (Supplementary Table 6). In terms of interventions, the implications were obvious and expected, suggesting that improved exercise and diet would impact PC2 and result in more successful aging.

When we applied the same approach to PC4, we found that PC4 was substantially lower in the ‘healthy aging’ cluster (Supplementary Table 4) compared to all other clusters. Moreover, PC4 was significantly positively correlated with CA in all but one cluster (female ‘cardio-metabolic failure’) (Fig. 4c and Extended Data Fig. 4b). It is noteworthy that, despite the differences in body composition and disease spectrum that separate these two groups, high PC4 values were associated with less successful aging in both the ‘cardio-metabolic’ and ‘multi-morbid’ clusters, suggesting that PC4 captures a common feature of all unsuccessful aging. To identify underlying mechanisms, we built a partial correlation network including only the top 10% (by PC4 absolute weight) of clinical parameters (Fig. 4d). We categorized these measures into biomedical categories related to body composition, physiological functions and responses, finding that PC4 encodes important information on pathways relating to cardiac function, renal function, inflammation and immunity, glucose regulation and iron storage and erythropoiesis (Fig. 4d). Elevated values in PC4 appear to capture abnormal clinical measures, thereby reflecting dysregulation in these pathways. Interestingly, the PC4 network gives substantial weight to markers of inflammation, a process known to play a central role in many age-dependent diseases.

### Angiotensin-converting enzyme inhibitors/angiotensin receptor blockers normalize PC4 to reduce mortality risk and BA

**Fig. 5:**
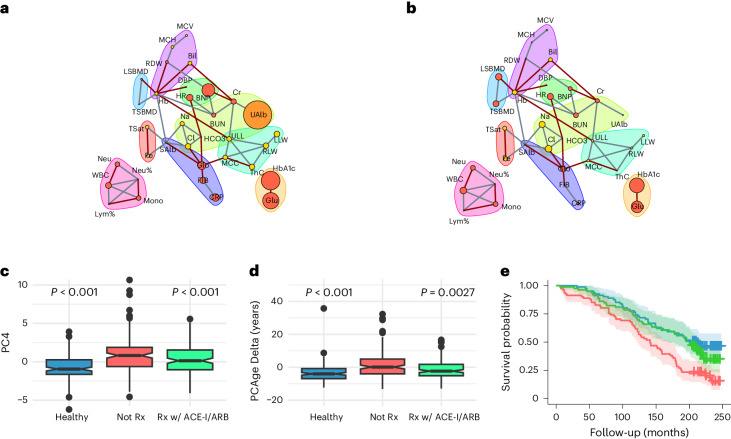
ACE-I/ARBs normalize modifiable clinical parameters, involved in renal function, cardiac function and inflammation, within PC4 space to reduce mortality risk and BA. **a**, Comparison of PC4 networks of subjects with high urine ACR and not on treatment, superimposed on reference healthy subjects with normal urine ACR and without hypertension, hyperlipidemia or diabetes mellitus. **b**, Comparison of PC4 networks of ACE-I/ARB-treated subjects superimposed on reference healthy subjects. Parameters in red had a positive weight in PC4, increasing with CA, whereas parameters in yellow had a negative weight and decreased with CA. During the comparison, parameters that became worse were scaled by the log_2_ fold change relative to healthy subjects. Urine albumin (UAlb) is colored orange because it was used as the original selection criterion. Refer to Fig. 4 for a list of abbreviations. **c**, Notched box plots of PC4 weights for healthy subjects (blue), untreated subjects with high urine ACR (red) and ACE-I/ARB-treated subjects (green) (*n* = 140 per group). Notch of box blots indicates median value. Lower and upper hinges correspond to 25th and 75th percentiles, respectively. Whiskers extend to ±1.5 multiplied by interquartile range, with points outside this range drawn individually. Multiple group comparisons were performed using the Kruskal–Wallis test. Post hoc analyses were performed using Dunn’s test. **d**, Notched box plots of PCAge Delta for the same groups (*n* = 140 per group), constructed and analyzed as in **c**. **e**, Kaplan–Meier survival curves for the same groups in **c**. Survival analyses were performed using log-rank tests. Areas shaded in color indicate 95% error bands for respective lines. Rx, treatment.

Microalbuminuria, which is often secondary to chronic hypertension and/or longstanding diabetes mellitus, is an early manifestation of chronic kidney disease, associated with increased cardiovascular risk^51^. Microalbuminuria is considered clinically significant when the urine albumin-to-creatinine ratio (ACR) is ≥30 mg g^−1^ (ref. ^51^). We first matched (1) healthy subjects with normal urine ACR and without hypertension, hyperlipidemia or diabetes mellitus; (2) subjects with high urine ACR and not on treatment; and (3) subjects treated with angiotensin-converting enzyme inhibitors (ACE-Is) or angiotensin receptor blockers (ARBs) who had normal urine ACR (successfully treated). Subjects were matched by CA, sex, smoking status (serum cotinine) and BMI (*n* = 140 per group). We then compared the PC4 network of untreated subjects with high urine ACR against healthy subjects (Fig. 5a). In untreated subjects with high urine ACR, we found statistically significant increases in urine albumin (*P* < 0.001), N-terminal pro-brain natriuretic peptide (NT-proBNP) (*P* = 0.0074), globulin (*P* < 0.001), C-reactive protein (CRP) (*P* = 0.047), glycohemoglobin (HbA1c) (*P* < 0.001) and glucose (*P* < 0.001). Our results show that untreated subjects with high urine ACR also had dysregulated pathways involving renal and cardiac function, inflammation and glucose regulation. These findings are expected and consistent with known associations and outcomes of albuminuria, but we also found increased inflammation for untreated subjects with high urine ACR. Compared to healthy subjects, untreated subjects with high urine ACR had statistically significantly higher median PC4 value (*P* < 0.001) (Fig. 5c), higher positive median PCAge Delta (*P* < 0.001) (Fig. 5d) and higher mortality (*P* < 0.001) (Fig. 5e).

Given these findings, treatments to normalize urine ACR using best clinical practice, such as an ACE-I or ARB^51,52^, might be expected to normalize PC4 values and lower PCAge. Apart from their reno-protective effects, ACE-I/ARBs have additional effects of lowering blood pressure and are cardio-protective, preventing heart failure^53^. When we compared the PC4 network of ACE-I/ARB-treated subjects against healthy subjects, we found that there were no longer any significant differences in urine albumin, serum creatinine and NT-proBNP (Fig. 5b). Surprisingly, successful treatment with ACE-I/ARBs was also associated with lower CRP (Fig. 5b), which suggests that treatment with ACE-I/ARBs resulted in additional anti-inflammatory effects, either directly or through effects on general systemic function. ACE-I/ARB-treated subjects had statistically significantly lower median PCAge Delta (*P* = 0.0027), resulting in an overall negative (PCAge lower than CA) PCAge Delta (Fig. 5d). Consistent with both the disease-specific benefits of ACE-I/ARBs and the normalization of PCAge Deltas, treated subjects had better survival over the 20-year follow-up period (*P* = 0.003), with no remaining statistically significant differences in survival between ACE-I/ARB-treated and healthy subjects (Fig. 5e).

When we compared ACE-I/ARB-treated to untreated subjects with high urine ACR, we not only found lower urine albumin (*P* < 0.001) and NT-proBNP (*P* = 0.047) levels, as expected, but also statistically significantly lower levels of inflammatory markers, including serum globulin (*P* = 0.011), CRP (*P* = 0.047), fibrinogen (*P* = 0.025), ferritin (*P* = 0.03) and lactate dehydrogenase (LDH) (*P* < 0.001) in ACE-I/ARB-treated subjects. Taken together, our data suggest that treatment of microalbuminuria with ACE-Is/ARBs reduces mortality risk and BA by normalizing modifiable clinical parameters, involved in renal function, cardiac function and inflammation, moving subjects along the PC4 axis in feature space.

### The reduced clinical clock (LinAge) recapitulates PCAge

It is impractical to measure all 165 parameters included in PCAge. We, therefore, developed a reduced BA clock derived from PCAge but using a minimal set of parameters (LinAge). Using sensitivity analysis, we selected a subset of clinical parameters for inclusion to retain the predictive power of PCAge. LinAge includes only parameters from the complete blood count, renal function tests, liver function tests, iron panel and lipid panel in addition to vitamin B12, folate, CRP, fibrinogen, LDH, NT-proBNP, uric acid, glucose, HbA1c, urine ACR, blood pressure, pulse rate, BMI, smoking status and medical history (Supplementary Tables 1 and 7). All parameters used in LinAge can be measured in most standard clinical laboratories (see Methods, Code and Supplementary Files in the Supplementary Information for more details on ‘custom clocks’).

**Fig. 6:**
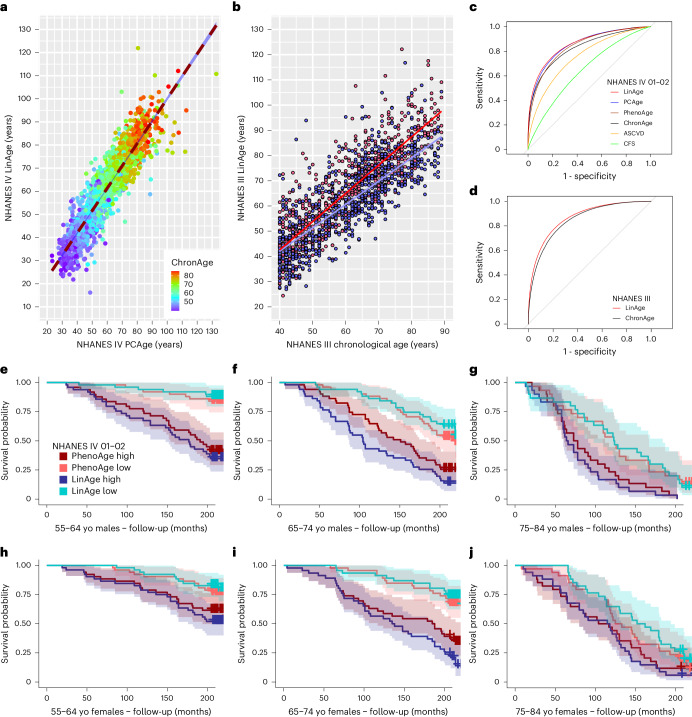
LinAge recapitulates PCAge in BA prediction. **a**, Scatter plot and linear regression of LinAge versus PCAge for both sexes in the NHANES IV test cohort. The color gradient (ChronAge) reflects CA. LinAge is strongly correlated with PCAge (PCC = 0.92, R^2^ = 0.84, *P* < 0.001, *n* = 2,036). PCAge Deltas were correlated with LinAge Deltas (PCC = 0.68). **b**, Scatter plot and linear regression of CA versus LinAge for males (blue) and females (red) in the NHANES III external validation cohort. LinAge is highly correlated with CA for males (blue symbols, PCC = 0.79, R^2^ = 0.62, *P* < 0.001, *n* = 715) and females (red symbols, PCC = 0.87, R^2^ = 0.76, *P* < 0.001, *n* = 819). **c**, ROC curves for 20-year all-cause mortality for LinAge, PCAge, PhenoAge, ChronAge, ASCVD and CFS scores in the test cohort. There was no significant difference in AUCs between PCAge (AUC = 0.8643) and LinAge (AUC = 0.8655). PCAge was significantly more informative than the ASCVD score (AUC = 0.7594, *P* < 0.001) in predicting future mortality. Compared to LinAge, ChronAge (AUC = 0.8289, *P* < 0.001), the CFS score (AUC = 0.6585, *P* < 0.001) and PhenoAge (AUC = 0.8474, *P* < 0.001) were significantly less predictive of 20-year survival. **d**, Similarly, in the NHANES III cohort, LinAge (AUC = 0.8741) predicted future all-cause mortality at 25-year follow-up significantly better than ChronAge (AUC = 0.8590, *P* = 0.03). ROC curves were compared using DeLong’s test. **e**–**j**, Kaplan–Meier survival curves of male or female subjects from the test cohort over 20-year follow-up. Subjects from each CA bin were stratified, selecting the highest (worst) and lowest (best) 25% difference between their CA and either LinAge or PhenoAge. In each age bin, subjects in the lowest (best) quartile of LinAge Delta (LinAge Low) were compared with the equivalent group of PhenoAge Delta (PhenoAge Low). Similarly, subjects in the highest (worst) quartile of LinAge Delta (LinAge High) were compared with the equivalent group of PhenoAge Delta (PhenoAge High). Across all age and sex categories, LinAge and PhenoAge captured subjects who aged unusually well (LinAge/PhenoAge Low) or badly (LinAge/PhenoAge High). Although the separation between survival curves for the best 25% and worst 25% quartiles of LinAge was wider than for PhenoAge in most age bins, the two clocks performed similarly in others. Areas shaded in color in **e**–**j** indicate 95% error bands for lines of the same color. yo, years old.

**Extended Data Fig. 5:**
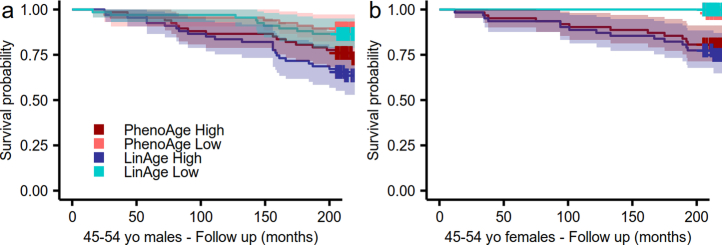
LinAge and PhenoAge in chronologically 45–54 year old males and females. **a-b**, Kaplan-Meier survival curves showing actual survival in the NHANES 2001–2002 test cohort over a 20-year follow up period for both sexes comparing biologically younger subjects in the bottom 25% quartile for LinAge Delta (LinAge Low, cyan) and PhenoAge Delta (PhenoAge Low, orange) with biologically older subjects in the upper 25% quartile for LinAge Delta (LinAge High, blue) and PhenoAge Delta (PhenoAge High, red) in the 45–54 CA category. Mortality is overall low in this CA bin and both clocks perform similarly with no statistically significant differences between LinAge Low (cyan) and PhenoAge Low (orange), as well as between LinAge High (blue) and PhenoAge High (red), in either sex. Areas shaded in color in each panel indicate 95% error bands for lines of the same color.

By design, LinAge is highly correlated with PCAge (Fig. 6a). To directly compare the performance of LinAge to PCAge in predicting survival, we first compared LinAge and PCAge predicted 20-year survival for all subjects in our test cohort. We also compared both clocks against CA, the ASCVD score and a widely used and well-validated measure of frailty in the clinic: the Clinical Frailty Scale (CFS)^54^ (Fig. 6c). Both LinAge and PCAge outperformed CA, the ASCVD score and CFS, with no significant difference in the areas under the curve (AUCs) between PCAge and LinAge (Fig. 6c), suggesting that, despite the reduction in the number of parameters, PCAge and LinAge performed similarly in the NHANES IV test cohort. We also compared LinAge directly to PhenoAge using receiver operating characteristic (ROC) analysis (Fig. 6c) and in individual age bins (Fig. 6e–j and Extended Data Fig. 5). LinAge has a statistically significantly larger AUC when predicting future survival in the NHANES IV test cohort across all ages (Fig. 6c). LinAge also outperformed PhenoAge in some individual bins, although they performed similarly in other age and sex bins, especially in very old individuals (Fig. 6e–j and Extended Data Fig. 5). Finally, we compared LinAge’s and PhenoAge’s ability to predict specific causes of death, finding that LinAge overall outperformed PhenoAge in predicting 20-year CVD and non-CVD-related and cancer-related mortality. This advantage was more pronounced for non-CVD deaths (Supplementary Fig. 7b–d).

PCs can be strongly affected by outliers, thresholding and batch effects^41^. When extracting LinAge parameters, we collapsed both the projection into the PC coordinate system of the training dataset and the linear risk model into a single set of parameters. LinAge was trained in the NHANES IV 1999–2000 recruitment wave and performed well when tested in the separate NHANES IV 2001–2002 wave (Fig. 6a). Although none of the subjects of the training cohort was part of the testing cohort, both cohorts were recruited as part of NHANES IV. It could be argued that comparison between different waves does not adequately test the potential impact of batch effects and methodological differences that would be expected when applying LinAge to subjects from an independent trial. To address this concern, we used data from NHANES III, which was a study preceding NHANES IV that ran from 1988 to 1994. Both studies differed in key experimental aspects (Supplementary Table 8). Because NHANES III was initiated a decade earlier, linked mortality data for NHANES III are also substantially longer than for NHANES IV. Despite these differences, when applied to NHANES III data, LinAge could successfully stratify survival in NHANES III subjects without batch or thresholding corrections for most parameters and without the need for re-training (Fig. 6b and Supplementary Fig. 8). Although LinAge was only trained with up to 20-year follow-up, it performed equally well in predicting survival when applied to the longer follow-up time (25 years) in NHANES III (Fig. 6d).

### Caloric-restricted subjects have lower aging rates

One important application for aging clocks is to evaluate the impact of intervention strategies on BA. The CALERIE phase 2 randomized controlled trial was designed to test the effects of moderate (25%) calorie restriction (CR). A cohort of 220 healthy non-obese volunteers between the ages of 20 years and 50 years were randomly assigned to either CR or ad libitum (AL) control groups and followed over 2 years^55^. Although, in practice, subjects from the CR group achieved only relatively moderate CR (12%), this nevertheless resulted in a significant reduction in several known CVD risk factors^56^, with reduction in BA estimates based on three different algorithms (Klemera–Doubal BA, PhenoAge and homeostatic dysregulation)^28,57^. However, a post hoc analysis using DNAm clocks found that only one DNAm clock (DunedinPACE) was able to identify significant effects, with no changes reported by several other DNAm clocks, including PhenoAge and GrimAge^58^. Unfortunately, the parameters reported for CALERIE do not overlap sufficiently with LinAge to apply it directly. However, the PCA approach can be used to re-train custom clocks based on different subsets of the feature space.

**Fig. 7:**
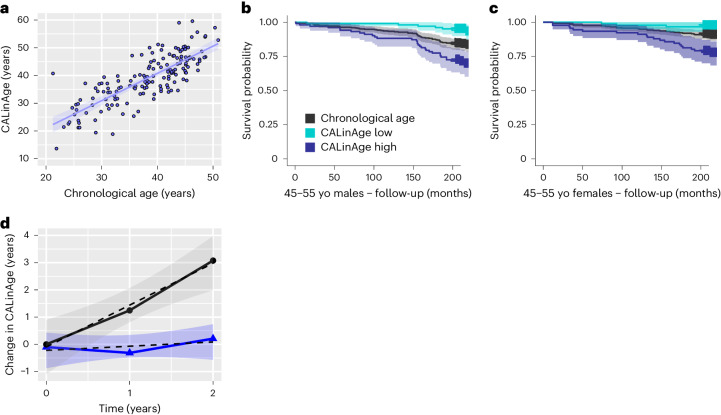
Caloric-restricted subjects have significantly lower aging rates. A custom PC-based clock was created to measure BA of CALERIE trial subjects. This clock was built exclusively on features found in data from both the NHANES IV 1999–2002 cohorts and the CALERIE trial. The CALinAge clock was trained in the NHANES IV 1999–2000 cohort, tested in the NHANES IV 2001–2002 cohort and then applied to CALERIE trial subjects. Comparison of CALinAge residuals with LinAge residuals in the NHANES IV 2001–2002 testing cohort confirmed a high degree of correlation between these two clocks (PCC = 0.70, *n* = 3,391). **a**, Scatter plot and linear regression of CA versus CALinAge for both sexes before the start of the CALERIE trial. CALinAge is highly correlated with CA (PCC = 0.80, R^2^ = 0.64, *P* < 0.001, *n* = 159). **b**,**c**, Kaplan**–**Meier survival curves showing actual survival in the NHANES IV 2001–2002 test cohort over the 20-year follow-up period for both sexes in the 45–55-year CA category. Compared to subjects with BA similar to their CA, subjects in the best 25% quartiles for BA (CALinAge Low) experienced significantly lower mortality over the 20-year follow-up (*P* = 0.01 for males and *P* = 0.05 for females), whereas subjects in the worst 25% quartiles for BA (CALinAge high) experienced significantly higher mortality (*P* = 0.003 for males and *P* = 0.001 for females). Survival analyses were performed using log-rank tests. Areas shaded in color in **b** and **c** indicate 95% error bands for lines of the same color. **d**, Change in CALinAge BA from baseline (0 years) to 1-year and 2-year follow-ups in the AL (black) and CR (blue) groups of the CALERIE trial. The points represent the mean values of change between the timepoint and baseline for each group. The shaded areas show the 95% confidence interval for the overall linear fit of the change in CALinAge as a function of treatment time for each group. Areas shaded in gray indicate 95% error bands for best linear fit for each condition. yo, years old.

To test this approach, we determined the set of parameters reported for both NHANES IV and CALERIE (Supplementary Table 1) and then applied the same procedure outlined for PCAge/LinAge (see Code and Supplementary Files in the Supplementary Information) to train and validate a mortality clock in NHANES IV (Fig. 7a). We next confirmed that the resulting ‘CALinAge’ custom clock could predict mortality differences in the NHANES IV test cohort within the age range relevant for CALERIE (Fig. 7b,c). CALinAge (AUC = 0.8282) has a statistically significantly larger AUC than CA (AUC = 0.7910, *P* < 0.001) when predicting future survival in the NHANES IV test cohort. We then applied CALinAge to CR and AL subjects, comparing the change in BA from baseline between CR and AL (Fig. 7d), adopting a similar approach as reported previously^57,58^. Using this approach, we calculated a CALinAge aging rate of 1.54 years per calendar year over the course of two calendar years for the AL group (95% confidence interval: 0.46–2.61, *n* = 55) and a significantly (*P* = 0.0022, comparison of linear models by two-way ANOVA) lower aging rate of 0.11 years per calendar year for the CR group (95% confidence interval: –0.58 to 0.79, *n* = 97) (Fig. 7d). Despite high inter-individual variability, the 95% confidence interval of the aging rate for CR subjects includes zero, indicating that the average aging rate in this group was insignificantly different from zero. These data suggest that CR, under the conditions realized in CALERIE, was able to significantly reduce biological aging as evaluated by CALinAge.

## Discussion

In this study, we constructed CCs using dimensionality reduction by PCA to generate BA estimates from clinical parameters. We developed and validated a CC (PCAge) that estimates BA using linear dimensionality reduction in a large clinical feature space, followed by Cox proportional hazards regression against mortality. Based on data from a single survey timepoint, often decades before death, PCAge showed significant predictive efficacy over 20 years and across a wide range of ages, illustrating the power of CCs in characterizing individual future aging trajectories, well before the onset of any specific pathology.

An advantage of CCs is that they are constructed from parameters that can be linked directly to the pathophysiology of specific diseases, making it easier to translate CC residuals into useful insights. Although PCA can facilitate both extraction and interpretation of feature space trajectories associated with organismal aging^5,42–44,59^, working in PCA/SVD coordinates comes with a tradeoff in terms of abstraction. This is because PCA coordinates are linear combinations of the original features and can be more difficult to interpret^41,60^. Nevertheless, individual aging PCs capture sets of features that exhibit correlated change during aging. By mapping to specific themes or aging processes captured by the data, analysis of individual PCs may, therefore, aid interpretation of feature space trajectories. Care must be taken when interpreting PCs in this way because they can be sensitive to outliers and thresholding effects and are generally not efficient in isolating distinct pathways. This tension between interpretability and efficacy is common to many machine learning techniques, and there are approaches to overcome it^25,61^.

Here we show that analysis of aging PCs can aid interpretation, leading to the identification of mechanisms of age-dependent failure and of potential intervention against them. By clustering subjects based on their location in the lower-dimensional space spanned by those PCs with significant weights in PCAge, we found that cluster membership was systematically associated with how successfully subjects aged. A ‘healthy aging’ cluster comprised subjects who were biologically younger and aged more successfully. Parameter values defining subjects from this cluster can be interpreted as normative values, defining features of healthy physiology at all ages. Overall distance from this cluster was associated with less successful aging. We found that, although there are different ways to age unsuccessfully, these are all associated with moving away from the main ‘healthy aging’ cluster along different directions in PC space. This suggests that healthy agers are all alike, but unhealthy subjects are unhealthy in their own way.

Although our analysis demonstrates that PCA is a useful technique for dimensionality reduction and for the identification of patterns in high-dimensional aging datasets, it is, of course, far from the only such approach. For example, nonlinear generalizations of PCA, such as variational autoencoders^43^, can be used to identify more complex patterns from high-dimensional data and may provide superior models, although this increased power comes at the cost of making interpretation more challenging.

Unfortunately, cross-sectional datasets, such as NHANES, do not allow comparison of subjects before and after interventions. This limits our ability to interpret the causality of the observation that ACE-Is/ARBs impact aging parameters. Furthermore, PCAge and LinAge include several parameters that are risk markers for age-dependent pathologies. Specifically, both include all the parameters found in the ASCVD score. However, BA clocks and clinical risk markers differ, both in goal and in approach. Clinical risk scores, by design, are hypothesis driven and organ/disease specific and aim to predict and detect specific pathologies or proximity to specific disease attractors. By contrast, CCs are data driven and disease agnostic, aiming to extract predictors of all-cause mortality from a collection of biological parameters, essentially quantifying the degree of an individual’s deviation from an optimal heathy aging trajectory. PCAge and LinAge are sensitive to a more complete set of mortality causes, and, unsurprisingly, they generally outperform the ASCVD score in predicting overall future mortality. This advantage is most obvious for individuals who are aging unusually well (whose BA is lower than their CA), because low cardiovascular risk alone does not guarantee healthy aging, but healthy aging is incompatible with substantially elevated cardiovascular risk.

The geroscience approach aims to practice preventative medicine by understanding and intervening in fundamental processes of aging or modulating the biological process(es) that drive the shift from healthy functioning toward systemic aging and the eventual manifestations of age-related disease(s). Geroscience shares many of the goals of traditional preventative medicine but seeks to push the boundaries of prevention to earlier ages, long before disease or overt abnormalities are detectable. Interpretable second-generation CCs can aid this goal. By analyzing factors related to elevated BA, we may be able to identify mechanisms separating successful from less successful aging. For example, subjects in the ‘healthy aging’ cluster had significantly lower values along PC4. Analysis of parameter weights for PC4 revealed that it encoded information on disease pathways relating to systemic inflammation and immunity, impaired cardiac and renal function, glucose regulation, iron storage and erythropoiesis. This observation is consistent with the importance of organism-level aging patterns and the role of chronic sterile inflammation as a key driver of age-dependent decline, morbidity and mortality^25^.

When interpreting CALERIE outcomes with reference to a clock trained in NHANES IV, we faced a common issue—that is, two datasets that have some, but not all, features in common. To address this challenge, we used dimensionality reduction by PCA to train a custom clock based only on features present in both datasets (see Code and Supplementary Files in the Supplementary Information for an example dataset and more detailed explanation on generating custom PC clocks). CCs can aid rapid intervention testing as our analysis of CALERIE suggests, showing that mild CR significantly reduces biological aging.

Finally, aging clocks are not replacements for disease-specific risk markers or differential diagnosis. They differentiate subjects who are aging well from those who are aging poorly, helping us to define the former and pointing to interventions to help the latter. Early and proactive modification of known risk factors, using primary disease prevention approaches as well as existing pharmacological interventions, can play an important role in maintaining subjects on optimal aging trajectories, delaying manifestations of aging, including age-related disease, and, in turn, extending healthy lifespan. A key goal of geroscience is to intervene proactively at a time when interventions are most efficacious, years or decades before any overt pathology is present. In this sense, BA clocks are to geroscience what clinical risk scores are to traditional primary prevention. Mature BA clocks will allow healthcare providers and governments to navigate the complexities of the risk–benefit analysis essential for adding years to healthy lifespan.

## Methods

### NHANES IV study design and participants

The continuous NHANES IV is an ongoing cohort study, by the National Center for Health Statistics, designed to assess the health and nutritional status of a nationally representative population of adults in the United States^35^. The study involves a series of cross-sectional surveys that includes demographic, socioeconomic, dietary information; responses to health-related questions; medical and physiological measurements; and results of laboratory tests. NHANES IV is approved by the National Center for Health Statistics Research Ethics Review Board. All study participants are de-identified, and data from NHANES IV are publicly available^35^. In the present study, we included adults aged 40–84 years, recruited for the 1999–2000 and 2001–2002 cohorts. Linked mortality data were obtained from the National Death Index^62^ and are available from 1 January 1999 until 31 December 2019.

The entire NHANES 1999–2002 dataset was initially composed of 5,700 participants and 186 clinical parameters, which included data from health-related questions and physiological and laboratory measurements. Using the health-related questions, we generated three derived indices, including a comorbidity index, a self-health index and a healthcare use index as follows.

The comorbidity index included data on 22 comorbidities (hypertension, diabetes mellitus, renal impairment, asthma, anemia, arthritis, coronary heart disease, angina, previous myocardial infarction, previous stroke, emphysema, thyroid disease, obesity, chronic bronchitis, liver disease, malignancy, osteoporosis, previous hip fracture, previous wrist fracture, previous spine fracture, cognitive impairment and overnight hospitalization). The index was calculated as the sum of the total number of comorbidities reported divided by the maximum number possible (22). The self-health index was calculated based on two questions reporting on a subject’s general health condition and on their current health compared to 1 year ago. Options for the general health question were: ‘good general health’ (or better), ‘fair general health’ or ‘poor general health’. Options for the question regarding current health compared to 1 year ago were: ‘better current health’, ‘about the same’ or ‘worse current health’. Affirmative answers were scored as 1, and negative answers were scored as 0. An aggregate index was generated according to the following formula: self-health index = ((‘fair general health’ × 2) + (‘poor general health’ × 4)) × (1 – (‘better current health’ × 0.5) + (‘worse current health’)). Therefore, subjects who became more ill would receive twice the penalty for current health status, whereas subjects who reported recovery would receive a modifier of 0.5. The healthcare use index is the number of times a subject received healthcare over the past year as coded by the NHANES variable ‘HUQ050’^35^. These three indices were included directly as clinical parameters without normalization.

After excluding any parameters with more than 10% missing observations and all subjects with incomplete records, the resulting dataset was reduced to 3,811 participants and 165 clinical parameters. Our final training cohort, composed of the NHANES IV 1999–2000 study participants, included 923 males and 852 females, and our testing cohort, composed of the NHANES IV 2001–2002 study participants, included 1,094 males and 942 females (see Supplementary Table 2 for baseline characteristics).

It was showed previously that subjects with missing data in some cases are not completely random. For example, older subjects may be more likely to have missing data in some variables^48^. This is a potential concern, as removal of subjects with missing values may affect some subsets of the cohort more than others. We, therefore, opted to remove features with more than 10% missing values. Because of its biological importance, NT-proBNP was included despite being missing in more than 10% of subjects (14.4%).

### BA—definition used and determination from hazard ratio

We know that individual BA typically differs from CA^2^. Some approaches define the BA of an individual as the age at which that individual’s position in feature space would be approximately normal for a reference cohort^27–29^. A different approach uses mortality risk as the dependent variable, directly building models to predict future mortality from biological parameters^17,18,31–33^. In this scheme, the true BA of an individual is defined as the ‘Gompertz age’ of the reference cohort at which subjects of the reference cohort have the same all-cause mortality risk as the individual in question^29,63^. This definition of BA addresses the problem that different clocks (for example, based on different feature spaces or using different mathematical/machine learning methods) often do not agree with each other, sometimes producing vastly different BA estimates for the same individual. This might occur because the appearance of an individual may be different when viewed through the lens of different feature spaces and compared to different training cohorts. However, clocks trained to predict ‘Gompertz’ BA can be objectively compared by testing them directly against the ground truth of historically observed all-cause mortality. Here, we adopt ‘Gompertz’ BA, following the approach by Levine et al.^18^.

We first generated two Cox proportional hazard models for the training cohort^64^. The NULL models for males and females were fitted to predict the mortality hazard (h_0_) of dying over the follow-up period, based on CA and sex alone. This NULL model also yields the sex-specific mortality rate doubling time (MRDT_sex_) for the training cohort. A second Cox model was then constructed by taking into consideration the covariates (PCs) for each subject of the training cohort. The PCs to be included were selected based on the percentage explained (see below). The final model was then used to predict the hazard of dying as a function of an individual’s position in PC-transformed feature space (h_pc_). Finally, differences in ‘Gompertz age’ are calculated that result in an equivalent relative hazard ratio h_pc_/h_0_, thereby converting the hazard ratio into a corrected ‘Gompertz age’ (Δage) as follows:

$$\Delta {\rm{age}}=\frac{\mathrm{ln}\left(\frac{{h}_{{pc}}}{{h}_{0}}\right)}{\mathrm{ln}(2)}\bullet {MRD}{T}_{{sex}}$$

The final BA was then calculated by adding this age correction to the subject actual CA:

$${\rm{BA}}={\rm{CA}}+\Delta {\rm{age}}$$

### SVD/PCA—motivation and construction

When building models based on only a small subset of features, errors within these selected features can have a large impact on the resulting model predictions. An alternative approach, therefore, is to employ dimensionality reduction techniques—that is, transformation of data from a high-dimensional feature space into an approximately equivalent, lower-dimensional space. Using this lower-dimensional space as the basis of the model simplifies model building (reduces the number of parameters that need to be fitted), but, because the original features are still included in determining each subject’s position in the reduced feature space, errors in individual parameters have a less pronounced effect on the overall model. Here, we explored the potential of PCA as a linear dimensionality reduction tool. For the construction of PCAge and LinAge, we first normalized the clinical parameters of the training dataset into z-scores, before transforming them into PC coordinates using the SVD function of R version 4.2.0 (https://www.R-project.org/). For the testing and validation cohorts, we used the singular vectors derived for the training cohort to project each subject’s normalized feature space coordinates into the same PC coordinate system of the training set.

### PCAge and LinAge development and validation

For PCAge, the first 18 PCs of the training data, which accounted for 99% of the overall variability in that dataset, together with each subject’s CA, were selected as covariates in the Cox proportional hazards regression model, trained against data from 20-year mortality follow-up. The hazard ratio for each individual was then converted into an age correction as outlined above. Separate BA clocks were trained for males and females, exclusively using the data for the NHANES IV 1999–2000 recruitment wave and tested against the NHANES IV 2001–2002 wave.

For the construction of LinAge, we selected 61 parameters that are routinely measured clinically or can be extracted from clinical records. The relevant parameters are listed in Supplementary Tables 1 and 7. With the exception of CA, basophils number, smoking status and morbidity indices, each parameter was normalized with reference to its median value obtained from the ‘healthy aging’ clusters of PCAge (Fig. 3a,b). Parameters were normalized separately for male and females by subtraction of the median and division by the median absolute deviation (MAD) in the ‘healthy aging’ clusters for males or females, respectively (Supplementary Table 7). Smoking status was determined by binning the serum cotinine levels according to known cutoffs demonstrated in previous studies to correspond with qualitative smoking status^65^: 0 to <10 ng ml^−1^ (non-smokers = 0), 10 to <100 ng ml^−1^ (light smokers = 1), 100 to <200 ng ml^−1^ (moderate smokers = 2) and ≥200 ng ml^−1^ (heavy smokers = 3). This approach is subject to less recall bias when compared to using questionnaire data on smoking status. However, in cases where data on cotinine are not available, this score can also be populated directly from data obtained by questionnaire without any change to LinAge. As for PCAge, feature space coordinates were transformed into PC space, and models were optimized separately for males and females based on the mortality follow-up for the training cohort.

For LinAge, PCs were selected for inclusion in the final model using regularized Cox regression using ‘glmnet’ version 4.1–7 (refs. ^66,67^) and the ‘survival’ package version 3.5–8 (ref. ^68^) in R, with a 10-fold cross-validation and for alpha values of 1, 0.75 and 0.5 (Supplementary Fig. 9). PCs bearing non-zero weights, which were identified in a minimum of five instances out of the 100 iterations and consistently selected across all models, were selected for inclusion in the final Cox model. Individual proportional hazard ratios were transformed into age deltas and added to CA for each subject as described above. For LinAge, we also extracted a version of the clock parametrized in the original (non-PC) feature space by multiplying the SVD-derived coordinate transformation matrix with the weight matrix in PC space to obtain discrete parameter weights for each of the 61 parameters included in LinAge. These individual, parameter-level weights for the clinical parameters can be found in Supplementary Table 7 and enable LinAge to be calculated directly from parameter values using only a spreadsheet.

Equation for BA:

$${\rm{LinAge}}(\bar{X}\,)={\beta }_{{CA}}\bullet {CA}+\mathop{\sum }\limits_{i=1}^{n}{\beta }_{i}\bullet {X}_{i}^{\,z}+{C}_{0}$$

where:

$\bar{X}=\left\{{X}_{i}\right\}$, vector of *n* = 61 parameters used for LinAge (for given subject)

${\bar{X}}^{\,z}=\left\{{X}_{i}^{\,z}\right\}$, ${X}_{i}^{\,z}=\frac{{X}_{i}-{{median}}_{i}}{{{MAD}}_{i}}$, normalized parameters (for given subject)

*median*_*i*_ = sex-specific median value for *i-*th parameter over ‘healthy aging’ cluster (Supplementary Table 7)

*MAD*_*i*_ = sex-specific MAD value for *i-*th parameter over ‘healthy aging’ cluster (Supplementary Table 7).

### Further LinAge validation

#### Validation in independent NHANES III cohort

The NHANES III study ran from 1988 to 1994 to assess the health and nutritional status of the United States’ civilian, non-institutionalized population^69^. Cross-sectional survey data included demographic, socioeconomic and dietary information; responses to health-related questions; medical and physiological measurements; and results of clinical laboratory tests. NHANES III was approved by the National Center for Health Statistics Research Ethics Review Board. Anonymized data from NHANES III were obtained from a publicly available source^69^. Linked mortality data were obtained from the National Death Index^62^ and are available from 18 October 1988 until 31 December 2019. Our final NHANES III external validation cohort comprised 715 males and 819 females aged 40–89 years for whom most LinAge parameters were available.

Of all LinAge parameters, only one (NT-proBNP) was not recorded as part of NHANES III and was, therefore, missing from the entire NHANES III dataset. We addressed this issue by setting the weights associated with NT-proBNP to zero in the LinAge model. This approach is conservative with respect to evaluating LinAge in a different cohort, as LinAge would perform worse in NHANES IV if the information encoded in the NT-proBNP was not used. We next compared individual parameters between NHANES III and NHANES IV to identify major batch effects. However, we deliberately did not carry out a formal batch or range correction procedure as this would not generally be feasible, for example, when applying LinAge to a new patient cohort for which exact parameter ranges may not be known. However, we identified technical issues with the CRP and bicarbonate parameters. For technical reasons, CRP values in NHANES III had a lower detection limit of 0.21, whereas NHANES IV had a lower detection limit of 0.01. This means that the lowest possible value in NHANES III would be considered above the detection limit and within the modeled range for NHANES IV. We addressed this issue by replacing CRP values for group of subjects with the median log(CRP) value from NHANES IV, thereby ensuring that, for this subset of subjects, CRP contributed a zero value to the LinAge Delta age. Finally, the distribution of bicarbonate values of NHANES III was systematically and substantially higher than in NHANES IV (Supplementary Fig. 10a). Although some other parameters also showed systematic differences between NHANES III and NHANES IV, we chose to correct these batch effects only for bicarbonate alone by re-centering the NHANES III bicarbonate distribution on NHANES IV by multiplication of the bicarbonate values of NHANES III by the ratio between the mean bicarbonate values of the ‘healthy aging’ clusters in the NHANES IV 1999–2002 cohorts and NHANES III (Supplementary Fig. 10b). No other corrections were applied.

#### Evaluation of the CALERIE intervention trial

Details of the CALERIE phase 2 multi-center randomized controlled trial were reported and can be found in the original publications^55,70,71^. The CALERIE trial is registered on ClinicalTrials.gov as NCT00427193. CALERIE received ethics approval at three clinical centers (Washington University School of Medicine, Pennington Biomedical Research Center and Tufts University) and at the coordinating center at Duke University. Data from CALERIE are publicly available (https://calerie.duke.edu), and all study participants are de-identified.

When contemplating applying LinAge to CALERIE, we discovered that several of the clinical parameters used in LinAge were not present in the CALERIE dataset. We applied our pipeline to re-train a version of LinAge based on a subset of the feature space for which parameters were available within both NHANES IV and CALERIE. This clock included only features found in both the NHANES IV 1999–2002 waves and the CALERIE trial (Supplementary Table 1). The resulting CALinAge clock was trained in subjects aged 20–70 years in the NHANES IV 1999–2000 cohort (*n* = 1,516), before being validated in subjects aged 20–70 years in the separate NHANES IV 2001–2002 cohort (*n* = 1,683) and finally applied to the CALERIE trial subjects (*n* = 159). Our final CALERIE external validation cohort comprised 101 CR and 58 AL subjects.

### Clustering analyses

We performed *k*-means clustering, using the ‘cluster’^72^ (version 2.1.4) and ‘factoextra’^73^ (version 1.0.7) R packages, by Euclidean distance in PC coordinates for 2,017 male and 1,794 female participants from the entire NHANES IV 1999–2002 dataset. We selected PC numbers 2, 3, 4, 7, 10, 11, 13, 17 and 18 for clustering, based on their significant weights in the PCAge model (Supplementary Fig. 3). We generated five distinct clusters each for males and females. The optimal number of clusters was determined to minimize the degree of overlap in information/themes between clusters. A larger number of clusters resulted in additional separation along the cardio-metabolic axis, whereas selecting fewer clusters resulted in merger along the same axis. Clusters were visualized using the ‘fviz_cluster’ function of ‘factoextra’. This method projects subjects into a two-dimensional (2D) plane which forms the *x*–*y* plane of the cluster diagrams (Fig. 3a,b). The third (*z*) axis of the three-dimensional (3D) cluster diagram is time, in this case the age of each subject at the time of final mortality status update (31 December 2019). If this update is a record of death (subject deceased), a small marker (sphere) is drawn. If the final record is a record of survival to the end of the follow-up period, then the age at that timepoint is used as the *z* coordinate, and a large marker is drawn.

### PC interpretation and partial correlation network analysis

Partial correlation network analysis of PC4 was performed by selecting the top 10% clinical measures by absolute magnitude of weights within PC4. For these parameters, we generated partial correlations using the ‘ppcor’^74^ (version 1.1) R package. Edges below a hard threshold of 0.1 were set to zero, and the remaining edges were used as edge weights to construct a network using the ‘igraph’^75,76^ (version 2.0.3) R package. Edges indicating positive correlation are colored in blue, and negative edges are colored in red. Clinical parameters were categorized by body composition, physiological functions and physiological responses, based on domain knowledge to aid interpretation of the partial correlation network.

### PhenoAge, ASCVD and CFS scores

PhenoAge^18^ and the ASCVD score^34^ were constructed, and functions to calculate them from the data matrix were implemented, based on the equations provided in the original publications. CFS scores were determined as previously reported^54^.

### Dealing with missing values

Missing data is a common challenge with clinical datasets. When variables are missing from all or a large fraction of subjects, it may be necessary to remove that variable and train a new (‘custom’) clock. An example workflow for creating a custom PCA clock is illustrated in Code and Supplementary Files (Supplementary Information). A similar toolkit for clocks based on individual parameters is already available as an R package^28^.

If features are missing randomly for only a small fraction of subjects, missing values in continuous datasets can be imputed, for example, using mean imputation, random forest imputation and PCA-based imputation algorithms. PCA-based imputation takes advantage of the fact that the PC coordinates are linearly uncorrelated. For PCA-based imputation, the original data are projected into the PC coordinate system, initially using randomly imputed values for the missing data. The missing values are then deduced by reverse projecting the reduced data back onto the original space. This cycle is repeated, iteratively refining the missing values. Such PCA imputation can be performed using the iterative PCA algorithm implemented in the ‘missMDA’ package. We explored PCA imputation as implemented in the ‘missMDA’^77^ (version 1.18) R package with 10,000 iterations. However, when imputing data, care needs to be taken to ensure that variables are missing completely at random, with the probability of being missing not related to parameter values. For our final analysis (both PCAge and LinAge), we elected not to impute missing values and, instead, removed subjects with missing values from the analysis completely.

For LinAge itself, a zero z-score can be substituted for missing values after normalization, thereby setting the impact of the missing value on LinAge to zero. The magnitude of error introduced by this substitution (in years) is of the order of the individual weight (Supplementary Table 7) for the parameter in question.

### Statistics and reproducibility

For the NHANES IV cohort, we excluded (1) participants top-coded at age 85 years, as we could not ascertain the exact CAs of these adults; (2) participants who died from accidental deaths, as these were deemed to be not age related; and (3) physiological and laboratory measurements with significant missing data, defined as more than 10% of the training dataset. For the NHANES III cohort, we excluded (1) participants top-coded at age 90 years, as we could not ascertain the exact CAs of these adults; (2) participants who died from accidental deaths, as these were deemed to be not age related; and (3) subjects for whom laboratory measurements needed to calculate LinAge were missing. For the CALERIE cohort, participants with missing data for whom CALinAge could not be calculated were excluded. For all three cohorts, no statistical method was used to pre-determine sample size.

Correlation analyses were performed using linear regression, and the strength of correlation was determined using PCC. Two-sided *t*-tests were used to compare the delta telomere lengths, delta digit symbol substitution test scores and delta gait speeds between groups and to compare the PCAge Deltas of centenarians to non-centenarians. Two-way ANOVA was used to compare aging rates between the AL and CR groups in the CALERIE trial. Survival analyses were performed using log-rank tests. Kruskal–Wallis tests were performed on continuous variables during cluster characterization. Post-test pairwise comparisons using Wilcoxon rank-sum tests with continuity correction were performed between clusters. Hypergeometric probability distributions were used to compare categorical variables during cluster characterization. Kruskal–Wallis tests were used to compare clinical parameters between multiple groups involving healthy subjects, untreated subjects with high urine ACR and ACE-I/ARB-treated subjects. Post hoc analyses were performed using Dunn’s test. ROC curves were compared using DeLong’s test. All statistical analyses were performed using R version 4.2.0 (https://www.R-project.org/).

### Reporting summary

Further information on research design is available in the Nature Portfolio Reporting Summary linked to this article.

### Supplementary information

Supplementary information Supplementary Figs. 1–10, Supplementary Tables 1–8, Cluster Analysis, Code and Supplementary Files

Reporting Summary

Supplementary Data 1 PCAge: Cleaned and merged dataset derived from NHANES IV 99/00 and 01/02 recruitment waves. Includes mortality linkage. This dataset is sufficient to train the PCAge clock example.

Supplementary Data 2 PCAge: Codebook file lists features (columns in NHANES datafile), linking NHANES variable names to human readable names. Codebook also contains columns used to include and force-include variables in clock.

Supplementary Code 1 PCAge: Example code (R script) constructing and displaying a PCAge clock—needs codeBook.csv and nhanesMerged.csv in the working directory.

Supplementary Data 3 linAge_XLS: Excel spreadsheet to calculate LinAge from clinical parameters.

Supplementary Data 4 linAge_XLS: Readme file with instructions how to use linAge_Example.xls spreadsheet.

Supplementary Data 5 linAge_Rscript: Data matrix extracted from NHANES IV 99/00 and 01/02 required to run linAge.R example script.

Supplementary Data 6 linAge_Rscript: Data matrix (normalized) extracted from NHANES IV 99/00 and 01/02 required to run linAge.R example script.

Supplementary Data 7 linAge_Rscript: Data matrix extracted from NHANES IV 99/00 and 01/02, including demographic and questionnaire data, required to run linAge.R example script.

Supplementary Data 8 linAge_Rscript: Data file containing LinAge parameters and normalization parameters for LinAge. Required to run linAge.R example script.

Supplementary Software 2 linAge_Rscript: R script illustrating LinAge calculation from parameter and data files.

Supplementary Data 9 customClock: Readme file explaining purpose and flow of customClock_script.R.

Supplementary Software 3 customClock: Main custom clock R script, illustrating construction of LinAge using a subset of variables (see README_2.txt).

Supplementary Data 10 customClock: Data matrix extracted from NHANES IV 99/00 and 01/02 required to run customClock_script.R example script (see README_2.txt).

Supplementary Data 11 customClock: Codebook file lists features (columns in NHNAES datafile), linking NHANES variable names to human readable names. Codebook also contains columns used to include and force-include variables in clock. Needed to run customClock_script.R (see README_2.txt).

Supplementary Data 12 customClock: File containing modifiable parameters for customClock_script.R (see README_2.txt).

## Data Availability

All datasets used are publicly available online at https://wwwn.cdc.gov/nchs/nhanes/Default.aspx, https://wwwn.cdc.gov/nchs/nhanes/nhanes3/datafiles.aspx#core and https://calerie.duke.edu. There were no restrictions on data availability. This study was reported according to STROBE guidelines for cohort studies^78^.

## Code availability

The codes for PCAge and LinAge, as well as code to generate a custom PC clock, are available as zipped R archives. Content of this archive and the readme file is provided in the Supplementary Information.
