# Supplementary material for: Principal component-based clinical aging clocks identify signatures of healthy aging and targets for clinical intervention
Source: Nat Aging. 2024 Jun 19;4(8):1137–52. doi: 10.1038/s43587-024-00646-8 (PMC11333290; doi:10.1038/s43587-024-00646-8)
Supplement: Supplementary file 1 — Supplementary Figs. 1–10, Supplementary Tables 1–8, Cluster Analysis, Code and Supplementary Files [file 43587_2024_646_MOESM1_ESM.pdf]

nature aging

Supplementary information https://doi.org/10.1038/s43587-024-00646-8

Principal component-based clinical aging clocks identify signatures of healthy aging and targets for clinical intervention

In the format provided by the authors and unedited

Supplementary Information

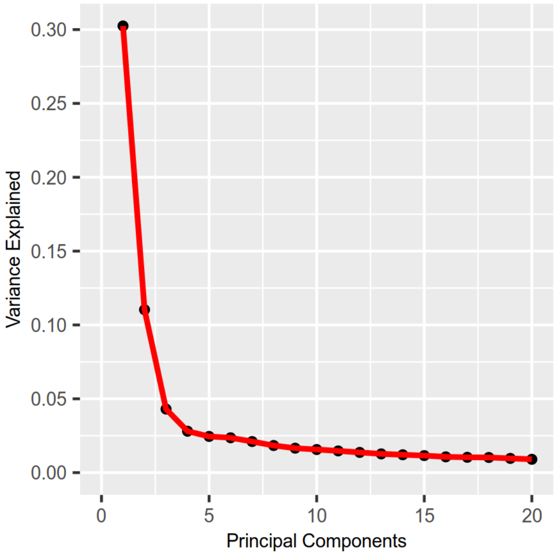

Supplementary Fig. 1: Scree plot of the first 20 PCs. The first 18 PCs accounted for 99% of the overall variance in the NHANES IV 1999-2000 training dataset. The remaining PCs are not shown because they contribute very little (<1%) to the overall variance.

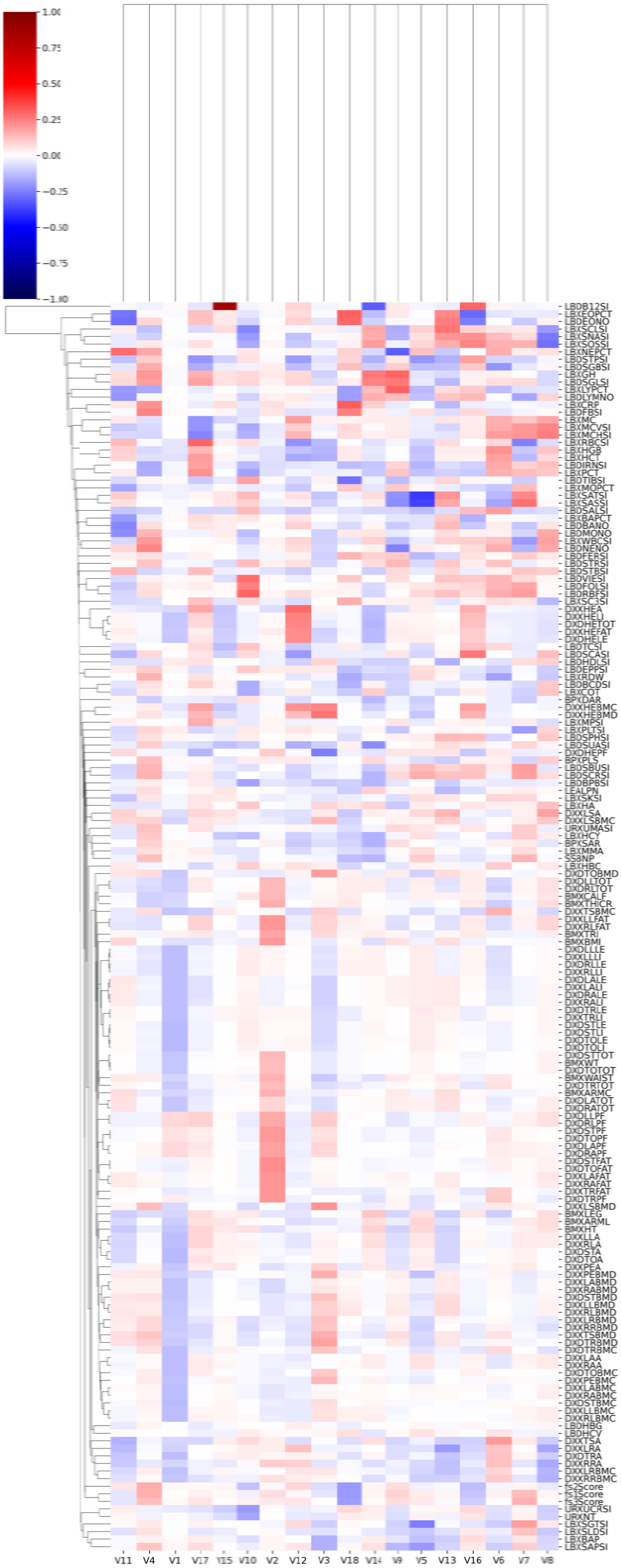

Supplementary Fig. 2: Heatmap illustrating the loadings of the 165 clinical parameters onto the 18 PCs with significant weights in PCAge. We first selected the 18 PCs (V1 to V18) with significant weights in the PCAge Cox models and extracted the corresponding right singular vectors (rotations) for each of these 18 PCs. To visualize the loadings of the clinical parameters on each of these PCs, we applied hierarchical clustering analysis to the 165 parameters before visualizing the re-ordered parameter weights (rotations) as a heatmap.

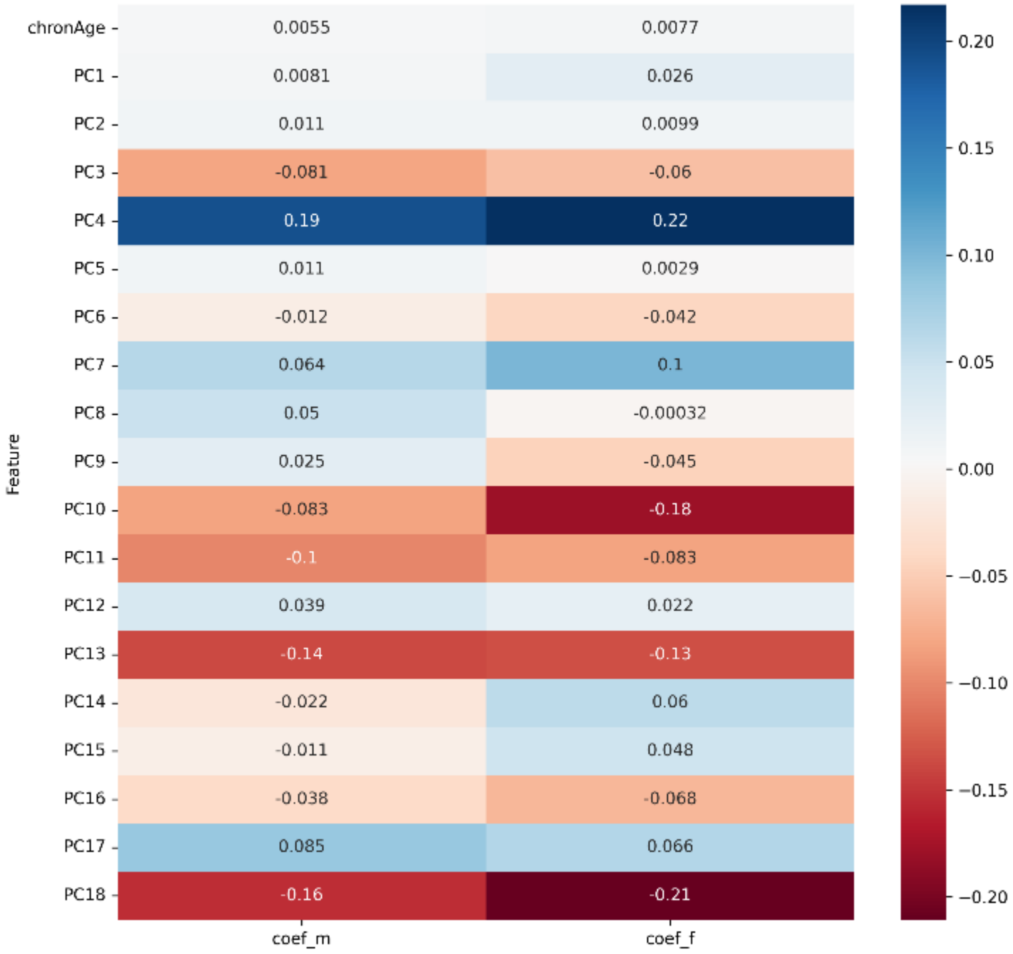

Supplementary Fig. 3: Heatmap illustrating the weights assigned to PC1-18 in PCAge for males and females. This heatmap shows the subset of the 18 PCs that had significant weights in PCAge. PC4 was the PC with the highest weight in both the male (coef_m) and female (coef_f) Cox proportional hazard models.

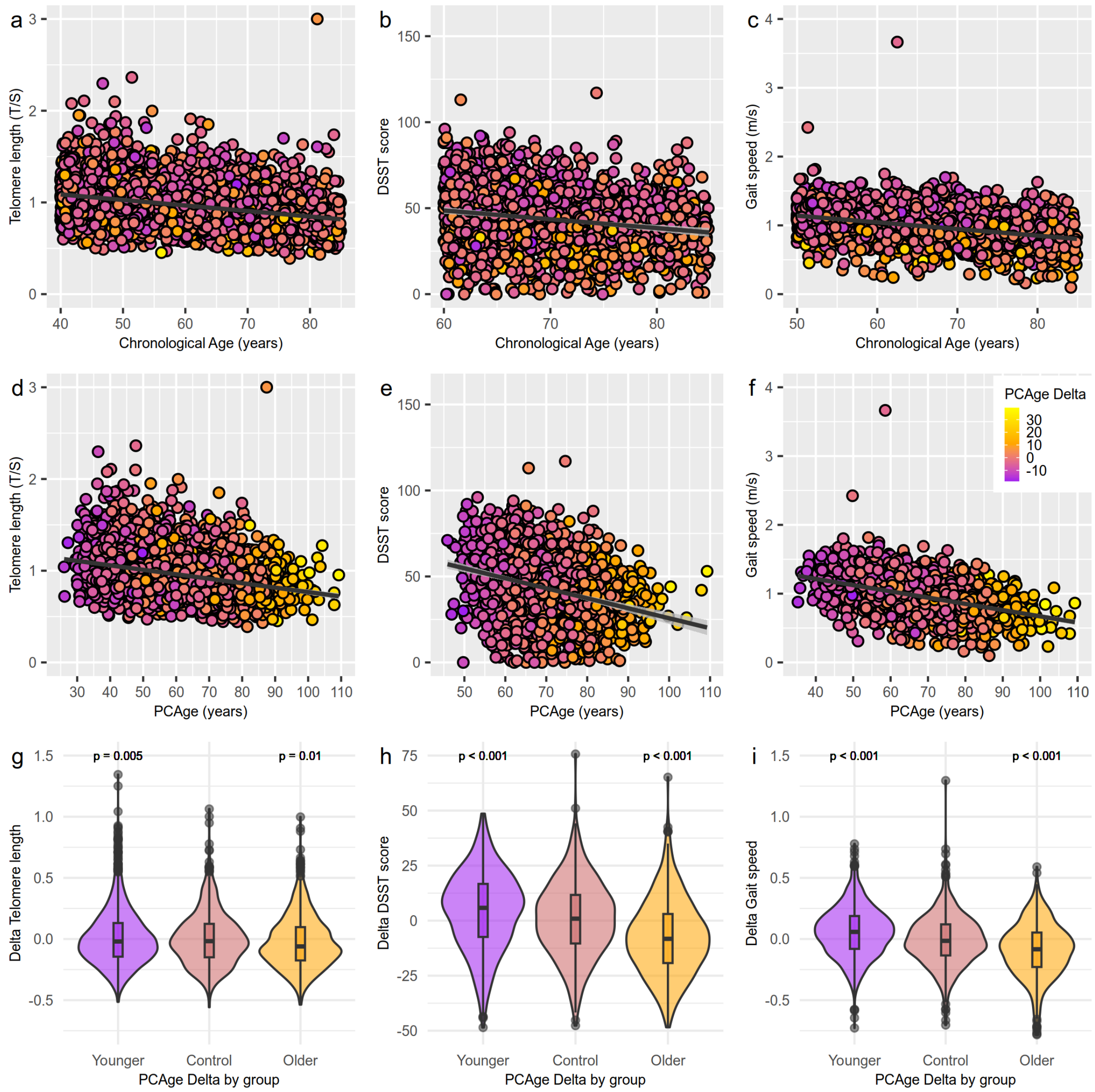

Supplementary Fig. 4: PCAge predicts molecular, cognitive, and functional parameters known to depend on BA. a-f, Scatter plots and linear regression of CA or PCAge against telomere length, digit symbol substitution test (DSST) score (a cognitive measure), and gait speed (a functional measure), respectively. Each point represents a unique subject and is filled by a color that represents the PCAge Delta of that subject, which ranges from purple (-10) to yellow (+30). In both the training and testing cohorts, CA was negatively correlated with telomere length (R^2=0.09, P<0.001, n=3,620), DSST score (R^2=0.04, P<0.001, n=1,755) and gait speed (R^2=0.14, P<0.001, n=2,682). As PCAge includes CA, unsurprisingly, in both the training and testing cohorts, PCAge was negatively correlated with telomere length (R^2=0.09, P<0.001, n=3,620), DSST score (R^2=0.11, P<0.001, n=1,755) and gait speed (R^2=0.22, P<0.001, n=2,682). Additionally, however, the PCAge Deltas themselves were also significantly negatively correlated with telomere length (R^2=0.01, P<0.001, n=3,620), DSST score (R^2=0.08, P<0.001, n=1,755) and gait speed (R^2=0.11, P<0.001, n=2,682). **g-i,** Violin plots of PCAge Delta categorized into younger, control and older groups plotted against the delta scores for telomere length (n=3,620), DSST score (n=1,755), and gait speed (n=2,682). Violin outline displays the continuous distribution of values for each box plot. Center line of box indicates the median value. Lower and upper hinges correspond to 25th and 75th percentiles, respectively. Whiskers extend to $\pm$1.5 multiplied by inter-quartile range with points outside this range drawn individually. The control group represented the middle 50% of all subjects, and hence, the reference group, to which the younger (best 25% quartile) and older (worst 25% quartile) groups were compared by two-sided t-tests. Compared to control, the biologically younger subjects with large negative (bottom 25% quartile) PCAge Deltas had significantly longer telomeres than expected for their CA (significant larger positive Delta Telomere

lengths, *P*=0.005), significantly higher cognitive scores (significantly larger positive Delta DSST scores, *P*<0.001) and walked significantly faster than expected (significantly larger Delta Gait speeds, *P*<0.001). By contrast, biologically older subjects, with the largest positive (top 25% quartile) PCAge Deltas had significantly shorter Delta Telomere lengths (*P*=0.01), significantly lower DSST cognitive scores (*P*<0.001) and significantly slower Delta Gait speeds (*P*<0.001). Taken together, our data therefore suggest that PCAge predicts molecular, cognitive, and functional parameters beyond that of simple CA.

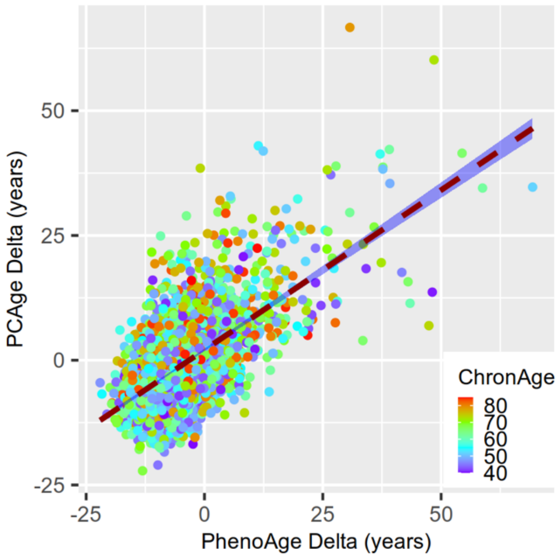

Supplementary Fig. 5: There were significant residuals between PCAge and PhenoAge. Scatter plot and linear regression of PhenoAge Deltas (residuals between CA and PhenoAge) versus PCAge Deltas (residuals between CA and PCAge). The residuals between PCAge and PhenoAge were moderately correlated (Pearson correlation coefficient=0.61). The color gradient (ChronAge) reflects the CAs of the subjects. Areas shaded in color indicate 95% error band for linear fit.

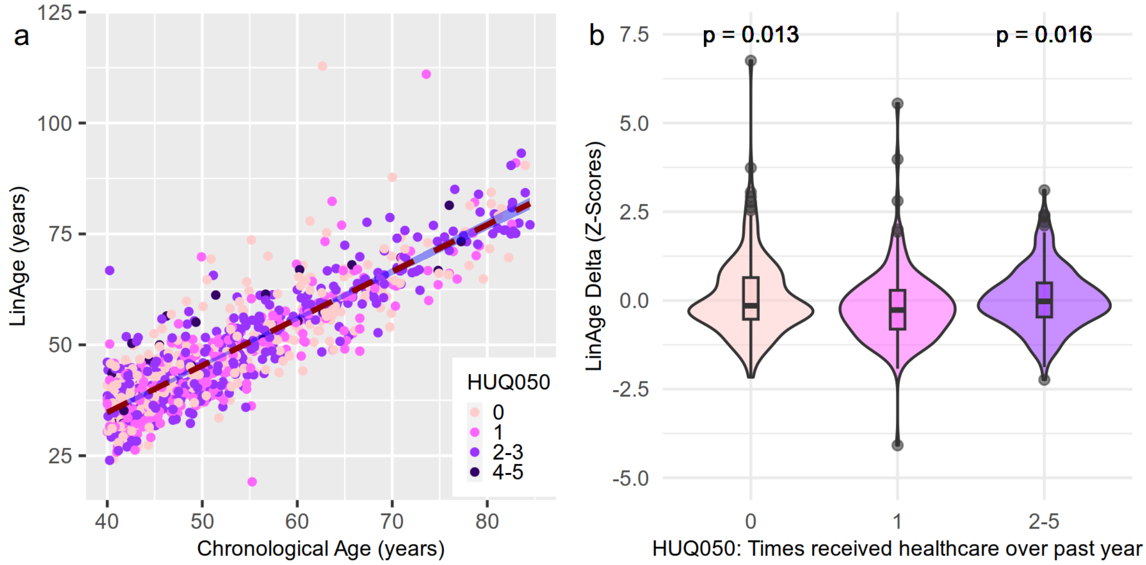

Supplementary Fig. 6: Subjects without comorbidities but with frequent healthcare visits were biologically older. a, Scatter plot of CA versus LinAge for subjects with a co-morbidity index score of 0 (*n*=845). HUQ050 refers to the number of times each subject received healthcare over the past year. Category 0 refers to 0 visits (*n*=273), category 1 refers to 1 visit (*n*=236), category 2-3 refers to 2-9 visits (*n*=311), category 4-5 refers to 10 or more visits (*n*=25). Most subjects with 10 or more healthcare visits over the past year were biologically older. **b,** Violin plots of number of healthcare visits over the past year grouped into 0 (0 visit) (*n*=845), 1 (1 visit) (*n*=236), and 2-5 (2 or more visits) (*n*=336) categories plotted against the z-scores for LinAge Delta. To adjust for the effects of the dispersion of LinAge Deltas, which increase with CA, z-scores were calculated for each subject by subtracting the mean of the LinAge Delta for each CA category (45-54, 55-64, 65-74, and 75-84 years) and then dividing by the standard deviation of the LinAge Delta for that CA category. Between all groups, one-way ANOVA (*P*=0.016) and post-test comparisons were all statistically significant, and the group with 1 healthcare visit over the past year had the lowest mean LinAge Delta z-score. Violin outline displays the continuous distribution of values for each box plot. Center line of box indicates the median value. Lower and upper hinges correspond to 25th and 75th percentiles, respectively. Whiskers extend to $\pm$1.5 multiplied by inter-quartile range with points outside this range drawn individually.

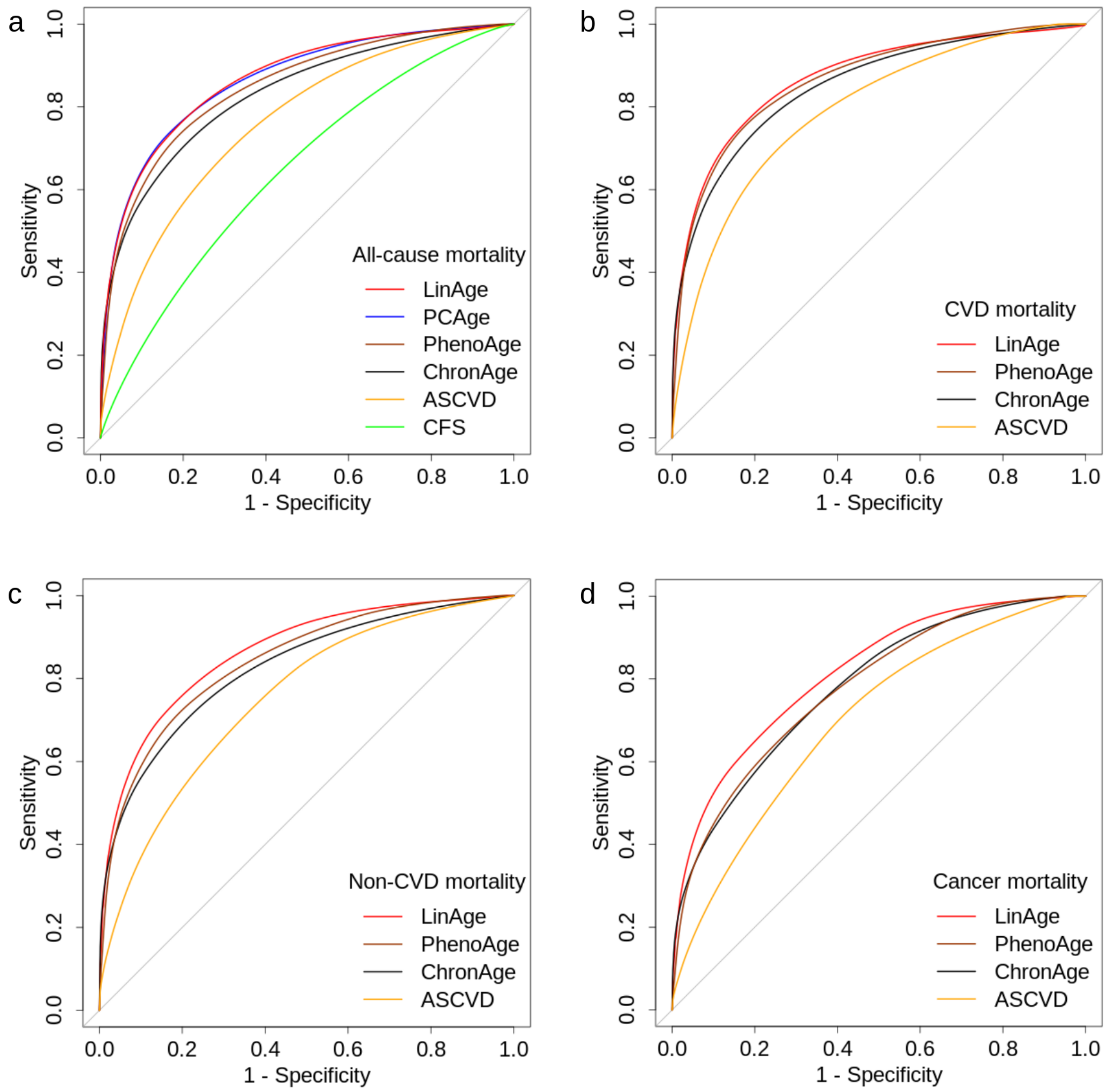

Supplementary Fig. 7: ROC curves for 20-year mortality prediction. a, ROC curves for 20-year all-cause mortality for LinAge (red), PCAge (blue), PhenoAge (brown), ChronAge (black), ASCVD (yellow) and CFS (green) scores. Refer to Fig. 5c for details. **b,** ROC curves for LinAge (red), PhenoAge (brown) and the ASCVD (yellow) score for predicting 20-year CVD-related (heart disease and stroke) mortality. Both LinAge (red) (AUC=0.8683) and PhenoAge (brown) (AUC=0.8627) significantly outperformed the ASCVD (yellow) score (AUC=0.7866) (*P*=0.0058 and *P*=0.012, respectively) in predicting 20-year CVD-related mortality. However, there were no significant differences between LinAge (red) and PhenoAge (brown) in predicting 20-year CVD-related mortality. **c,** ROC curves for LinAge (red), PhenoAge (brown) and the ASCVD (yellow) score for predicting 20-year non-CVD-related mortality. LinAge (red) (AUC=0.8643) significantly outperformed PhenoAge (brown) (AUC=0.8411, *P*<0.001) and the ASCVD (yellow) score (AUC=0.7479, *P*<0.001) in predicting 20-year non-CVD-related mortality. **d,** ROC curves for LinAge (red), PhenoAge (brown) and the ASCVD (yellow) score for predicting 20-year cancer-related mortality. LinAge (red) (AUC=0.8148) also significantly outperformed PhenoAge (brown) (AUC=0.7779, *P*<0.001) and the ASCVD (yellow) score (AUC=0.6938, *P*<0.001) in predicting 20-year cancer-related mortality. ROC curves were compared by DeLong's test.

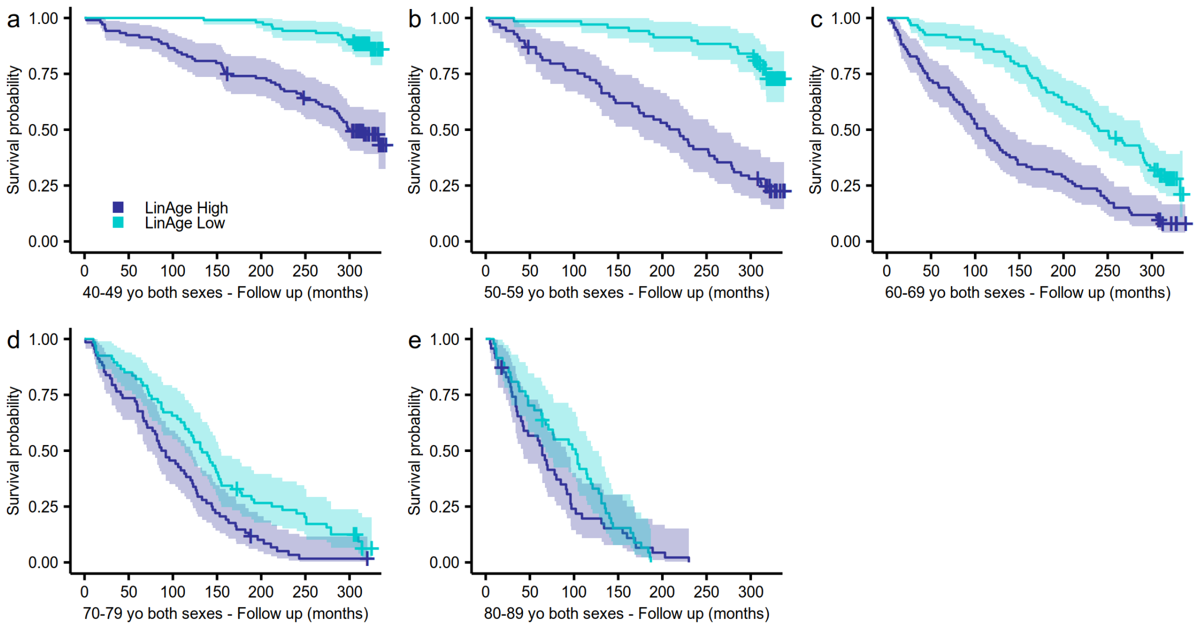

Supplementary Fig. 8: LinAge also predicts BA in the NHANES III external validation cohort. Kaplan-Meier survival curves comparing actual survival in the NHANES III validation cohort over a 25-year follow up period. Male and female subjects are pooled and survival for each CA bin is compared between individuals that are significantly biologically younger (bottom 25% quartile for LinAge Delta, LinAge Low) or significantly biologically older (upper 25% quartile for LinAge Delta, LinAge High) than their CA. In the 40-49 (**a**), 50-59 (**b**), 60-69 **(c)**, and 70-79 (**d**) CA bins, LinAge was able to identify and separate subjects who aged unusually well (LinAge Low) or badly (LinAge High), as evidenced by the clear separation of survival curves for these two groups ($P<0.001$). For male and female subjects in the 80-89 CA bin, the difference did not reach statistical significance ($P=0.4$). Areas shaded in color in each panel indicate 95% error bands for lines of the same color.

a **b**

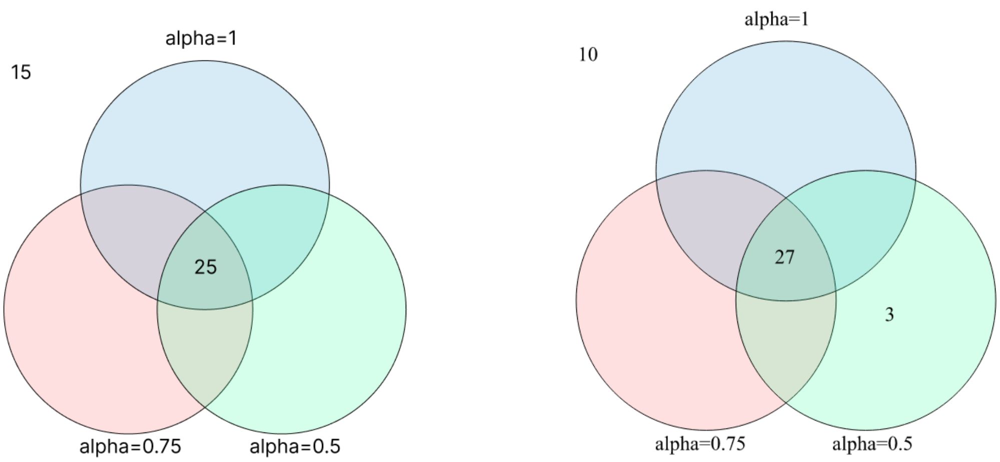

LinAge (male)				LinAge (female)			
**PC number**	**alpha=1**	**alpha=0.75**	**alpha=0.5**	**PC number**	**alpha=1**	**alpha=0.75**	**alpha=0.5**
PC1	1	1	1	PC1	1	1	1
PC3	1	1	1	PC3	1	1	1
PC4	1	1	1	PC4	1	1	1
PC5	1	1	1	PC6	1	1	1
PC6	1	1	1	PC7	1	1	1
PC7	1	1	1	PC8	1	1	1
PC8	1	1	1	PC9	1	1	1
PC9	1	1	1	PC10	1	1	1
PC10	1	1	1	PC11	1	1	1
PC11	1	1	1	PC12	1	1	1
PC12	1	1	1	PC13	1	1	1
PC13	1	1	1	PC15	1	1	1
PC14	1	1	1	PC17	1	1	1
PC15	1	1	1	PC18	1	1	1
PC17	1	1	1	PC19	1	1	1
PC18	1	1	1	PC20	1	1	1
PC19	1	1	1	PC21	1	1	1
PC20	1	1	1	PC22	1	1	1
PC21	1	1	1	PC23	1	1	1
PC22	1	1	1	PC26			1
PC27	1	1	1	PC27	1	1	1
PC31	1	1	1	PC28			1
PC32	1	1	1	PC30	1	1	1
PC35	1	1	1	PC31	1	1	1
PC36	1	1	1	PC33	1	1	1
				PC34	1	1	1
				PC35	1	1	1
				PC36	1	1	1
				PC37	1	1	1
				PC38			1

Supplementary Fig. 9: Venn diagrams of PCs selected by elastic net regression for inclusion into the LinAge model for different alpha values. a, For males, the PC coefficients of the models for different alpha values overlapped for all PCs indicating there is a clear signal in males. **b,** For females, all models agreed on 27 PCs. However, alpha=0.5 also selected 3 additional PCs (PC26, PC28 and PC38). This indicates that there is no complete agreement regarding the importance of the additional PCs. However, the common 27 PCs indicate a consensus signal and were ultimately used for the final model.

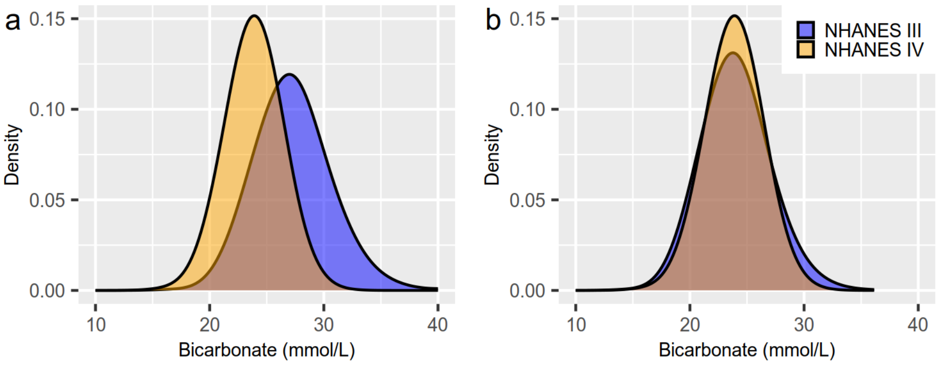

Supplementary Fig. 10: The distribution of bicarbonate values in NHANES III was re-centered on the NHANES IV 1999-2002 cohorts. a, The bicarbonate values in NHANES III were systematically higher than in NHANES IV with a substantial number of subjects having bicarbonate values above 30 mmol/L which is outside of the usual medically acceptable reference range. **b,** To re-center the NHANES III bicarbonate distribution on NHANES IV, we first calculated the ratio of the mean bicarbonate values between the subjects in the "healthy aging" clusters in the NHANES IV 1999-2002 cohorts and NHANES III. We then multiplied the bicarbonate values from NHANES III by this ratio, resulting in a distribution that was similar to NHANES IV. This correction means that the average subject in NHANES III receives a zero delta age contribution from the bicarbonate term.

Supplementary Table 1. List of the clinical parameters used in PCAge, LinAge and CALinAge

NHANES 1999-2000 Variable Name	Parameter	PCAge	LinAge	CALinAge
BPXPLS	60 second pulse (30 second pulse x 2)	√	√	√
BPXSAR	Systolic Blood Pressure average reported to examinee	√	√	√
BPXDAR	Diastolic Blood Pressure average reported to examinee	√	√	√
BMXWT	Weight (kg)	√		√
BMXHT	Standing Height (cm)	√		√
BMXBMI	Body Mass Index (kg/m^2)	√	√	√
BMXLEG	Upper Leg Length (cm)	√		
BMXCALF	Maximal Calf Circumference (cm)	√		
BMXARML	Upper Arm Length (cm)	√		
BMXARMC	Arm Circumference (cm)	√		
BMXWAIST	Waist Circumference (cm)	√		√
BMXTHICR	Thigh Circumference (cm)	√		
BMXTRI	Triceps Skinfold (mm)	√		
DXXHEA	Head Area (cm^2)	√		
DXXHEBMC	Head Bone Mineral Content (grams)	√		
DXXHEBMD	Head Bone Mineral Density (grams/cm^2)	√		√
DXXHEFAT	Head Fat (grams)	√		
DXDHELE	Head Lean excluding Bone Mineral Content (grams)	√		
DXXHELI	Head Lean including Bone Mineral Content (grams)	√		
DXDHETOT	Head Total (grams)	√		
DXDHEPF	Head Percent Fat	√		√
DXXLAA	Left Arm Area (cm^2)	√		
DXXLABMC	Left Arm Body Mineral Content (grams)	√		
DXXLABMD	Left Arm Body Mineral Density (grams/cm^2)	√		√
DXXLAFAT	Left Arm Fat (grams)	√		
DXDLALE	Left Arm Lean excluding Body Mineral Content (grams)	√		
DXXLALI	Left Arm Lean including Bone Mineral Content (grams)	√		
DXDLATOT	Left Arm Total (grams)	√		
DXDLAPF	Left Arm Percent Fat	√		√
DXXLLA	Left Leg Area (cm^2)	√		
DXXLLBMC	Left Leg Bone Mineral Content (grams)	√		
DXXLLBMD	Left Leg Bone Mineral Density (grams/cm^2)	√		√
DXXLLFAT	Left Leg Fat (grams)	√		
DXDLLLE	Left Leg Lean excluding Bone Mineral Content (grams)	√		
DXXLLLI	Left Leg Lean including Bone Mineral Content (grams)	√		
DXDLLTOT	Left Leg Total (grams)	√		
DXDLLPF	Left Leg Percent Fat	√		√
DXXRAA	Right Arm Area (cm^2)	√		
DXXRABMC	Right Arm Bone Mineral Content (grams)	√		
DXXRABMD	Right Arm Bone Mineral Density (grams/cm^2)	√		√
DXXRAFAT	Right Arm Fat (grams)	√		
DXDRALE	Right Arm Lean excluding Bone Mineral Content (grams)	√		
DXXRALI	Right Arm Lean including Bone Mineral Content (grams)	√		
DXDRATOT	Right Arm Total (grams)	√		
DXDRAPF	Right Arm Percent Fat	√		√
DXXRLA	Right Leg Area (cm^2)	√		
DXXRLBMC	Right Leg Bone Mineral Content (grams)	√		
DXXRLBMD	Right Leg Bone Mineral Density (grams/cm^2)	√		√
DXXRLFAT	Right Leg Fat (grams)	√		
DXDRLLE	Right Leg Lean excluding Bone Mineral Content (grams)	√		
DXXRLLI	Right Leg Lean including Bone Mineral Content (grams)	√		
DXDRLTOT	Right Leg Total (grams)	√		
DXDRLPF	Right Leg Percent Fat	√		√
DXXLRA	Left Ribs Area (cm^2)	√		
DXXLRBMC	Left Ribs Bone Mineral Content (grams)	√		
DXXLRBMD	Left Ribs Bone Mineral Density (grams/cm^2)	√		√
DXXRRA	Right Ribs Area (cm^2)	√		
DXXRRBMC	Right Ribs Bone Mineral Content (grams)	√		
DXXRRBMD	Right Ribs Bone Mineral Density (grams/cm^2)	√		√
DXXTSA	Thoracic Spine Area (cm^2)	√		
DXXTSBMC	Thoracic Spine Bone Mineral Content (grams)	√		
DXXTSBMD	Thoracic Spine Bone Mineral Density (grams/cm^2)	√		√
DXXLSA	Lumbar Spine Area (cm^2)	√		
DXXLSBMC	Lumbar Spine Bone Mineral Content (grams)	√		
DXXLSBMD	Lumbar Spine Bone Mineral Density (grams/cm^2)	√		√
DXXPEA	Pelvis Area (cm^2)	√		
DXXPEBMC	Pelvis Bone Mineral Content (grams)	√		
DXXPEBMD	Pelvis Bone Mineral Density (grams/cm^2)	√		√
DXDTRA	Trunk Bone area (cm^2)	√		
DXDTRBMC	Trunk Bone Mineral Content (grams)	√		
DXDTRBMD	Trunk Bone Bone Mineral Density (g/cm^2)	√		
DXXTRFAT	Trunk Fat (grams)	√		

DXDTRLE	Trunk Lean excluding Bone Mineral Content (grams)	√		
DXXTRLI	Trunk Lean including Bone Mineral Content (grams)	√		
DXDTRTOT	Trunk Total (grams)	√		
DXDTRPF	Trunk Percent Fat	√		√
DXDSTA	Subtotal Area (cm^2)	√		
DXDSTBMC	Subtotal Bone Mineral Content (grams)	√		
DXDSTBMD	Subtotal Bone Mineral Density (grams/cm^2)	√		√
DXDSTFAT	Subtotal Fat (grams)	√		
DXDSTLE	Subtotal Lean excluding Bone Mineral Content (grams)	√		
DXDSTLI	Subtotal Lean including Bone Mineral Content (grams)	√		
DXDSTTOT	Subtotal (Total excluding Head) (grams)	√		
DXDSTPF	Subtotal Percent Fat	√		√
DXDTOA	Total Area (cm^2)	√		
DXDTOBMC	Total Bone Mineral Content (grams)	√		
DXDTOBMD	Total Bone Mineral Density (g/cm^2)	√		√
DXDTOFAT	Total Fat (grams)	√		
DXDTOLE	Total Lean excluding Bone Mineral Content (grams)	√		
DXDTOLI	Total Lean including Bone Mineral Content (grams)	√		
DXDTOTOT	Total Lean and Fat (grams)	√		
DXDTOPF	Total Percent Fat	√		√
LEALPN	Left foot number of insensate areas	√		
URXUMASI	Albumin urine (mg/L)	√		
URXUCRSI	Creatinine urine (μmol/L)	√		
LBDBPBSI	Lead (μmol/L)	√		
LBDBCDSI	Cadmium (nmol/L)	√		
LBDEPPSI	Protoporphyrin (μmol/L RBC)	√		
LBDIRNSI	Iron (μmol/L)	√	√	√
LBDTIBSI	Total iron binding capacity (μmol/L)	√	√	
LBXPCT	Transferrin saturation (%)	√	√	
LBDFERSI	Ferritin (μg/L)	√	√	
LBDFOLSI	Folate serum (nmol/L)	√	√	
LBDB12SI	Vitamin B12 serum (pmol/L)	√	√	
LBXHCY	Homocysteine (μmol/L)	√		
LBXMMA	Methylmalonic acid (μmol/L)	√		
LBDRBFSI	Folate RBC (nmol/L RBC)	√		
LBXCOT	Cotinine (ng/mL)	√	√	
LBDVIESI	Vitamin E (μmol/L)	√		
LBDTCSI	Total cholesterol (mmol/L)	√	√	√
LBDHDLSI	High-density lipoprotein (mmol/L)	√	√	√
LBXWBCSI	White blood cell count (1000 cells/μL)	√	√	√
LBXLYPCT	Lymphocyte percent	√	√	√
LBXMOPCT	Monocyte percent	√	√	√
LBXNEPCT	Segmented neutrophils percent	√	√	√
LBXEOPCT	Eosinophils percent	√	√	√
LBXBAPCT	Basophils percent	√	√	√
LBDLYMNO	Lymphocyte number (1000 cells/μL)	√	√	√
LBDMONO	Monocyte number (1000 cells/μL)	√	√	√
LBDNENO	Segmented neutrophils number (1000 cell/μL)	√	√	√
LBDEONO	Eosinophils number (1000 cells/μL)	√		√
LBDBANO	Basophils number (1000 cells/μL)	√	√	√
LBXRBCSI	Red blood cell count (million cells/μL)	√	√	√
LBXHGB	Hemoglobin (g/dL)	√	√	√
LBXHCT	Hematocrit (%)	√	√	√
LBXMCVSI	Mean cell volume (fL)	√	√	√
LBXMCHSI	Mean cell hemoglobin (pg)	√	√	√
LBXMC	Mean cell hemoglobin concentration (g/dL)	√	√	√
LBXRDW	Red cell distribution width (percent)	√	√	
LBXPLTSI	Platelet count (1000 cells/μL)	√	√	√
LBXMPSI	Mean platelet volume (fL)	√	√	
LBXCRP	C-reactive protein (mg/dL)	√	√	√
LBDFBSI	Fibrinogen (g/L)	√	√	
LBXBAP	Bone alkaline phosphotase (μg/L)	√		√
URXNT	N-telopeptides (nmol BCE)	√		
LBXGH	Glycohemoglobin (%)	√	√	
LBXHA	Hepatitis A antibody	√		
LBXHBC	Hepatitis B core antibody	√		
LBDHBG	Hepatitis B surface antigen	√		
LBDHCV	Hepatitis C antibody confirmed	√		
SSBNP	N-terminal pro-brain natriuretic peptide (pg/ml)	√	√	
LBDSALSI	Albumin (g/L)	√	√	√
LBXSATSI	Alanine aminotransferase (U/L)	√	√	√
LBXSASSI	Aspartate aminotransferase (U/L)	√	√	√
LBXSAPSI	Alkaline phosphatase (IU/L)	√	√	√
LBDSBUSI	Blood urea nitrogen (mmol/L)	√	√	√
LBDSCASI	Calcium total (mmol/L)	√	√	√
LBXSC3SI	Bicarbonate (mmol/L)	√	√	

LBXSGTSI	Gamma glutamyl transferase (U/L)	√	√	√
LBDSGLSI	Glucose (mmol/L)	√	√	√
LBXSLDSI	Lactate dehydrogenase (U/L)	√	√	
LBDSPHSI	Phosphorus (mmol/L)	√	√	
LBDSTBSI	Bilirubin total (μmol/L)	√	√	√
LBDSTPSI	Protein total (g/L)	√	√	√
LBDSTRSI	Triglycerides (mmol/L)	√	√	√
LBDSUASI	Uric acid (μmol/L)	√	√	√
LBDSCRSI	Creatinine (μmol/L)	√	√	√
LBXSNASI	Sodium (mmol/L)	√	√	√
LBXSKSI	Potassium (mmol/L)	√	√	√
LBXSCLSI	Chloride (mmol/L)	√	√	
LBXSOSSI	Osmolality (mOsm/Kg)	√		
LBDSGBSI	Globulin (g/L)	√	√	
N.A.¶	Co-morbidity index	√	√	√
N.A.¶	Self-health index	√	√	√
N.A.¶	Healthcare use index	√	√	√
N.A.¶	Low-density lipoprotein (mmol/L)		√	√
N.A.¶	Urine albumin-to-creatinine ratio (mg/g)		√	

N.A. = not applicable
¶ N.A. because these parameters were calculated / derived from a combination of variables in the NHANES 1999-2002 cohorts

Supplementary Table 2. Baseline characteristics of participants.

	NHANES 1999-2000 (training cohort)	NHANES 2001-2002 (testing cohort)
	***n* = 1,775**	***n* = 2,036**
Age (years) (mean ± SD)	59.86 ± 12.37	58.86 ± 12.56
Male sex (%)	52.00	53.73
Race (%)		
• Non-Hispanic White	47.14	57.73
• Non-Hispanic Black	16.63	17.57
• Mexican American	28.23	18.73
• Other Hispanic	5.53	3.52
• Other	2.47	2.45
Education (%)		
• < High school	43.65	29.95
• High school diploma	20.51	22.95
• > High school	35.65	47.01
• Missing	0.19	0.13
Poverty income ratio (mean ± SD)	2.64 ± 1.60	2.97 ± 1.61
• Missing (%)	14.68	7.28
Smoking (%)		
• Current	18.82	19.93
• No	34.09	33.89
• Missing	47.09	46.18
Alcohol (%)		
• Yes	59.51	60.95
• No	23.08	23.45
• Missing	17.41	15.59
Body mass index (kg/m^2) (mean ± SD)	28.66 ± 5.91	28.77 ± 6.04
Mortality status at 20-year follow-up (%)		
• Alive	55.38	64.95
• Deceased	44.57	35.01
• Missing	0.05	0.04

Supplementary Table 3. PC themes in PCAge.

PC	Themes
Chronological age	Not applicable
PC1	Sex
PC2	Body composition
PC3	Bone health
PC4	Cardiac function, renal function, inflammation and immunity, glucose regulation, iron storage and erythropoiesis
PC5	Uninterpretable
PC6	Uninterpretable
PC7	Hepatic function, renal function, immunity, iron storage and erythropoiesis
PC8	Uninterpretable
PC9	Uninterpretable
PC10	Nutrition, micronutrients
PC11	Body composition, glucose regulation, renal function
PC12	Uninterpretable
PC13	Body composition
PC14	Uninterpretable
PC15	Uninterpretable
PC16	Uninterpretable
PC17	Iron storage and erythropoiesis, glucose regulation, bone health
PC18	Inflammation

Supplementary Table 4. Cluster centers for each PC utilized in clustering.

Male	Cluster 1: Major Cardio-metabolic (n = 209)	Cluster 2: Multi-morbid (n = 382)	Cluster 3: Cardio-metabolic Failure (n = 49)	Cluster 4: Healthy Aging (n = 753)	Cluster 5: Mild Cardio-metabolic (n = 624)
PC2	3.69 ± 2.84	-5.80 ± 2.15	3.22 ± 7.41	-3.29 ± 1.33	0.50 ± 1.42
PC3	-2.23 ± 2.38	0.40 ± 2.66	-1.14 ± 3.91	-0.55 ± 1.73	-1.61 ± 2.01
PC4	0.51 ± 2.92	0.89 ± 2.38	3.59 ± 4.46	-0.45 ± 1.48	-0.24 ± 1.86
PC7	0.48 ± 2.33	-0.09 ± 2.07	1.86 ± 5.09	-0.19 ± 1.32	-0.12 ± 1.54
PC10	0.24 ± 1.92	-0.78 ± 1.81	-0.30 ± 2.47	0.09 ± 1.35	0.18 ± 1.48
PC11	-0.31 ± 1.92	-0.20 ± 1.93	-0.96 ± 3.72	0.33 ± 1.34	0.17 ± 1.43
PC13	0.04 ± 1.82	-0.25 ± 1.82	0.28 ± 1.26	0.23 ± 1.24	0.19 ± 1.30
PC17	0.10 ± 1.58	0.12 ± 1.67	0.62 ± 1.84	-0.12 ± 1.13	-0.14 ± 1.24
PC18	-0.26 ± 1.63	0.15 ± 1.47	0.00 ± 3.59	0.19 ± 1.00	0.00 ± 1.18
**Female**	**Cluster 1: Multi-morbid (n = 282)**	**Cluster 2: Mild Cardio-metabolic (n = 627)**	**Cluster 3: Cardio-metabolic Failure (n = 74)**	**Cluster 4: Healthy Aging (n = 476)**	**Cluster 5: Major Cardio-metabolic (n = 335)**
PC2	-3.42 ± 2.16	2.88 ± 1.39	9.62 ± 5.11	-0.58 ± 1.31	6.47 ± 1.99
PC3	1.60 ± 3.47	0.43 ± 2.06	-0.15 ± 3.62	2.08 ± 2.06	0.26 ± 2.74
PC4	0.20 ± 2.29	-0.28 ± 1.59	1.68 ± 3.69	-0.49 ± 1.53	0.29 ± 2.16
PC7	0.18 ± 1.90	-0.09 ± 1.42	0.69 ± 4.29	0.09 ± 1.49	-0.07 ± 1.91
PC10	0.12 ± 1.91	0.12 ± 1.32	-0.35 ± 2.84	0.25 ± 1.38	-0.37 ± 1.61
PC11	-0.20 ± 1.48	-0.07 ± 1.26	-0.19 ± 2.52	-0.04 ± 1.40	-0.12 ± 1.56
PC13	-0.24 ± 1.42	-0.16 ± 1.20	-0.01 ± 2.34	-0.15 ± 1.19	0.06 ± 1.57
PC17	-0.07 ± 1.52	0.08 ± 1.17	0.41 ± 1.74	-0.02 ± 1.12	0.09 ± 1.40
PC18	-0.32 ± 1.27	-0.01 ± 1.01	-0.07 ± 2.47	-0.07 ± 1.00	-0.06 ± 1.33
Data are shown as Mean ± SD					

Supplementary Table 5. Characteristics of male and female participants from each cluster.

Male Characteristic	Cluster 1: Major Cardio-metabolic (n = 209)	Cluster 2: Multi-morbid (n = 382)	Cluster 3: Cardio-metabolic Failure (n = 49)	Cluster 4: Healthy Aging (n = 753)	Cluster 5: Mild Cardio-metabolic (n = 624)	P-Value
Chronological Age (years) (IQR)	61.3 (51.3 – 70.7)	60.2 (48.4 – 70.9)	60.3 (51.5 – 67.4)	58.8 (47.9 – 69.1)	60.5 (50.0 – 70.4)	N.S.
PCAge (years) (IQR)	63.2 (54.0 – 76.1)	60.6 (48.1 – 73.4)	73.4 (51.9 – 87.4)	54.5 (43.6 – 64.9)	59.2 (47.6 – 69.4)	< 0.001* (post-tests p < 0.05 across all clusters, except p = 0.06 between Clusters 1 & 3, 2 & 5)
PCAge Delta (years) (IQR)	2.1 (-2.5 – 7.6)	0.1 (-5.5 – 6.3)	10.3 (-0.3 – 22.3)	-4.6 (-7.4 – -1.2)	-1.8 (-5.9 – 2.2)	< 0.001* (post-tests p < 0.01 across all clusters)
Education level (%)						
< High school	36.8	43.5	38.8	31.6	37.0	< 0.001 for Clusters 2 (more than expected) and 4 (fewer than expected)[#]
High school diploma	18.2	19.4	16.3	21.9	20.7	N.S. for all clusters[#]
> High school	45.0	37.2	44.9	46.3	42.1	< 0.01 for Cluster 2 (fewer than expected)[#] and 0.01 for Cluster 4 (more than expected)[#]
Missing	0	0	0	0.1	0.2	N.S. for all clusters[#]
Poverty income ratio	2.6 (1.3 – 4.6)	2.1 (1.2 – 4.2)	1.9 (1.1 – 4.1)	3.0 (1.6 – 5.0)	3.0 (1.5 – 4.9)	< 0.001* (post-tests p < 0.001 between Clusters 2 & 4, 2 & 5; p < 0.05 between Clusters 3 & 4, 3 & 5)
Missing (%)	7.7	8.9	6.1	7.8	10.4	
Smoking (%)						
Current	22.0	39.0	24.5	19.7	16.0	< 0.001 for Clusters 2 (more than expected) and 5 (fewer than expected)[#] ; < 0.01 for Cluster 4 (fewer than expected)[#]
No	50.7	31.4	42.9	40.0	50.8	< 0.001 for Clusters 2 (fewer than expected) and 5 (more than expected)[#] ; < 0.01 for Cluster 1 (more than expected)[#] ; 0.02 for Cluster 4 (fewer than expected)[#]
Missing	27.3	29.6	32.7	40.4	33.2	< 0.001 for Cluster 4 (more than expected)[#] ; 0.01 for Clusters 1 (fewer than expected) and 2 (fewer than expected)[#]
≥ 5 alcohol drinks/day (%)						
Yes	26.3	30.1	26.5	22.3	26.6	< 0.01 for Cluster 4 (fewer than expected)[#] ; 0.01 for Cluster 2 (more than expected)[#]
No	66.0	62.8	67.3	70.5	66.8	< 0.01 for Cluster 4 (more than expected)[#] ; 0.02 for Cluster 2 (fewer than expected)[#]
Missing	7.7	7.1	6.1	7.2	6.6	N.S. for all clusters[#]
Body mass index (BMI) (kg/m^2) (IQR)	34.2 (31.3 – 37.5)	23.2 (21.2 – 25.1)	31.9 (25.4 – 42.5)	26.3 (24.7 – 28.0)	30.2 (28.8 – 32.0)	< 0.001* (post-tests p < 0.001 between Clusters 1 & 2, 1 & 4, 1 & 5, 2 & 3, 2 & 4, 2 & 5, 3 & 4, 4 & 5)
Clinical frailty score (CFS) (age ≥ 65) (IQR)	4 (4 – 6)	4 (3 – 6)	5 (4 – 6)	4 (2 – 5)	4 (3 – 6)	< 0.001* (post-tests p < 0.001 between Clusters 1 & 4, 4 & 5; p < 0.01 between Clusters 3 & 4; p = 0.02 between Clusters 2 & 4
Missing (%)	2.5	11.0	5.3	15.9	12.4	N.A.
Diseases (%)						
None	4.3	31.2	4.1	33.1	17.0	< 0.001 for Clusters 1 (fewer than expected), 2 (more than expected), 3 (fewer than expected), 4 (more than expected) and 5 (fewer than expected)[#]
Cardiovascular disease[##]	64.6	40.3	79.6	39.0	55.3	< 0.001 for Cluster 1 (more than expected), 2 (fewer than expected), 3 (more than expected), 4 (fewer than expected) and 5 (more than expected)[#]

Cancer	3.3	4.5	4.1	3.6	4.3	N.S. for all clusters[#]
Kidney disease	0	0	0	0.7	0.5	0.03 for Cluster 4 (more than expected)[#]
Liver disease	1.9	2.1	2.0	1.5	0.8	N.S. for all clusters[#]
Asthma	3.8	2.9	0	3.6	2.7	N.S. for all clusters[#]
Chronic obstructive pulmonary disease	1.0	2.4	0	1.5	0.8	0.02 for Cluster 2 (more than expected)[#]
Arthritis	7.7	10.2	4.1	9.0	8.0	N.S. for all clusters[#]
Anemia	0	0	2.0	0.1	0.2	< 0.01 for Cluster 3 (more than expected)[#]
Thyroid disease	0	0.3	0	0.1	0.2	N.S. for all clusters[#]
Obesity	11.5	0.3	2.0	3.2	7.1	< 0.001 for Clusters 1 (more than expected), 2 (fewer than expected) and 5 (more than expected)[#] ; < 0.01 for Cluster 4 (fewer than expected)[#]
Osteoporosis and fragility (hip, wrist, spine) fractures	1.0	3.4	2.0	4.1	2.2	0.01 for Cluster 4 (more than expected)[#] ; 0.04 for Cluster 1 (fewer than expected)[#]
Cognitive impairment	1.0	2.6	0	0.5	1.0	< 0.001 for Cluster 2 (more than expected)[#]
Mortality (%)						
Alive	43.5	46.1	32.7	66.4	57.5	< 0.001 for Clusters 1 (fewer than expected), 2 (fewer than expected), 3 (fewer than expected) and 4 (more than expected)[#]
Cardiovascular disease[##]	24.4	18.3	34.7	12.4	14.7	< 0.001 for Clusters 1 (more than expected), 2 (more than expected) and 4 (fewer than expected)[#]
Cancer	14.4	14.9	12.2	8.9	10.6	< 0.01 for Clusters 2 (more than expected) and 4 (fewer than expected)[#]
Kidney disease	3.3	0.3	0	0.8	1.1	< 0.001 for Cluster 1 (more than expected)[#]
Chronic lower respiratory disease	1.9	3.9	2.0	2.1	1.9	0.01 for Cluster 2 (more than expected) [#]
Influenza and Pneumonia	0	1.8	2.0	0.3	1.0	< 0.01 for Cluster 2 (more than expected)[#] ; 0.03 for Cluster 4 (fewer than expected)[#]
Alzheimer's dementia	0	2.9	0	0.8	1.8	< 0.01 for Cluster 2 (more than expected)[#]
All other causes of death	12.4	11.8	16.3	8.4	11.4	< 0.01 for Cluster 4 (fewer than expected)[#]
Able to do tasks around home or yard over past 30 days (%)						
Yes	57.4	56.0	53.1	68.0	64.1	< 0.001 for Cluster 4 (more than expected)[#] ; < 0.01 for Cluster 2 (fewer than expected)[#] ; 0.04 for Cluster 1 (fewer than expected)[#]
No	41.6	41.1	38.8	31.6	34.1	< 0.01 for Clusters 2 (more than expected) and 4 (fewer than expected)[#] ; 0.02 for Cluster 1 (more than expected)[#]
Unable	1.0	2.6	8.2	0.3	1.8	< 0.001 for Clusters 3 (more than expected) and 4 (fewer than expected)[#] ; 0.01 for Cluster 2 (more than expected)[#]
Missing	0	0.3	0	0.1	0	0.04 for Cluster 2 (more than expected)[#]
Average level of daily physical activity (%)						
Sedentary	34.4	20.2	46.9	15.9	25.3	< 0.001 for Clusters 1 (more than expected), 3 (more than expected) and 4 (fewer than expected)[#] ; 0.01 for Cluster 5 (more than expected)[#]
Stand / Walk a lot	47.4	55.8	44.9	55.8	52.6	0.03 for Cluster 1 (fewer than expected)[#]
Climb stairs / Carry light loads	10.5	16.5	6.1	18.6	13.9	< 0.01 for Cluster 4 (more than expected)[#] ; 0.02 for Cluster 1 (fewer than expected)[#] ; 0.04 for Cluster 3 (fewer than expected)[#]

Does heavy work / Carry heavy loads	7.2	7.6	2.0	9.6	7.9	0.04 for Cluster 4 (more than expected)[#]
Missing	0.5	0	0	0.1	0.3	N.S. for all clusters[#]
Does muscle strengthening activities (%)						
Yes	13.4	22.5	18.4	24.6	19.6	< 0.01 for Clusters 1 (fewer than expected) and 4 (more than expected)[#]
No	84.2	74.6	77.6	74.6	77.1	< 0.01 for Cluster 1 (more than expected)[#]
Unable	2.4	2.9	4.1	0.8	3.4	< 0.001 for Cluster 4 (fewer than expected)[#] ; < 0.01 for Cluster 5 (more than expected)[#]
Missing	0	0	0	0	0	N.A.
Number of times received healthcare over past year (%)						
0	8.6	24.6	12.2	20.7	14.9	< 0.001 for Clusters 1 (fewer than expected) and 2 (more than expected)[#] ; < 0.01 for Cluster 5 (fewer than expected)[#] ; 0.01 for Cluster 4 (more than expected)[#]
1	10.0	16.2	6.1	20.7	18.8	< 0.001 for Cluster 1 (fewer than expected)[#] ; < 0.01 for Cluster 4 (more than expected)[#] ; 0.02 for Cluster 3 (fewer than expected)[#]
2-3	25.8	25.9	18.4	26.8	28.5	N.S. for all clusters[#]
4-9	34.0	21.5	28.6	24.0	25.3	< 0.001 for Cluster 1 (more than expected)[#] ; 0.04 for Cluster 2 (fewer than expected)[#]
10-12	10.5	5.2	10.2	4.4	6.4	< 0.01 for Cluster 1 (more than expected)[#] ; 0.01 for Cluster 4 (fewer than expected)[#]
≥ 13	11.0	6.3	24.5	3.3	6.1	< 0.01 for Clusters 1 (more than expected), 3 (more than expected) and 4 (fewer than expected)[#]
Missing	0	0.3	0	0	0	< 0.001 for Cluster 2 (more than expected)[#]
Required hospitalization over past year (%)						
Yes	17.7	14.1	24.5	6.1	12.8	< 0.001 for Clusters 3 (more than expected) and 4 (fewer than expected)[#] ; < 0.01 for Cluster 1 (more than expected)[#] ; 0.03 for Cluster 2 (more than expected)[#]
No	81.8	85.9	75.5	93.9	87.2	< 0.001 for Cluster 4 (more than expected)[#] ; < 0.01 for Clusters 1 (fewer than expected) and 3 (fewer than expected)[#] ; 0.04 for Cluster 2 (fewer than expected)[#]
Missing	0.5	0	0	0	0	< 0.001 for Cluster 1 (more than expected)[#]
ACE-I or ARB treatment (45-64 year olds)						
All subjects who need ACE-I or ARB treatment	41 (41.4%)	27 (15.7%)	7 (33.3%)	64 (18.2%)	66 (22.9%)	Cluster 1: < 0.001 for more subjects than expected needing ACE-I or ARB[#] ; Cluster 2: 0.02 for fewer subjects than expected needing ACE-I or ARB[#] ; Cluster 3: N.S.[#] ; Cluster 4: 0.02 for fewer subjects than expected needing ACE-I or ARB[#] ; Cluster 5: N.S.[#]
Subjects treated with ACE-I or ARB	30 (30.3%)	13 (7.6%)	3 (14.3%)	50 (14.2%)	44 (15.3%)	Cluster 1: N.S.[#] ; Cluster 2: 0.03 for fewer subjects than expected treated with ACE-I or ARB[#] ; Cluster 3: N.S.[#] ; Cluster 4: 0.01 for more subjects than expected treated with ACE-I or ARB[#] ; Cluster 5: N.S.[#]
Missed subjects who need ACE-I or ARB treatment	11 (11.1%)	14 (8.1%)	4 (19%)	14 (4%)	22 (7.6%)	Cluster 1: N.S.[#] ; Cluster 2: < 0.01 for more missed subjects than expected who need ACE-I or ARB[#] ; Cluster 3: 0.04 for more missed subjects than expected who need ACE-I or ARB[#] ; Cluster 4: 0.03 for fewer missed subjects than expected who need ACE-I or ARB[#] ; Cluster 5: N.S.[#]
Centenarians	1	1	0	2	2	N.S. across all clusters[#]

Female Characteristic	Cluster 1: Multi-morbid (n = 282)	Cluster 2: Mild Cardio-metabolic (n = 627)	Cluster 3: Cardio-metabolic Failure (n = 74)	Cluster 4: Healthy Aging (n = 476)	Cluster 5: Major Cardio-metabolic (n = 335)	P-Value
Chronological Age (years) (IQR)	55.4 (47.1 – 71.3)	62.7 (51.5 – 71.4)	58.3 (46.8 – 65.0)	56.8 (47.2 – 66.8)	61.4 (50.9 – 70.5)	< 0.001* (post-tests p < 0.001 between Clusters 2 & 4, 4 & 5; p < 0.01 between Clusters 1 & 2, 2 & 3)
PCAge (years) (IQR)	55.7 (45.0 – 73.7)	60.2 (49.6 – 70.8)	57.5 (48.7 – 71.7)	53.0 (44.1 – 63.8)	60.2 (49.8 – 72.3)	< 0.001* (post-tests p < 0.001 between Clusters 2 & 4, 4 & 5; p < 0.01 between Clusters 1 & 4, 3 & 4)
PCAge Delta (years) (IQR)	-0.8 (-5.0 – 4.3)	-2.5 (-5.5 – 1.6)	4.6 (-3.8 – 10.3)	-3.4 (-6.4 – -0.6)	-0.6 (-4.3 – 4.2)	< 0.001* (post-tests p < 0.001 between Clusters 1 & 2, 1 & 4, 2 & 3, 2 & 4, 2 & 5, 3 & 4, 4 & 5; p < 0.01 between Clusters 1 & 3, 3 & 5)
Education level (%)						
< High school	27.3	39.7	37.8	27.3	41.5	< 0.001 for Clusters 2 (more than expected) and 4 (fewer than expected)[#] ; < 0.01 for Clusters 1 (fewer than expected) and 5 (more than expected)[#]
High school diploma	20.6	24.7	23.0	23.5	23.6	N.S. for all clusters[#]
> High school	52.1	35.2	39.2	48.9	34.6	< 0.001 for Clusters 1 (more than expected), 2 (fewer than expected) and 4 (more than expected)[#] ; < 0.01 for Cluster 5 (fewer than expected)[#]
Missing	0	0.3	0	0.2	0.3	N.S. for all clusters
Poverty income ratio	2.9 (1.4 – 5.0)	2.4 (1.3 – 4.4)	2.0 (1.1 – 3.4)	3.1 (1.7 – 5.0)	2.2 (1.2 – 4.0)	< 0.001* (post-tests p < 0.001 between Clusters 2 & 4, 3 & 4, 4 & 5; p < 0.01 between Clusters 1 & 3, 1 & 5; p < 0.05 between Clusters 1 & 2)
Missing (%)	10.6	11.2	9.5	9.5	9.9	
Smoking (%)						
Current	27.0	15.9	17.6	14.5	13.1	< 0.001 for Cluster 1 (more than expected)[#] and 0.02 for Cluster 5 (fewer than expected)[#]
No	25.5	22.8	29.7	26.3	25.7	N.S. for all clusters[#]
Missing	47.5	61.2	52.7	59.2	61.2	< 0.001 for Cluster 1 (fewer than expected)[#] and 0.02 for Cluster 2 (more than expected)[#]
≥ 5 alcohol drinks/day (%)						
Yes	8.2	4.6	2.7	5.7	5.4	0.02 for Cluster 1 (more than expected)[#]
No	77.7	70.5	66.2	73.5	62.7	< 0.01 for Clusters 1 (more than expected) and 5 (fewer than expected)[#]
Missing	14.2	24.9	31.1	20.8	31.9	< 0.001 for Clusters 1 (fewer than expected) and 5 (more than expected)[#]
Body mass index (BMI) (kg/m^2) (IQR)	21.1 (19.7 – 23.1)	28.9 (26.9 – 30.7)	40.3 (35.5 – 43.8)	24.6 (23.2 – 26.2)	34.0 (31.9 – 36.4)	< 0.001* (post-tests p < 0.001 between all clusters)
Clinical frailty score (CFS) (age ≥ 65) (IQR)	4 (3 – 6)	5 (4 – 6)	6 (5.25 – 6.75)	4 (3 – 5)	5 (4 – 6)	< 0.001* (post-tests p < 0.001 between Clusters 1 & 3, 1 & 5, 2 & 3, 2 & 5, 3 & 4, 4 & 5; p = 0.02 between Clusters 3 & 5)
Missing (%)	7.1	9.0	5.3	17.5	0.8	N.A.
Diseases (%)						
None	27.3	12.9	6.8	28.8	5.1	< 0.001 for Clusters 1 (more than expected), 2 (fewer than expected), 4 (more than expected) and 5 (fewer than expected)[#] ; < 0.01 for Cluster 3 (fewer than expected)[#]
Cardiovascular disease[##]	35.1	52.5	71.6	35.7	63.3	< 0.001 for Clusters 1 (fewer than expected), 3 (more than expected), 4 (fewer than expected) and 5 (more than expected)[#] ; < 0.01 for Cluster 2 (more than expected)[#]

Cancer	5.7	4.1	2.7	7.1	3.3	< 0.01 for Cluster 4 (more than expected)#
Kidney disease	1.1	0.5	0	0.8	0.9	N.S. for all clusters#
Liver disease	2.8	0.6	0	1.3	1.2	< 0.01 for Cluster 1 (more than expected)#
Asthma	3.9	4.5	2.7	4.0	2.4	N.S. for all clusters#
Chronic obstructive pulmonary disease	3.9	1.9	1.4	1.3	1.2	< 0.01 for Cluster 1 (more than expected)#
Arthritis	9.9	12.9	6.8	9.2	9.6	< 0.01 for Cluster 2 (more than expected)#
Anemia	1.8	0.3	1.4	3.2	0.6	0.02 for Cluster 1 (more than expected)#
Thyroid disease	0.7	1.3	0	1.1	0.9	N.S. for all clusters#
Obesity	1.1	6.5	6.8	3.2	10.7	< 0.001 for Clusters 1 (fewer than expected) and 5 (more than expected)# ; < 0.01 for Cluster 4 (fewer than expected)#
Osteoporosis and fragility (hip, wrist, spine) fractures	5.3	1.4	0	5.7	0.9	< 0.001 for Cluster 4 (more than expected)# ; < 0.01 for Clusters 1 (more than expected), 2 (fewer than expected) and 5 (fewer than expected)#
Cognitive impairment	1.4	0.5	0	0.8	0	0.02 for Cluster 1 (more than expected)#
Mortality (%)						
Alive	61.3	63.5	58.1	74.2	57.0	< 0.001 for Clusters 4 (more than expected) and 5 (fewer than expected)#
Cardiovascular disease##	11.0	11.0	20.3	8.4	17.0	< 0.001 for Cluster 5 (more than expected)# ; < 0.01 for Cluster 4 (fewer than expected)# ; 0.01 for Cluster 3 (more than expected)#
Cancer	6.0	7.7	4.1	5.5	11.3	< 0.01 for Cluster 5 (more than expected)# and 0.04 for Cluster 4 (fewer than expected)#
Kidney disease	0.7	1.1	2.7	0	0.6	0.01 for Cluster 3 (more than expected)# and 0.02 for Cluster 4 (fewer than expected)#
Chronic lower respiratory disease	5.3	2.2	1.4	1.9	1.8	< 0.001 for Cluster 1 (more than expected)#
Influenza and Pneumonia	1.8	0.3	4.1	0.2	0.3	< 0.01 for Clusters 1 (more than expected) and 3 (more than expected)#
Alzheimer's dementia	3.2	2.4	1.4	1.3	1.2	0.04 for Cluster 1 (more than expected)#
All other causes of death	10.6	11.8	8.1	8.6	10.7	N.S. for all clusters#
Able to do tasks around home or yard over past 30 days (%)						
Yes	53.5	51.0	41.9	56.9	44.8	< 0.01 for Clusters 4 (more than expected) and 5 (fewer than expected)#
No	44.7	45.9	48.6	41.0	50.4	0.01 for Cluster 4 (fewer than expected)# ; 0.02 for Cluster 5 (more than expected)#
Unable	1.8	2.7	9.5	1.9	4.8	< 0.01 for Cluster 3 (more than expected)# ; 0.02 for Cluster 5 (more than expected)#
Missing	0	0.3	0	0.2	0	0.04 for Cluster 2 (more than expected)#
Average level of daily physical activity (%)						
Sedentary	21.3	26.2	43.2	17.6	31.6	< 0.001 for Clusters 3 (more than expected), 4 (fewer than expected) and 5 (more than expected)#
Stand / Walk a lot	59.9	60.6	40.5	64.1	58.5	< 0.001 for Cluster 3 (fewer than expected)# ; 0.02 for Cluster 4 (more than expected)#
Climb stairs / Carry light loads	15.6	11.6	13.5	15.1	9.6	0.02 for Cluster 5 (fewer than expected)# ; 0.04 for Cluster 4 (more than expected)#

Does heavy work / Carry heavy loads	3.2	1.1	2.7	2.9	0.3	< 0.01 for Cluster 5 (fewer than expected)[#] ; 0.01 for Cluster 4 (more than expected)[#] ; 0.03 for Cluster 1 (more than expected)[#]
Missing	0	0.5	0	0	0	0.01 for Cluster 2 (more than expected)[#]
Does muscle strengthening activities (%)						
Yes	22.7	13.2	8.1	24.4	10.7	< 0.001 for Clusters 4 (more than expected) and 5 (fewer than expected)[#] ; < 0.01 for Clusters 1 (more than expected) and 2 (fewer than expected)[#] ; 0.02 for Cluster 3 (fewer than expected)[#]
No	74.1	83.4	77.0	73.1	84.2	< 0.001 for Clusters 2 (more than expected) and 4 (fewer than expected)[#] ; < 0.01 for Cluster 5 (more than expected)[#] ; 0.02 for Cluster 1 (fewer than expected)[#]
Unable	3.2	3.0	14.9	2.3	5.1	< 0.001 for Cluster 3 (more than expected)[#] ; 0.03 for Cluster 4 (fewer than expected)[#]
Missing	0	0.3	0	0.2	0	0.04 for Cluster 2 (more than expected)[#]
Number of times received healthcare over past year (%)						
0	8.2	7.8	9.5	8.4	9.6	N.S. for all clusters[#]
1	17.0	16.1	10.8	21.2	12.5	< 0.01 for Cluster 4 (more than expected)[#] ; 0.01 for Cluster 5 (fewer than expected)[#]
2-3	29.8	28.1	24.3	33.2	25.1	< 0.01 for Cluster 4 (more than expected)[#]
4-9	28.0	32.9	25.7	24.6	33.7	< 0.01 for Cluster 4 (fewer than expected)[#] ; 0.03 for Cluster 5 (more than expected)[#] ; 0.02 for Cluster 2 (more than expected)[#]
10-12	6.7	7.8	10.8	4.8	10.1	< 0.01 for Cluster 4 (fewer than expected)[#] ; 0.02 for Cluster 5 (more than expected)[#]
≥ 13	10.3	7.2	18.9	7.6	9.0	< 0.01 for Cluster 3 (more than expected)[#]
Missing	0	0.2	0	0.2	0	N.S. for all clusters[#]
Required hospitalization over past year (%)						
Yes	13.8	10.2	20.3	8.6	16.1	< 0.01 for Clusters 4 (fewer than expected) and 5 (more than expected)[#] ; 0.01 for Cluster 3 (more than expected)[#]
No	86.2	89.8	79.7	91.4	83.9	< 0.01 for Clusters 4 (more than expected) and 5 (fewer than expected)[#] ; 0.02 for Cluster 3 (fewer than expected)[#]
Missing	0	0	0	0	0	N.A.
ACE-I or ARB treatment (45-64 year olds)						
All subjects who need ACE-I or ARB treatment	21 (16.9%)	61 (22.7%)	17 (42.5%)	32 (13.5%)	58 (34.9%)	Cluster 1: N.S.[#] ; Cluster 2: N.S.[#] ; Cluster 3: 0.001 for more subjects than expected needing ACE-I or ARB[#] ; Cluster 4: < 0.001 for fewer subjects than expected needing ACE-I or ARB[#] ; Cluster 5: < 0.001 for more subjects than expected needing ACE-I or ARB[#]
Subjects treated with ACE-I or ARB	9 (7.3%)	41 (15.2%)	12 (30%)	19 (8%)	44 (26.5%)	Cluster 1: 0.02 for fewer subjects than expected treated with ACE-I or ARB[#] ; Clusters 2-5: N.S.[#]
Missed subjects who need ACE-I or ARB treatment	12 (9.7%)	20 (7.4%)	5 (12.5%)	13 (5.5%)	14 (8.4%)	Cluster 1: < 0.01 for more missed subjects than expected who need ACE-I or ARB[#] ; Clusters 2-5: N.S.[#]
Centenarians	0	3	0	4	1	0.03 for Cluster 4 (more than expected)[#]

IQR = interquartile range; N.S. = not significant; N.A. = not applicable
¶ This parameter was presented as a fold change versus expected for age, which was obtained by determining the residuals from a linear regression analysis of each parameter against chronological age.
Continuous data are presented as median (25th and 75th percentiles). Categorical variables are presented as percentage (%).
* This value was based on a Kruskal-Wallis test across all clusters. Post-test pairwise comparisons using Wilcoxon rank sum test with continuity correction were also performed between clusters.
[#] This value was based on a hypergeometric probability distribution.

Cardiovascular disease includes heart failure, coronary heart disease, angina, acute myocardial infarction, stroke, hypertension, and diabetes mellitus.

Supplementary Note 1

When the "healthy aging" cluster was compared to all other clusters, after 20 years of follow-up, there were significantly more "healthy agers" who remained alive (*P*<0.001 for both), and, overall, significantly fewer deaths, especially due to cardiovascular disease (*P*<0.001 for males and *P*<0.01 for females) and cancer (*P*<0.01 for males and *P*=0.04 for females). Those aged 65 and above also remained significantly less frail (*P*<0.001 for both). "Healthy agers" were significantly more highly educated (*P*=0.01 for males and *P*<0.001 for females), wealthier, with higher median poverty income ratios (*P*<0.001 for both), and males were less likely to smoke (*P*<0.01) and abuse alcohol (*P*<0.01) although the latter were not statistically significant for females. Compared to their peers, "healthy agers" were also significantly more physically active (*P*<0.05 for both) and more likely to participate in muscle strengthening activities (*P*<0.01 for males and *P*<0.001 for females). It is important to note that, none of these socioeconomic, lifestyle or exercise data were part of the data used to construct the PCA and that PCAge calculation and clustering was performed solely on clinical parameters. Healthy agers had lower median BMI (*P*<0.001 for both) and were better able to perform functional tasks around the home (*P*<0.001 for males and *P*<0.01 for females). Overall, "healthy agers" appeared to have fewer chronic diseases compared to members from the other clusters and there were significantly more "healthy agers" who were disease-free (*P*<0.001 for both). As expected, there were significantly fewer "healthy agers" who needed to take chronic medications, for example, an ACE-I or ARB (*P*=0.02 for males and *P*<0.001 for females). When treatment was medically indicated, male "healthy agers" tended to be started on a chronic medication, for example, an ACE-I or ARB, at an earlier age (*P*=0.01 for 45-64 yo males) compared to members of other clusters and there were significantly fewer male (but not female) "healthy agers" who were missed, that is, who met medical indication for prescriptions of ACE-I or ARB but for which no such treatment had been initiated (*P*=0.03 for 45-64 yo males). These data suggest that one determinant of membership in the "healthy aging" cluster may be good access to timely medical care. Indeed, despite being generally healthier, "healthy agers" tended to visit their healthcare providers more often than members of other clusters 1-3 times per year (*P*<0.001 for males who had 1 healthcare visit, and *P*<0.01 for females who had 1-3 healthcare visits, over the past year). Unsurprisingly then, when compared to the other clusters, "healthy agers" had significantly fewer hospitalizations over the past year (*P*<0.001 for males and *P*<0.01 for females who did not require hospitalization over the past year).

Along the cardio-metabolic axis, there appeared to be a trend towards a progressive decline in median poverty income ratios although this was not statistically significant between clusters. Females from the "cardio-metabolic" clusters tended to receive less education (*P*<0.001 for "mild cardio-metabolic" and *P*<0.01 for "major cardio-metabolic"), although this was not the case for males. There were significantly fewer current smokers in the male "mild cardio-metabolic" cluster (*P*<0.001), more non-smokers in the male "major cardio-metabolic" cluster (*P*<0.01), and fewer current smokers in the female "major cardio-metabolic" cluster (*P*=0.02). Compared to the other clusters, alcohol use disorder was not significantly higher among members from the "cardio-metabolic" clusters. Along the cardio-metabolic axis, members of the "cardio-metabolic" clusters became increasingly sedentary (*P*=0.01 for "mild" males, *P*<0.001 for "major" males and females), had progressively higher median BMI (*P*<0.001 for both), were less likely and less able to participate in muscle strengthening activities (*P*<0.01 for both), and members from the "major cardio-metabolic" clusters were less able to perform functional tasks around the home (*P*=0.02 for both). When compared to "healthy agers", "cardio-metabolic" members aged 65 and above also became increasingly frailer (*P*<0.001 for both) along the cardio-metabolic axis. As expected, members from the "cardio-metabolic" clusters were significantly less healthy (*P*<0.001 for no diseases for all members), and suffered mainly from cardiovascular disease (*P*<0.001 for all males, *P*<0.01 for "mild" females, and *P*<0.001 for "major" females) and obesity (*P*<0.001 for all males, *P*<0.001 for "major" females, and not statistically significant for "mild" females), with significantly higher disease rates seen in the "major" compared to the "minor" clusters. In addition, a significant proportion of females from the "mild cardio-metabolic" cluster suffered from arthritis (*P*<0.01). Members from the "major cardio-metabolic" clusters had significantly higher healthcare utilization (*P*<0.01 for males and *P*=0.03 for females for at least 4 healthcare visits over the past year) and hospitalizations (*P*<0.01 for both). After 20 years of follow-up, there were fewer members from the "major cardio-metabolic" clusters who were still alive (43.5% for males and 57% for females, *P*<0.001 for both), and most deaths were due to cardiovascular disease (24.4% for males and 17% for females, *P*<0.001 for both), although 3.3% of

males also succumbed to kidney disease (*P*<0.001) and 11.3% of females succumbed to cancer (*P*<0.01). There were no significant differences for members from the "mild cardio-metabolic" clusters in terms of overall survival and disease-specific mortality.

An overwhelming proportion of members from the "cardio-metabolic failure" clusters suffered from cardiovascular disease (79.6% for males and 71.6% for females, *P*<0.001 for both), and they had the highest healthcare utilization (*P*<0.01 for males and females who required at least 13 healthcare visits over the past year) and hospitalizations (*P*<0.001 for males and *P*=0.01 for females) amongst all clusters. After 20 years of follow-up, the "cardio-metabolic failure" clusters had the fewest members who were still alive (32.7% for males and 58.1% for females, *P*<0.001 for males although not significant for females), and most deaths within these clusters were due to cardiovascular disease (34.7% for males and 20.3% for females, *P*=0.01 for females although not significant for males). While they had significantly lower median poverty income ratios (*P*<0.05 for males and *P*<0.001 for females) compared to "healthy agers", members of the "cardio-metabolic failure" clusters did not differ from the other clusters in terms of education level, smoking and alcohol use disorder. Compared to "healthy agers", members of the "cardio-metabolic failure" clusters were significantly more sedentary (*P*<0.001 for both), had higher median BMI (*P*<0.001 for both), with many more members who were unable to perform functional tasks around the home (*P*<0.001 for males and *P*<0.01 for females), and those aged 65 and above were significantly frailer (*P*<0.01 for males and *P*<0.001 for females).

While the "healthy aging" and "cardio-metabolic" clusters were essentially the same for males (Fig. 3a) and females (Fig. 3b), the "multi-morbidity" cluster revealed significant differences between males and females, suggesting that outside the cardio-metabolic axis, there are distinct, sex-specific factors preventing individuals form aging successfully. While male members from the "multi-morbid" cluster received the least education of all clusters (*P*<0.001) and had one of the lowest median poverty income ratios (*P*<0.001), female members of this cluster were significantly more highly educated (*P*<0.001) and had one of the highest median poverty income ratios (*P*<0.001) instead. Females from the "multi-morbid" cluster were better able to do heavy work (*P*=0.03) and participated in muscle strengthening activities (*P*<0.01), although they were not significantly better at performing functional tasks around the home. Males however were significantly less able (*P*<0.01) and unable (*P*=0.01) to perform functional tasks around the home, although they were neither significantly more sedentary nor participated less in muscle strengthening activities. While male members aged 65 and above were significantly frailer than "healthy agers" (*P*=0.02), this was not the case for females, who had similar frailty scores to "healthy agers". The male and female "multi-morbid" clusters were similar in that both had significantly more current smokers (*P*<0.001 for both), alcohol use disorder (*P*=0.01 for males and *P*=0.02 for females), and their members had the lowest BMI (*P*<0.001 for both) amongst all the clusters. The differences between male and female members of this cluster suggest that there are distinct, sex-specific trajectories that are captured. While there were significantly more males and females who were disease-free (*P*<0.001 for both), however, other members suffered from a significant variety of chronic diseases including chronic obstructive pulmonary disease (*P*=0.02 for males and *P*<0.01 for females), liver disease (*P*<0.01 for females), anemia (*P*=0.02 for females), osteoporosis and fragility fractures (*P*<0.01 for females), and cognitive impairment (*P*<0.001 for males and *P*=0.02 for females). Unlike the "cardio-metabolic" clusters, members from the "multi-morbid" clusters suffered from significantly less cardiovascular disease (*P*<0.001 for both) and obesity (*P*<0.001 for both). As expected, given the disease spectrum, there were significantly fewer male members who required treatment with an ACE-I or ARB (*P*=0.02), although this was not statistically significant for females. However, when treatment was indicated, there were significantly fewer members from the "multi-morbid" clusters who received the required chronic medications, for example, an ACE-I or ARB, at an earlier age (*P*=0.03 for 45-64 yo males, and *P*=0.02 for 45-64 yo females). There were also overall significantly more relatively younger members from the "multi-morbid" clusters who required treatment but were missed (*P*<0.01 for both 45-64 yo males and females). In general, male members of the "multi-morbid" cluster accessed healthcare less frequently, with fewer males from this cluster having visited their healthcare providers (*P*<0.001 for males who did not visit their healthcare providers at all over the past year). However, significantly more males required hospitalizations over the past year (*P*=0.03), suggesting a pattern of fewer routine visits and a higher reliance on emergency treatment. After 20 years of follow-up, fewer males than expected remained alive (*P*<0.001), and disease-specific mortality was significantly higher for cardiovascular disease (*P*<0.001 for males only), cancer (*P*<0.01 for males only), chronic lower respiratory disease (*P*=0.01 for males and *P*<0.001 for females), influenza and pneumonia (*P*<0.01 for both), and Alzheimer's dementia (*P*<0.02 for males and *P*=0.04 for females). Taken together, these results complement our findings in the "healthy aging" cluster, suggesting that lack of early, preventative, and

proactive treatment of age-related disease(s) and associated risk factors contributes to unsuccessful aging later in life.

The cluster analysis shows that individuals separated in feature space along the major PCs selected by PCAge fall into distinct patient cohorts that differ not only by life expectancy but also by socioeconomic, lifestyle and behavioral factors as well as by their medical history. This is true even though none of these factors were originally included in the model, demonstrating that the biomedical parameters informing PCAge form a complex and tightly interconnected network with many of the behavioral and lifestyle factors known to impact healthy aging.

Supplementary Table 6. PC2 and PC4 parameters with top 10% positive and negative weights.

PC2 Parameter	Weight	PC4 Parameter	Weight
Subtotal Fat (g)	0.225	Fibrinogen (g/L)	0.240
Total Fat (g)	0.224	Segmented Neutrophils Number (1000 cell/µ/L)	0.235
Left Arm Fat (g)	0.209	C-Reactive Protein (mg/dL)	0.213
Right Arm Fat (g)	0.209	Glycohemoglobin (%)	0.209
Left Leg Fat (g)	0.206	White Blood Cell Count (1000 cell/µ/L)	0.200
Right Leg Fat (g)	0.206	Glucose (mmol/L)	0.191
Trunk Fat (g)	0.206	Segmented Neutrophils Percent (%)	0.166
Trunk Percent Fat	0.203	Globulin (g/L)	0.161
Left Arm Bone Mineral Density (g/cm^2)	-0.077	Lymphocyte Percent (%)	-0.169
Right Arm Bone Mineral Density (g/cm^2)	-0.077	Iron (µmol/L)	-0.167
Albumin (g/L)	-0.066	Transferrin Saturation (%)	-0.162
Head Area (cm^2)	-0.065	Chloride (mmol/L)	-0.122
Iron (µmol/L)	-0.063	Average Diastolic Blood Pressure (mmHg)	-0.096
Transferrin Saturation (%)	-0.062	Albumin (g/L)	-0.094
Pelvis Area (cm^2)	-0.060	Maximal Calf Circumference (cm)	-0.090
Standing Height (cm)	-0.059	Sodium (mmol/L)	-0.088

Supplementary Table 7. Median and median absolute deviation (MAD) values utilized for normalization of clinical parameters, as well as 25th quartile (Q25), 75th quartile (Q75), and individual weights for parameters for LinAge.

Parameter	Male					Female				
	Median	MAD	Q25	Q75	Individual Weights	Median	MAD	Q25	Q75	Individual Weights
Chronological Age (β_{CA}) (months)	N.A.[#]	N.A.[#]	N.A.[#]	N.A.[#]	-0.0183	N.A.[#]	N.A.[#]	N.A.[#]	N.A.[#]	-0.0075
Body Mass Index (kg/m^2)	26.3	2.43	24.91	27.76	-0.0569	24.57	2.08	24.5	28.22	-0.4598
Systolic Blood Pressure (mmHg)	127	16.31	117	128	1.7638	125	20.76	116	129	1.2637
Diastolic Blood Pressure (mmHg)	75	10.38	67	75	-0.485	73	8.9	66	73	0.0068
Pulse Rate (bpm)	66	8.9	60	68	0.8006	68	8.9	64	72	1.0792
Hemoglobin (g/dL)	15.2	0.89	14.3	15.1	-0.0076	13.7	1.04	12.9	13.7	0.3208
Red Blood Cell Count (million cells/µL)	4.93	0.37	4.63	4.9	-0.3623	4.4	0.33	4.19	4.45	0.0468
Hematocrit (%)	45.1	2.67	42.4	44.7	0.0234	40.3	2.97	38	40.3	0.4149
Mean Cell Volume (fL)	91.5	3.85	88.5	91.4	0.7502	91.6	3.93	87.4	90.5	0.6093
Mean Cell Hemoglobin (pg)	31	1.48	29.8	30.9	0.6207	31.3	1.48	29.5	30.7	0.411
Mean Cell Hemoglobin Concentration (g/dL)	33.8	0.74	33.3	33.8	-0.1101	34	0.74	33.4	33.9	-0.2698
Red Cell Distribution Width (%)	12.5	0.59	12.2	12.6	1.9329	12.4	0.59	12.1	12.6	1.4199
Platelet Count (1000 cells/µL)	237	50.41	206	240	-0.4788	264	65.23	233	270	-0.1163
Mean Platelet Volume (fL)	8.2	0.89	7.7	8.2	0.8348	8.1	0.74	7.7	8.2	-0.1797
White Blood Cell Count (1000 cells/µL)	6.6	1.63	5.6	6.7	0.3903	6.3	1.63	5.7	6.9	0.3447
Segmented Neutrophils Percent (%)	59.2	8.15	52.8	58.8	0.0868	57.3	8.38	52.2	58	0.0015
Lymphocyte Percent (%)	28.3	7.41	23.15	28.6	-0.0078	31.4	7.56	25.5	30.8	0.0362
Monocyte Percent (%)	8.5	1.78	7.3	8.5	-0.3965	7.7	1.63	6.4	7.6	-0.2868
Eosinophils Percent (%)	2.5	1.33	1.7	2.7	-0.1064	2	1.19	1.5	2.2	0.419
Basophils Percent (%)	0.6	0.3	0.4	0.6	0.6472	0.6	0.3	0.4	0.6	-0.3984
Segmented Neutrophils Number (1000 cells/µL)	3.8	1.19	3.1	3.9	0.3672	3.6	1.19	3.1	4	0.2602
Lymphocyte Number (1000 cells/µL)	1.8	0.59	1.5	1.9	0.3601	1.9	0.59	1.7	2.1	0.4247
Monocyte Number (1000 cells/µL)	0.6	0.15	0.5	0.6	-0.0739	0.5	0.15	0.4	0.5	-0.0627
Basophils Number (1000 cells/µL)	N.A.[¶]	N.A.[¶]	N.A.[¶]	N.A.[¶]	0.029	N.A.[¶]	N.A.[¶]	N.A.[¶]	N.A.[¶]	-0.0024
Log C-Reactive Protein (mg/dL)	-1.97	1.03	-2.41	-1.61	0.9396	-1.56	1.1	-1.97	-1.14	0.9428
Fibrinogen (g/L)	3.4	0.61	3.12	3.58	1.0257	3.46	0.62	3.28	3.76	1.2387
Lactate Dehydrogenase (U/L)	137	25.2	121	139	0.2753	137	28.17	123	142	-0.0588
Iron (µmol/L)	16.65	5.57	12.17	16.11	0.2438	15.57	6.09	10.56	13.96	0.0743
Total Iron Binding Capacity (µmol/L)	62.47	9.01	56.39	62.47	0.2293	66.59	10.48	58.71	65.34	-0.4431
Transferrin Saturation (%)	26.5	8.9	19.5	25.9	0.189	23.55	9.12	15.9	21.4	0.2854
Ferritin (µg/L)	134	103.78	81	146	-0.3941	64.5	58.56	34	70	0.2913
Folate (nmol/L)	31	15.12	21.1	29.7	0.6654	35.3	16.75	23.1	33.3	-0.371
Vitamin B12 (pmol/L)	333.58	130.2	261.25	338.74	-0.0174	389.66	189.29	263.47	361.62	-0.0203
Blood Urea Nitrogen (mmol/L)	5.4	1.48	4.6	5.4	-0.3801	4.64	1.54	3.93	5	0.3013
Sodium (mmol/L)	139.5	2.22	137.8	139.2	-0.2775	139	2.97	137.2	139	0.5501
Potassium (mmol/L)	4.11	0.31	3.9	4.15	0.4169	4	0.3	3.8	4	0.7207
Chloride (mmol/L)	102.7	2.52	100.6	102.4	-0.47	102.6	2.52	100.6	102.6	0.3513
Bicarbonate (mmol/L)	24	2.97	23	24	-0.3404	24	1.48	22	24	0.3577
Creatinine (µmol/L)	79.6	13.2	70.7	79.6	-0.3536	61.88	13.11	53	61.88	-0.1335
Calcium Total (mmol/L)	2.35	0.07	2.3	2.35	0.4501	2.35	0.11	2.28	2.35	-0.0054
Phosphorus (mmol/L)	1.1	0.15	1	1.1	0.1301	1.16	0.14	1.07	1.16	0.1654
Protein Total (g/L)	74	4.45	71	74	0.3662	74	4.45	71	74	-0.9744
Albumin (g/L)	44	2.97	42	44	-0.4833	43	2.97	41	42	-0.6401
Globulin (g/L)	30	4.45	28	30	0.6748	30	4.45	29	31	-0.5466
Bilirubin (µmol/L)	11.97	2.56	10.26	11.97	0.1548	10.26	2.54	6.8	10.26	-0.7655
Alkaline Phosphatase (IU/L)	70	20.76	60	72	-0.1903	67	19.27	57	73	0.4288
Alanine Aminotransferase (U/L)	24	7.41	19	24	-1.6351	18	5.93	15	19	-1.0532
Aspartate Aminotransferase (U/L)	24	5.93	20	24	1.1366	22	5.93	18	21	1.5262
Gamma Glutamyl Transferase (U/L)	25	11.86	19	27	0.1972	18	7.41	14	20	0.0745
Uric Acid (µmol/L)	345	79.32	303.3	350.9	0.6415	261.7	61.68	237.9	285.5	0.9131
Glucose (mmol/L)	5.16	0.49	4.88	5.22	0.3585	4.91	0.45	4.72	5.05	0.4584
Glycohemoglobin (%)	5.4	0.3	5.2	5.5	0.044	5.3	0.3	5.2	5.4	0.1086
Total Cholesterol (mmol/L)	5.33	0.92	4.64	5.25	0.3847	5.38	0.85	4.86	5.48	-0.151

High-Density Lipoprotein (mmol/L)	1.17	0.31	0.96	1.14	0.5512	1.5	0.39	1.16	1.41	0.9171
Triglycerides (mmol/L)	1.42	0.75	1.02	1.48	1.1103	1.2	0.6	0.96	1.37	-0.5964
Low-Density Lipoprotein (mmol/L)	3.76	0.87	3.09	3.69	0.0337	3.53	0.78	3.07	3.68	-0.3884
Log N-Terminal Pro-Brain Natriuretic Peptide (pg/mL)	3.81	1.19	3.12	3.95	1.7226	4.3	0.91	3.8	4.42	1.7577
Urine ACR (mg/g)	5.3	3.15	4.02	6.46	0.001	7.1	4.7	5	7.95	0.0044
Smoking status / Cotinine	N.A.*	N.A.*	N.A.*	N.A.*	0.9828	N.A.*	N.A.*	N.A.*	N.A.*	1.1906
Co-morbidity index	N.A.#	N.A.#	N.A.#	N.A.#	0.0492	N.A.#	N.A.#	N.A.#	N.A.#	0.0717
Self-health index	N.A.#	N.A.#	N.A.#	N.A.#	0.6674	N.A.#	N.A.#	N.A.#	N.A.#	1.3017
Healthcare use index	N.A.#	N.A.#	N.A.#	N.A.#	-0.048	N.A.#	N.A.#	N.A.#	N.A.#	0.9043
C_0 Constant	N.A.#	N.A.#	N.A.#	N.A.#	7.46	N.A.#	N.A.#	N.A.#	N.A.#	-1.71

N.A. = not applicable

¶ N.A. because the median and MAD were 0, hence, actual Basophils Number were used instead

* N.A. because smoking status was determined by using actual serum cotinine levels organized into bins – 0-10 ng/mL (non-smokers), 10-99 ng/mL (light smokers), 100-199 (moderate smokers), and ≥ 200 (heavy smokers) – which could be replaced by questionnaire data if cotinine data are not available

N.A. because actual scores were used

Supplementary Table 8. Differences between the NHANES III and IV cohorts.

	NHANES III	NHANES IV
Directors / PI	• Robert S. Murphy (1979-1997)	• Raynard S. Kington (1999-2001)<br>• Clifford L. Johnson (2001-2012)<br>• Kathryn S. Porter (2013-2019)
Goals	• Estimate national prevalence of selected diseases and risk factors<br>• Estimate national population reference distributions of selected health parameters<br>• Document and investigate reasons for secular trends in selected diseases and risk factors<br>• Contribute to understanding of disease etiology<br>• Investigate natural history of selected diseases	• Provide prevalence data on selected diseases and risk factors for US population<br>• Monitor trends in selected diseases, behaviors, and environmental exposures<br>• Explore emerging public health needs<br>• Maintain a national probability sample of baseline information on health and nutritional status
Cohort	• 1988-1994<br>• 81 counties across US<br>• 33,994 interviewed<br>• 30,818 examined<br>• 2 months and over<br>• Focused on subpopulation nutrition and health<br>• Oversampled children aged 2 months to 5 years, persons 60 years and older (8,200), Mexican-Americans, and non-Mexican-American blacks<br>◦ "Survey content of NHANES III is particularly useful for the study of the contribution of multiple diseases to disability in old age", including "cardiovascular disease, pulmonary disease, diabetes, osteoarthritis, and osteoporosis"	• Continuous since 1999<br>• 5,000 individuals (except institutionalized persons) per year from 15 different county locations selected from a sampling frame that included all 50 states hence representative of entire US population<br>• Oversampled subgroups for 1999-2006 included non-Hispanic blacks, Mexican-Americans, low-income white persons (beginning in 2000), adolescents 12-19 years, and persons aged 70 and over<br>• 1999-2000: 9,965 interviewed, 9,282 examined<br>• 2001-2002: 11,039 interviewed, 10,477 examined
Location	• Staff / Team<br>◦ Physician (medical exam)<br>◦ Dentist<br>◦ Health interviewer (questionnaires)<br>◦ Dietary interviewers<br>◦ Ultrasound technician<br>◦ Health technicians (exam)<br>◦ Medical technologists (processed biological samples)<br>◦ Phlebotomist (drew blood)<br>• Household interviews<br>◦ Questionnaires<br>• Mobile examination center (trailers)<br>◦ Exam (e.g. BP, DXA, Ultrasound, Spirometry, X-ray)<br>◦ Biospecimen collection (e.g. blood, urine)<br>◦ Lab tests (e.g. CBC)<br>• Home exam<br>◦ Exam for older persons at home who are unable or unwilling to come to the MEC for a complete exam	• Staff / Team<br>◦ Manager<br>◦ Coordinator<br>◦ Physician (medical exam, medical emergencies)<br>◦ Health interviewers (questionnaires)<br>◦ Dietary interviewers (24-hour diet recall)<br>◦ Health technologists (exam)<br>◦ Medical technolo30gists (processed biological specimens, conducted CBC, prepared specimens for shipment to labs across US)<br>◦ Phlebotomist (drew blood)<br>• Household interviews<br>◦ Questionnaires<br>• Mobile examination center (trailers)<br>◦ Exam (e.g. BP, DXA)<br>◦ Biospecimen collection (e.g. blood, urine)<br>◦ Lab tests (e.g. CBC)
Technology	<u>Lab or Diagnostic Center</u><br>• Lipids – Lipoprotein Analytical Laboratory, Johns Hopkins University<br>• Urine albumin (micro)/creatinine – Department of Pediatrics, University of Minnesota<br>• Glucose, HbA1c – Department of Child Health, University of Missouri-Columbia<br>• CRP – Immunology Division, University of Washington<br>• Biochemistry profile, Fibrinogen – White Sands Research Center, Alamogordo, NM<br>• Nutrition biochemistries – National Center for Environmental Health, CDC, Atlanta, GA<br>• WBC differential – National Center for Infectious Diseases, CDC, Atlanta, GA<br>• CBC – NHANES III MEC<br><br><u>Analysis / Analyzer</u><br>• CBC, Platelets, 3-cell differential, RDW – Coulter S-PLUS JR Blood Analyzer<br>• Differential smears – Manual differential on abnormals and 10% of normals<br>• HbA1c – DIAMAT HPLC/Bio-Rad Laboratories<br>• Folate - "Quantaphase Folate" RIA Kit/Bio-Rad Laboratories<br>• Iron, TIBC – Alpkem RFA Automated Ferrozine Colorimetric<br>• Ferritin – Quantimune Ferritin IRMA Kit/Bio-Rad Laboratories<br>• B12 – ^{125}I-folic/^{57}Co-B-12<br>• Total cholesterol, HDL, Triglycerides – Hitachi 704 Analyzer/Boehringer-Mannheim Diagnostics	<u>Lab or Diagnostic Center</u><br>• Cotinine – Organic Analytical Toxicants Branch, Division of Laboratory Sciences, National Center for Environmental Health, CDC<br>• Ferritin, Folate, B12, Iron, TIBC – Inorganic Toxicology and Nutrition Branch, Division of Laboratory Sciences, National Center for Environmental Health, CDC<br>• Glucose, HbA1c – Department of Child Health, University of Missouri-Columbia<br>• CRP, Fibrinogen – Immunology Division, University of Washington<br>• Lipids – Lipoprotein Analytical Laboratory, Johns Hopkins University<br>• Urine creatinine – University of Minnesota<br>• Biochemistry profile – Coulston Foundation, Alamogordo, NM<br>• CBC with 5-part differential – NHANES IV MEC<br><br><u>Analysis / Analyzer</u><br>• Cotinine – ID HPLC-APCI MS/MS (HPLC Hewlett-Packard model 1090L, Series II. MS PE-Sciex API III Triple Quadrupole mass spectrometer with heated nebulizer interface)<br>• Ferritin – Quantimune Ferritin IRMA Kit/Bio-Rad Laboratories<br>• Folate, B12 - "Quantaphase II Folate/vitamin B12" radioassay kit/Bio-Rad Laboratories<br>• Iron, TIBC – Modification of the automated AAII-25 colorimetric method

	• Total calcium – NOVA 7+7 Electrolyte Analyzer/NOVA Biomedical<br>• Cotinine – EIA Screen/STC, Inc., LCMS Confirmation/Perkin-Elmer SCIEX<br>• CRP – Behring Nephalometric Analyzer/Behring<br>◦ Lower limit of detection 0.021<br>• Biochemistry profile – Hitachi 737 Analyzer/Boehringer-Mannheim Diagnostics<br>• Fibrinogen – Coag-A-Mate XC Plus/Organon-Teknika/General Diagnostics<br>• Urine creatinine – Synchron AS/ASTRA Clinical Analyzer/Beckman Instruments<br>• Urine albumin (micro) – Fluorescent Immunoassay/Bio-Rad Laboratories	• HbA1c – Primus Automated HPLC System (Primus I, Model CLC330)<br>• Glucose – Enzyme hexokinase<br>• CRP – Latex-enhanced Nephelometry<br>• Fibrinogen – Rate of Clot Formation on the STA-Compact<br>• Lipids – Hitachi 704 Analyzer/Roche Diagnostics (formerly Boehringer-Mannheim Diagnostics)<br>• Urine creatinine – Synchron CX3 Clinical Analyzer/Beckman Instruments<br>• Urine albumin – Fluorescent Immunoassay using Fluorometer, Sequoia-Turner Digital Model 450<br>• Biochemistry profile – Hitachi Model 917 Multichannel Analyzer/Roche Diagnostics (formerly Boehringer-Mannheim Diagnostics)<br>• CBC with 5-part differential – Beckman Coulter MAXM

Code and Supplementary Files

1. **PCAge construction R script (PCAgeScript.R)**

   This is a version of the original R script for the construction of PCAge, including all steps of data reduction and normalization, dimensionality reduction, model fitting and application. The script is modularized and commented, with an intention to be instructive rather than elegant or efficient. The script should be run using R and expects the relevant NHANES input data (nhanesMerged.csv) and the “codebook” file (codebook.csv) in the same working directory.

2. **NHANES example input data file (nhanesMerged.csv)**

   Example data comprising publicly available data from the NHANES IV recruitment waves from 1999 to 2002. Biological parameters are in columns and subjects are in rows. Variable (column) names are harmonized using those of the 1999/2000 wave. Mortality follow-up has been integrated into the file (variables: MORTSTAT, UCOD_LEADING, PERMTH_INT, ELIGSTAT).

3. **Codebook and feature selection file (codebook.csv)**

   This file contains information on the relevant NHANES variable terms (Var). For each variable, the file contains a short human readable explanation (Human) and a flag indicating the type of variable (DEMO: demographic, Q: questionnaire, E: physical exam, LAB: laboratory parameter, MORTALITY: survival follow-up). The “Data” flag indicates numerical data while the “ForceInc” flag forces the variable to be used in the PCAge model, regardless of NA numbers. The force include and data flags can be modified to construct custom clocks using PCAgeScript.R.

4. **LinAge spreadsheet implementation (linAge_Example.xls)**

   The folder contains a readme file with instructions (README.txt), and a spreadsheet version of LinAge (linAge_Example.xls). The spreadsheet comprises three tabs. The example_male contains data on a single (male) example subject (NHANES subject with NHANES SEQN = 10). The parameters_male and parameters_female tabs contain the final LinAge parameter values for the male and female LinAge models.

5. **LinAge R script (linAge.R)**

   This folder contains a standalone R script (linAge.R) for calculation of LinAge from model parameters (linAge_Paras.csv) and input data in NHANES IV format. We have also included examples from NHANES IV containing all required input data (dataMat_test.csv) and the associated demographic and questionnaire data (qDataMat_test.csv). All four files need to be in the same R directory to run.

6. **Custom PC Clock R script (customClock_script.R)**

   The customClock folder contains a standalone R script (customClock_script.R) that runs through all the steps for generating and testing a PCA clock, using a custom feature set selected from NHANES IV. The script will use the 1999/2000 recruitment wave of NHANES IV to build a custom clock before testing the clock in the separate 2001/2002 recruitment wave. The clock is constructed using a user-defined set of features and some user-modifiable parameters. Features are selected for inclusion by editing the codebook file (codebook_custom_linAge.csv). Parameters controlling different aspects of this workflow can also be set in a parameter file (paraInit.csv). See the README.txt for more detailed instructions. All required files can be found in this folder.
