# Supplementary material for: Principal component-based clinical aging clocks identify signatures of healthy aging and targets for clinical intervention
Source: Nat Aging. 2024 Jun 19;4(8):1137–52. doi: 10.1038/s43587-024-00646-8 (PMC11333290; doi:10.1038/s43587-024-00646-8)
Supplement: Supplementary file 2 — Reporting Summary [file 43587_2024_646_MOESM2_ESM.pdf]

Corresponding author(s): Jan Gruber

Last updated by author(s): Apr 20, 2024

Reporting Summary

Nature Portfolio wishes to improve the reproducibility of the work that we publish. This form provides structure for consistency and transparency in reporting. For further information on Nature Portfolio policies, see our Editorial Policies and the Editorial Policy Checklist.

Statistics

For all statistical analyses, confirm that the following items are present in the figure legend, table legend, main text, or Methods section.

n/a	Confirmed	
☐	☒	The exact sample size (*n*) for each experimental group/condition, given as a discrete number and unit of measurement
☐	☒	A statement on whether measurements were taken from distinct samples or whether the same sample was measured repeatedly
☐	☒	The statistical test(s) used AND whether they are one- or two-sided<br>*Only common tests should be described solely by name; describe more complex techniques in the Methods section.*
☐	☒	A description of all covariates tested
☐	☒	A description of any assumptions or corrections, such as tests of normality and adjustment for multiple comparisons
☐	☒	A full description of the statistical parameters including central tendency (e.g. means) or other basic estimates (e.g. regression coefficient) AND variation (e.g. standard deviation) or associated estimates of uncertainty (e.g. confidence intervals)
☐	☒	For null hypothesis testing, the test statistic (e.g. *F*, *t*, *r*) with confidence intervals, effect sizes, degrees of freedom and *P* value noted<br>*Give P values as exact values whenever suitable.*
☒	☐	For Bayesian analysis, information on the choice of priors and Markov chain Monte Carlo settings
☐	☒	For hierarchical and complex designs, identification of the appropriate level for tests and full reporting of outcomes
☐	☒	Estimates of effect sizes (e.g. Cohen's *d*, Pearson's *r*), indicating how they were calculated

Our web collection on statistics for biologists contains articles on many of the points above.

Software and code

Policy information about availability of computer code

Data collection	All data used in this study were obtained from publicly available sources<br>- For the National Health and Nutrition Examination Survey (NHANES) IV cohort, data were retrieved from the NHANES website (https://www.cdc.gov/nchs/nhanes/index.htm) using the nhanesA R package.<br>- For the NHANES III cohort, data were retrieved from the NHANES website (https://wwwn.cdc.gov/nchs/nhanes/nhanes3/datafiles.aspx#core).<br>- For the Comprehensive Assessment of Long-term Effects of Reducing Intake of Energy (CALERIE) trial, data were retrieved from the CALERIE trial website (https://calerie.duke.edu).
Data analysis	All statistical analyses were performed using R version 4.2.0 (https://www.R-project.org/). R packages were used for regularized Cox regression (glmnet version 4.1-7 and survival package version 3.5-8), for k-means clustering (cluster version 2.1.4 and factoextra version 1.0.7), for partial correlation network analysis (ppcor version 1.1 and igraph version 2.0.3), and for PCA imputation (missMDA version 1.18).

For manuscripts utilizing custom algorithms or software that are central to the research but not yet described in published literature, software must be made available to editors and reviewers. We strongly encourage code deposition in a community repository (e.g. GitHub). See the Nature Portfolio guidelines for submitting code & software for further information.

Data

Policy information about availability of data

All manuscripts must include a data availability statement. This statement should provide the following information, where applicable:

- Accession codes, unique identifiers, or web links for publicly available datasets
- A description of any restrictions on data availability
- For clinical datasets or third party data, please ensure that the statement adheres to our policy

All datasets utilized are publicly available online at: https://wwwn.cdc.gov/nchs/nhanes/Default.aspx, https://wwwn.cdc.gov/nchs/nhanes/nhanes3/datafiles.aspx#core, and https://calerie.duke.edu. No new datasets were generated in this study. No software was used for data collection.

Research involving human participants, their data, or biological material

Policy information about studies with human participants or human data. See also policy information about sex, gender (identity/presentation), and sexual orientation and race, ethnicity and racism.

Reporting on sex and gender	Data on gender of the sample person were obtained using the code "RIAGENDR" from the demographics dataset of NHANES IV and the code "HSSEX" from the Household Adult File of NHANES III. Data obtained were self-reported. Separate PCAge biological aging clocks were constructed for males and females. Our final training cohort, composed of the NHANES 1999-2000 study participants, included 923 males and 852 females, while our testing cohort, composed of the NHANES 2001-2002 study participants, included 1,094 males and 942 females. Subsequent downstream analyses were performed separately on males and females. Separate LinAge biological aging clocks were also constructed for males and females in NHANES IV and then externally validated in NHANES III (715 males and 819 females).
Reporting on race, ethnicity, or other socially relevant groupings	Data on race/ethnicity were obtained using the code "RIDRETH1" from the demographics dataset of NHANES IV. Data coded in this variable is self-reported.
Population characteristics	The baseline characteristics of the participants from NHANES IV are reported in Supplementary Table 2.
Recruitment	The authors of this study were not directly involved in any of these trials - data was exclusively obtained from publicly available / previously published sources.<br><br>The continuous NHANES IV is an ongoing cohort study, by the National Center for Health Statistics, designed to assess the health and nutritional status of a nationally representative population of adults in the United States. The study involves a series of cross-sectional surveys that includes information on demographic, socioeconomic, dietary, health-related questions, medical and physiological measurements, as well as laboratory tests.<br><br>The NHANES III study was conducted from 1988-1994 by the National Center for Health Statistics to assess the health and nutritional status of the United States' civilian, non-institutionalized population. Cross-sectional survey data included demographic, socioeconomic, dietary, health-related questions, medical examination, physiological measurements, and clinical laboratory tests.<br><br>The CALERIE trial was conducted at three clinical centers in the United States that randomly assigned 220 healthy non-obese male and female volunteers between the ages 20-50 years at a ratio of 2:1 to either caloric-restricted (CR) or ad libitum (AL) control groups.
Ethics oversight	The NHANES IV and III cohorts are approved by the National Center for Health Statistics Research Ethics Review Board. The CALERIE trial received ethics approval at three clinical centers (Washington University School of Medicine, St Louis, MO, USA; Pennington Biomedical Research Center, Baton Rouge, LA, USA; Tufts University, Boston, MA, USA) and the coordinating center at Duke University (Durham, NC, USA).

Note that full information on the approval of the study protocol must also be provided in the manuscript.

Field-specific reporting

Please select the one below that is the best fit for your research. If you are not sure, read the appropriate sections before making your selection.

☒ Life sciences ☐ Behavioural & social sciences ☐ Ecological, evolutionary & environmental sciences

For a reference copy of the document with all sections, see nature.com/documents/nr-reporting-summary-flat.pdf

Life sciences study design

All studies must disclose on these points even when the disclosure is negative.

Sample size	For all three cohorts, no statistical method was used to predetermine sample size. For the NHANES IV cohort, we excluded: (1) participants top-coded at age 85 years as we could not ascertain the exact CAs of these adults, (2) participants who died from accidental deaths as these

	were deemed to be not age-related, and (3) physiological and laboratory measurements with significant missing data, defined as more than 10% of the training dataset. For the NHANES III cohort, we excluded: (1) participants top-coded at age 90 years as we could not ascertain the exact CAs of these adults, (2) participants who died from accidental deaths as these were deemed to be not age-related, and (3) subjects for whom laboratory measurements needed to calculate LinAge were missing. For the CALERIE cohort, participants with missing data for whom CALinAge could not be calculated were excluded.<br><br>After excluding the participants as described above, for the NHANES IV 1999-2002 recruitment waves, data were available for 3,811 participants; for the NHANES III external validation cohort, data were available for 1,534 participants; and, for the CALERIE trial, data were available for 159 participants. These sample sizes were sufficient to generate the PCAge, LinAge and CALinAge clocks, as evidenced by ROC and stratification of survival analysis.
Data exclusions	For the NHANES IV 1999-2002 cohorts, we excluded: (1) participants top-coded at age 85 years as we could not ascertain the exact chronological ages of these adults, (2) participants who died from accidental deaths as these were deemed to be not age-related, and (3) physiological and laboratory measurements with significant missing data, defined as more than 10% of the training dataset.<br><br>For the NHANES III cohort, we excluded: (1) participants top-coded at age 90 years as we could not ascertain the exact CAs of these adults, (2) participants who died from accidental deaths as these were deemed to be not age-related, and (3) subjects for whom laboratory measurements needed to calculate LinAge were missing.<br><br>For the CALERIE trial, participants with missing data for whom CALinAge could not be calculated were excluded.
Replication	We trained the PCAge and LinAge clocks in the NHANES IV 1999-2000 recruitment wave, and then tested PCAge and LinAge in a completely separate testing cohort, extracted from the NHANES IV 2001-2002 recruitment wave. We further validated LinAge in an external validation cohort, extracted from NHANES III, finding that it performed equally well in predicting survival. Finally, we validated our PCA approach by applying the same procedure outline for PCAge/LinAge to train and validate a mortality clock in NHANES IV. The resulting "CALinAge" custom clock could predict mortality differences in the NHANES IV 2001-2002 test cohort for both sexes within the age range relevant for CALERIE. All attempts at replication of PCAge, LinAge and CALinAge were successful.
Randomization	Where needed for statistical testing, we carried out random sampling using the random number generator functions for normal or uniform distributed random numbers. Details on this is given in methods where appropriate. For construction of the clocks, we included the whole training and testing cohort (separate NHANES IV recruitment years) to train and evaluate (respectively), without any further sub-selection of subjects.<br><br>For the CALERIE phase 2 randomized controlled trial, participants were randomly assigned, using a permuted block randomization technique, at a 2:1 ratio to either CR or AL groups. Randomization was stratified by site, sex, and BMI.
Blinding	For NHANES IV and III, blinding was not relevant because these are cohort studies for which data have already been collected and processed. We used publicly available data, applying machine learning and statistical techniques to these cohorts.<br><br>For the CALERIE trial, all study personnel were blinded.

Reporting for specific materials, systems and methods

We require information from authors about some types of materials, experimental systems and methods used in many studies. Here, indicate whether each material, system or method listed is relevant to your study. If you are not sure if a list item applies to your research, read the appropriate section before selecting a response.

Materials & experimental systems

n/a	Involved in the study
☒	☐ Antibodies
☒	☐ Eukaryotic cell lines
☒	☐ Palaeontology and archaeology
☒	☐ Animals and other organisms
☐	☒ Clinical data
☒	☐ Dual use research of concern
☒	☐ Plants

Methods

n/a	Involved in the study
☒	☐ ChIP-seq
☒	☐ Flow cytometry
☒	☐ MRI-based neuroimaging

Clinical data

Policy information about clinical studies

All manuscripts should comply with the ICMJE guidelines for publication of clinical research and a completed CONSORT checklist must be included with all submissions.

Clinical trial registration	Not available for the NHANES IV and III cohorts. The CALERIE trial is registered on ClinicalTrials.gov as NCT00427193.
Study protocol	The NHANES IV study protocol is available online at https://wwwn.cdc.gov/nchs/nhanes/Default.aspx. The NHANES III study protocol is available online at https://wwwn.cdc.gov/nchs/nhanes/nhanes3/surveymethods.aspx. The CALERIE trial study protocol is available online at https://calerie.duke.edu/protocols-procedures.

Data collection

The continuous NHANES IV is an ongoing cohort study, by the National Center for Health Statistics, designed to assess the health and nutritional status of a nationally representative population of adults in the United States. The study involves a series of cross-sectional surveys that includes information on demographic, socioeconomic, dietary, health-related questions, medical and physiological measurements, as well as laboratory tests. Survey data are frequently updated online at https://wwwn.cdc.gov/nchs/nhanes/Default.aspx. For the NHANES 1999-2002 recruitment waves, participants were recruited and data collected from the years 1999-2002.

The NHANES III study was conducted from 1988-1994 by the National Center for Health Statistics to assess the health and nutritional status of the United States' civilian, non-institutionalized population. Cross-sectional survey data included demographic, socioeconomic, dietary, health-related questions, medical examination, physiological measurements, and clinical laboratory tests. Survey data are available online at https://wwwn.cdc.gov/nchs/nhanes/nhanes3/datafiles.aspx#core. For NHANES III, participants were recruited and data collected from the years 1988-1994.

CALERIE was conducted at three clinical centers (Washington University School of Medicine, St Louis, MO, USA; Pennington Biomedical Research Center, Baton Rouge, LA, USA; Tufts University, Boston, MA, USA) in the United States that randomly assigned 220 healthy non-obese male and female volunteers between the ages 20-50 years at a ratio of 2:1 to either caloric-restricted or ad libitum control groups. Data from CALERIE are available online at https://calerie.duke.edu. The CALERIE trial started in May 2007 and completed in November 2012.

Outcomes

This study leverages on a data-driven approach to construct clinically actionable biological aging clocks generated against mortality as the primary outcome. Linked mortality data for NHANES IV and III were obtained from the National Death Index, and available from Oct 18, 1988 until Dec 31, 2019 for NHANES III, and from Jan 1, 1999 until Dec 31, 2019 for NHANES IV. Secondary outcomes are not applicable to this study, which focused on the analysis of publicly available datasets.

Plants

Seed stocks

Report on the source of all seed stocks or other plant material used. If applicable, state the seed stock centre and catalogue number. If plant specimens were collected from the field, describe the collection location, date and sampling procedures.

Novel plant genotypes

Describe the methods by which all novel plant genotypes were produced. This includes those generated by transgenic approaches, gene editing, chemical/radiation-based mutagenesis and hybridization. For transgenic lines, describe the transformation method, the number of independent lines analyzed and the generation upon which experiments were performed. For gene-edited lines, describe the editor used, the endogenous sequence targeted for editing, the targeting guide RNA sequence (if applicable) and how the editor was applied.

Authentication

Describe any authentication procedures for each seed stock used or novel genotype generated. Describe any experiments used to assess the effect of a mutation and, where applicable, how potential secondary effects (e.g. second site T-DNA insertions, mosiacism, off-target gene editing) were examined.
